# Repeated adaptive divergence of microhabitat specialization in avian feather lice

**DOI:** 10.1186/1741-7007-10-52

**Published:** 2012-06-20

**Authors:** Kevin P Johnson, Scott M Shreve, Vincent S Smith

**Affiliations:** 1 Illinois Natural History Survey, University of Illinois, Champaign, IL, USA; 2 Department of Entomology, University of Illinois, Urbana, IL, USA; 3 The Natural History Museum, London, UK

**Keywords:** adaptive radiation, convergence, Phthiraptera, ectoparasites, phylogenetics

## Abstract

**Background:**

Repeated adaptive radiations are evident when phenotypic divergence occurs within lineages, but this divergence into different forms is convergent when compared across lineages. Classic examples of such repeated adaptive divergence occur in island (for example, Caribbean *Anolis *lizards) and lake systems (for example, African cichlids). Host-parasite systems in many respects are analogous to island systems, where host species represent isolated islands for parasites whose life cycle is highly tied to that of their hosts. Thus, host-parasite systems might exhibit interesting cases of repeated adaptive divergence as seen in island and lake systems.

The feather lice of birds spend their entire life cycle on the body of the host and occupy distinct microhabitats on the host: head, wing, body and generalist. These microhabitat specialists show pronounced morphological differences corresponding to how they escape from host preening. We tested whether these different microhabitat specialists were a case of repeated adaptive divergence by constructing both morphological and molecular phylogenies for a diversity of avian feather lice, including many examples of head, wing, body and generalist forms.

**Results:**

Morphological and molecular based phylogenies were highly incongruent, which could be explained by rampant convergence in morphology related to microhabitat specialization on the host. In many cases lice from different microhabitat specializations, but from the same group of birds, were sister taxa.

**Conclusions:**

This pattern indicates a process of repeated adaptive divergence of these parasites within host group, but convergence when comparing parasites across host groups. These results suggest that host-parasite systems might be another case in which repeated adaptive radiations could be relatively common, but potentially overlooked, because morphological convergence can obscure evolutionary relationships.

## Background

Adaptive radiations are believed to be responsible for the diversification of many groups of organisms [1,2]. One common feature of groups that have diversified by an adaptive radiation is divergence of morphological features with ecological function [2,3]. In any adaptive radiation, there are two broad patterns by which lineages could diversify. On the one extreme, the adaptive morphological traits of the radiation could evolve early on in the diversification process and lineages possessing these features could then radiate in "open niches." Alternatively, divergence in morphology may be repeated and ongoing during the entire diversification process through processes of character displacement (that is, ecological speciation, [3,4]).

In the case of some adaptive radiations where there is repeated morphological diversification, this divergence in morphology may also be convergent (termed "replicate adaptive radiation", [5]). That is, different clades of the adaptive radiation converge on similar solutions to common ecological problems [4]. Several examples of potential repeated adaptive radiations have been identified, including benthic and limnetic sticklebacks [6], ecomorphs of Hawaiian *Tetragnatha *spiders [7], African rift lake cichlid fishes [8] and Bonin island land snails [9]. A particularly well-documented example is the *Anolis *lizards on large Caribbean islands, which have diversified into a number of ecomorphs related to the microhabitats in which different species occur [5,10]. However, when compared *across *different islands, these ecomorphs are convergent. That is, similar selection pressures on different islands have resulted in similarities in the type of radiations that have occurred. Such convergence suggests there might be a limited number of "niches" into which lineages can diversify. In contrast, morphological or behavioral traits under more limited ecological selection, for example, sexually selected traits, might be more likely to diversify into a broader array of forms and show less propensity for convergence [11].

It is unclear how common such repeated adaptive divergence processes are across the array of adaptive radiations in nature. Because convergent evolution leads to broad similarity in morphological features, classifications and phylogenies based mainly on morphology make identification of such convergence in a single group more difficult. By reconstructing the evolution of various ecomorphs or niche specialists over an independent phylogenetic tree, it is possible to identify the relative contributions of early versus repeated morphological divergence. In the former case, morphological characters should be highly concordant with the phylogeny, while in the latter, considerable homoplasy in morphological characters related to the adaptive features should be observed. In general, Losos [5] suggests replicate adaptive radiations are relatively rare, and typically confined to island [5,7,9,10] or lake [6,8] systems, perhaps because environments are discrete, isolated and replicated across space. Host-parasite systems have been compared as analogous to island systems [12,13], and might prove to be another case where repeated adaptive divergence is relatively more frequent.

**Figure 1:**
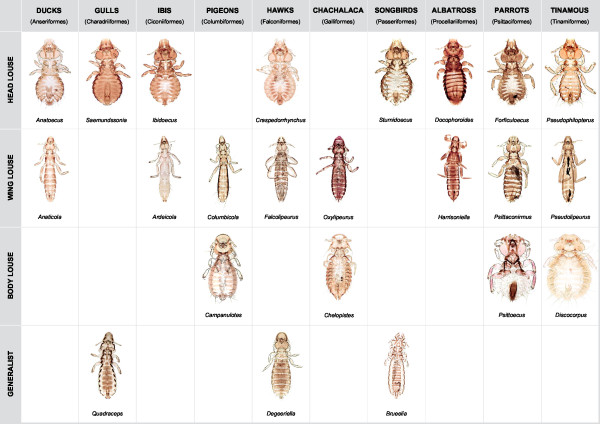
**Body forms of microhabitat specialists across diversity of avian feather lice included in this study**. Host group and microhabitat are indicated.

One group that has undergone an extensive adaptive radiation is the feather lice (Phthiraptera: Ischnocera) of birds (Aves). Feather lice are wingless ectoparasites that consume the downy portions of the feathers, and thus do not interact with the host's immune system [14]. These lice have radiated across the diversity of birds into over 2,700 species in around 140 genera [15], with pronounced morphological variation (Figure 1). The main way that birds control these ectoparasites is through preening. Feather lice have a limited number of options to escape this preening defense behavior, and these escape behaviors are highly correlated with louse morphology, suggesting strong selection on louse morphology for escape.

Feather lice generally follow one of four escape mechanisms corresponding to the main regions of the hosts body in which these escape mechanisms are used: wing lice, body lice, head lice and generalists. Avian wing lice have a long and slender body form (Figure 1) and escape from host preening by inserting themselves between the feather barbs of the primary wing feathers [16]. The size of wing lice is strongly correlated with the space between these feather barbs [17]. Body lice use more active escape mechanisms by burrowing in the downy portions of the body feathers or dropping from feather to feather [18]. These lice have a characteristic short, rounded body form with a rounded head margin. Head lice escape from host preening defenses by remaining on the head where birds cannot preen with their bills. The fact that these lice remain on the head to escape preening is also supported by the fact that these lice are not cryptically colored on birds that have cryptically colored wing and body lice [19]. Head lice have a rounded body form and a triangular head margin. These lice have a rostral groove on the head through which they insert a feather barb, gripping it with their mandibles [20]. This likely allows them to resist being removed from the bird by scratching, which is the main defense of birds against head lice [18,21]. Finally, lice that are generalists can be found over most of the bird's body, wings and sometimes head, and likely escape preening by running through the feathers [22]. These lice have an intermediate body shape and typically a rounded head margin. Although there is some behavioral plasticity in the lice of these different forms, such that sometimes lice of one microhabitat preference are found on other parts of the host's body [23], the strong correlation of overall morphological body form with these microhabitat preferences is pronounced [20,22].

Many groups of birds are host to more than one type of microhabitat specialist louse [15]. Genera of ischnoceran feather lice often correspond to lice of a single microhabitat specialization on an order of family of birds [20]. Thus the radiation of feather lice relates to both the radiation across different host groups and radiation into different microhabitats for escaping host preening defenses. Here we evaluate the pattern of diversification of this adaptive radiation. The central question is what are the relative contributions of 1) early microhabitat specialization and subsequent radiation across birds versus 2) repeated adaptive divergence [5] of microhabitat specialists within major groups of birds? We do this by reconstructing phylogenetic trees based on both molecular and morphological data sets, evaluating the level of congruence of these trees, and reconstructing microhabitat specialization over them. In particular, using randomizations, we evaluate the degree of signal contained in microhabitat specialization and host group over both the molecular and morphological trees. We evaluate the pattern of repeated adaptive divergence over each tree by determining the number of times parasites from the same host group, but of different microhabitat specializations, are sister taxa and compare this value to that expected by chance.

## Results

**Figure 2:**
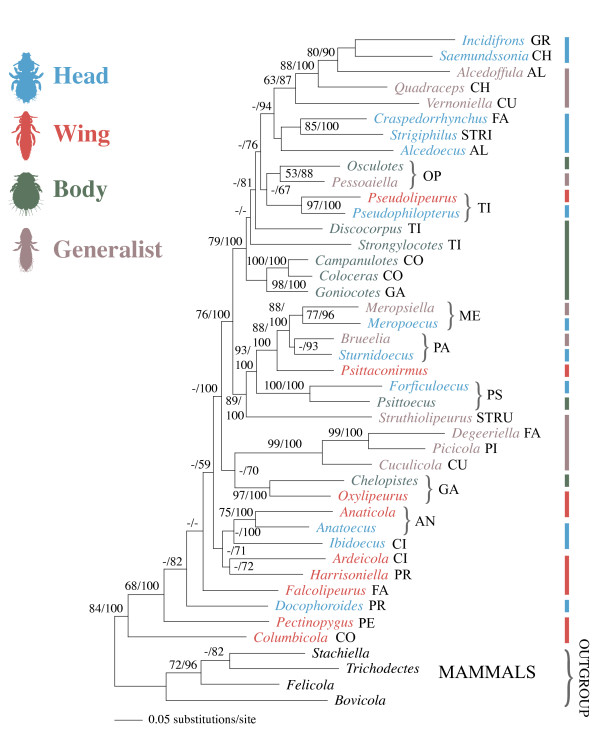
**Phylogeny based on combined maximum likelihood analyses of COI, EF-1α and Wingless gene sequences**. The tree from gene-partitioned Bayesian analysis was identical in topology. Maximum likelihood bootstrap/Bayesian posterior probabilities shown for each node when greater than 50%. Values less than 50% indicated by -. Microhabitat specialization indicated by vertical bars. Brackets indicate terminal sister pairs of genera from the same host group but of different microhabitation specializations. Avian host group indicated using the first two to four letters of the host order or family (see Table 1).

**Figure 3:**
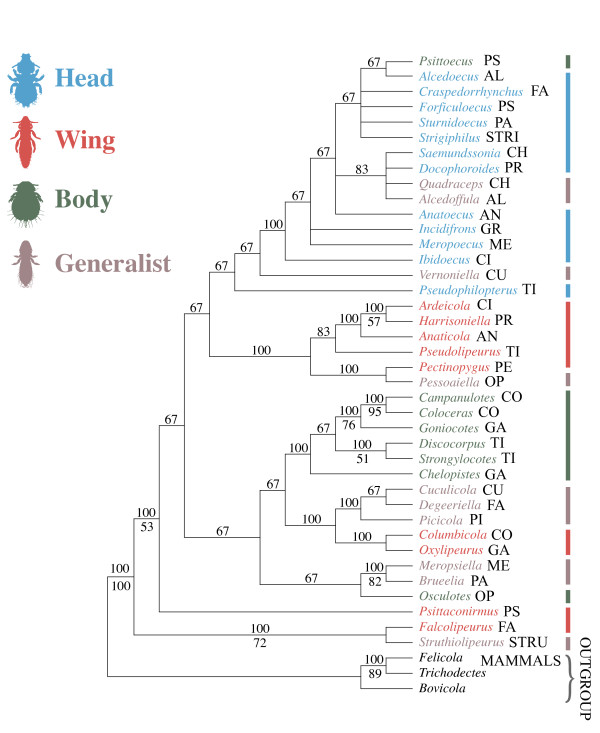
**Majority rule consensus of six equally parsimonious trees from analysis of 138 morphological characters**. Numbers above branches indicate percentage of trees for which branch is recovered. Numbers below branches indicated bootstrap values from 1,000 bootstrap replicates. Microhabitat specialization indicated by vertical bars. Avian host group indicated using the first two to four letters of the host order or family (see Table 1).

**Figure 4:**
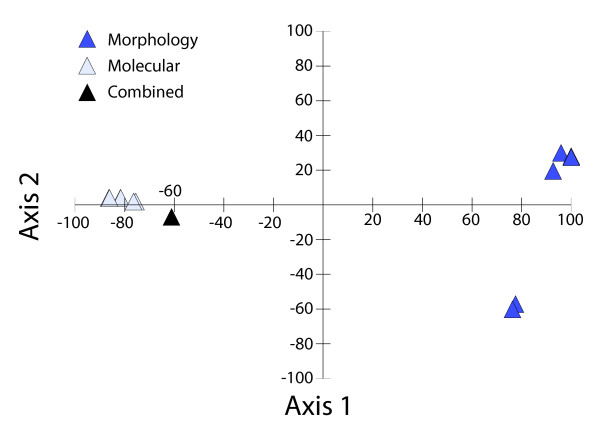
**Principal coordinate analysis of partition metrics comparing optimal molecular, morphological and combined trees**. The scatter plot shows the results from the first two axes of the principal coordinates that explain 97% of the overall variation between the scores.

Maximum likelihood analysis of the molecular data produced a single most likely tree for avian feather lice (Figure 2), and the Bayesian gene-partitioned analysis recovered a tree topology that was identical. In general, bootstrap analyses revealed relatively high support for many branches, particularly more terminal branches. Bayesian posterior probabilities were also high, with 25 of 42 (60%) branches being supported by more the 90% posterior probability (Figure 2). In many aspects the topology of this tree agrees with previous molecular based studies of lice [24-27], and differences involve only weakly supported nodes in these prior studies. Parsimony analysis of the morphological data (Additional files 1 and 2) resulted in six most parsimonious trees (Figure 3). These trees differed substantially from the molecular based tree as can be seen by principal coordinate analysis of their partition metric scores (Figure 4). Six trees resulted from combined parsimony analysis of both the molecular and morphological datasets. The consensus of these trees was more resolved than the consensus of the morphological trees, and analysis of partition metrics indicated these trees were intermediate between the molecular and morphological trees, but most closely resembling the molecular tree (Figure 4), likely because of the larger number of molecular characters and generally weaker support for the morphological tree. The SH-test [28] indicated that, using the molecular dataset, both the morphological tree and a tree in which each microhabitat specialization was constrained to be monophyletic were significantly less likely (all differences in likelihood exceeded 637 and all *P *< 0.001).

When microhabitat specialization was reconstructed over the molecular tree there were 18 changes in microhabitat specialization, which was only slightly fewer than that expected by chance according to the Maddison and Slatkin [29] test (*P *= 0.03). These randomizations suggested that over the molecular tree, microhabitat specialization is only weakly correlated with the phylogeny. In contrast, reconstructing microhabitat specialization over the six most parsimonious morphological trees required only 9 or 11 steps (depending on tree), and randomizations indicated microhabitat specialization was highly correlated with the tree produced by the morphological dataset (*P *< 0.001). These randomizations suggest that over the morphological trees, microhabitat specialization was strongly correlated with the phylogeny. Reconstructions of host group over the molecular tree required 24 steps and these were significantly fewer than expected by chance with the Maddison and Slatkin [29] test (*P *< 0.001). Reconstruction of host group over all six morphological trees resulted in more steps (31 in each case) than for over the molecular tree (24), and this larger number of steps was not fewer than expected by chance (*P *= 0.458). These results indicate that over the molecular tree, host group is strongly correlated with the phylogeny, while over the morphological tree it is not.

**Figure 5:**
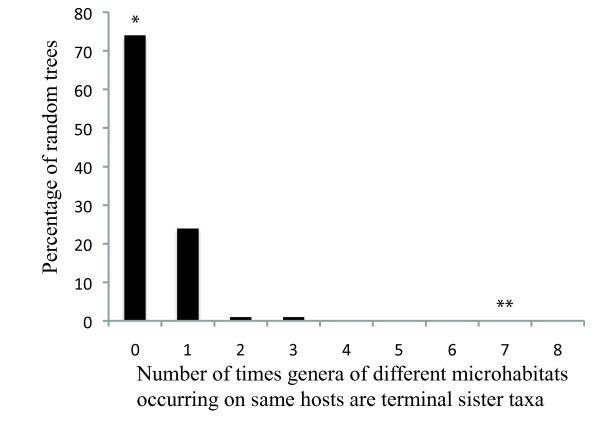
**Null distribution over random trees for sister groupings of different ecomorphs on same host group**. Cases in which lice of different microhabitat specializations, but from the same host group were terminal sister taxa were counted for each randomized tree. Actual number using the morphological (*) and molecular (**) trees indicated in relation to this null distribution.

In the molecular tree there were seven cases where species of lice of different microhabitat specializations, but from the same host group, were terminal sister taxa. Compared to a distribution based on randomized trees (Figure 5), this number was many more than would be expected by chance (*P *< 0.001). The maximum possible number for our dataset would be 14 pairs. For the morphological trees, it was never observed that lice from different microhabitat specializations, but from the same host group, were sister taxa, and this was well within the distribution of random trees (Figure 5).

## Discussion

Phylogenetic analyses based on one mitochondrial and two nuclear genes for avian feather lice revealed a pattern of repeated adaptive divergence of microhabitat specialization in these ectoparasites. Although there were some groups of genera possessing a particular microhabitat specialization, there was little indication of strong phylogenetic conservation in this trait. Rather, lice parasitizing the same group of hosts tended to diverge in their microhabitat specialization in a repeated pattern over the tree. The number of divergence events within host groups was many more than expected by chance revealing a pronounced pattern of repeated divergence of this trait *within *host groups and broad scale convergence *across *the radiation of feather lice.

Trees resulting from analysis of morphological data produced a dramatically different pattern, suggesting that most morphological characters are likely to be correlated with microhabitat specialization. This convergence likely obscures the true pattern of evolutionary relationships when inferred from morphology alone. In particular, microhabitat specialization was highly correlated with the morphological tree (*P *< 0.001). Based on the molecular tree, microhabitat specialization is highly convergent, and this convergence also extends to gross morphological features (Figure 1). Thus, selection for general morphological shape may affect a suite of morphological characters correlated with that shape.

Considering host group, the molecular tree shows much more conservation in host group usage than the morphological tree. This observation suggests that divergence in microhabitat and morphology occurs on lice within a group of hosts. It is currently unclear precisely how this divergence may be occurring. One possibility would be a form of sympatric speciation, whereby lice that live in different microhabitats on a host preferentially mate with other lice in those habitats. This would potentially reinforce character divergence associated with different selection pressures in different microhabitats. However, given that lice from different microhabitats often move to the same part of the host to feed [30], such a scenario may be unlikely. Another possibility is that this divergence is facilitated by host switching events and associated behavioral plasticity. Host transfer experiments have demonstrated that when wing lice from rock pigeons are moved to smaller dove species, these lice shift their microhabitat to the head [23]. This is a plastic behavioral response, presumably resulting from the inability of these lice to insert between the wing feather barbs on these smaller hosts [16]. If this host switching occurred in nature, such behavioral plasticity may facilitate survival of wing lice that ended up on smaller hosts. Once they shift to the head, this would in turn select on morphology, producing correlated evolution between ever stronger microhabitat preference and morphological features, facilitating survival in that microhabitat. Experiments are needed to uncover the mechanisms that might be associated with the early stages of microhabitat divergence. However, even though we uncovered a number of divergence events between closely related lice in different microhabitats, these are still relatively rare across avian feather lice, because in most cases, lice in the same genus possess the same microhabitat specialization.

Our study provides a potential example of repeated adaptive divergence in a group of parasites. In general, cases of repeated adaptive radiation or divergence are rare [5]. Most of the examples documented to date involve radiations on islands or in lakes. We are aware of one other case of repeated adaptive divergence in parasites, in non-pollinating gall-inducing fig wasp parasites [31], in which sister species of wasps parasitizing the same fig host species have diverged in their ovipositor length. This difference relates to the time during the development of the fig in which the gall-inducing parasite attacks the fig, which drives repeated morphological divergence of ovipositor length. However, given the strong morphological convergence predicted with such repeated adaptive divergence patterns, it may be that there are many more cases awaiting discovery when examined in detail with molecular based phylogenies.

These systems have several features in common which include isolation and relative simplicity of habitats. Isolation might be an important feature because if colonization opportunities are limited, only a single lineage may initially colonize an island, lake or host species. If there are multiple "niches" available, this might promote a single lineage to diversify and fill those niches. Those lineages may then subsequently co-radiate with their hosts, which may be why this process is evident mainly at the generic level. Habitat simplicity might also be an important factor. For example, for the avian feather lice, most groups are feeding on the same resources (the downy portions of the feathers [30]). There are very few cases in which congeneric feather lice of the same microhabitat specialization parasitize the same bird species [15], and in some of these cases lice partition the host range geographically because of variation in environmental conditions [32]. Competition studies have revealed that differences in escape from host defense may also mediate this resource competition [33]. Given that feather lice have only a limited number of ways in which to escape host defense, the lines along which lineages can diverge appear to be limited. With few exceptions, most species of birds host only one to three species of ischnoceran feather lice [15]. Once an escape mechanism evolves, it appears that there is strong selection on morphology for features that optimize escape ability [16]. Further work is needed on taxa in which repeated adaptive divergence occurs to more fully understand the mechanisms favoring such patterns.

## Conclusions

The radiation of avian feather lice into discrete ecomorphs has occurred along the lines of repeated adaptive divergence. This phenomenon involves both divergence of parasite morphology *within *host lineages, and convergence of parasite morphology *between *host lineages. While repeated adaptive radiations have generally been thought to be rare, being confined to island and lake systems, our study is one of the first comprehensive demonstrations of this pattern in a host-parasite system. A major difficulty of detecting such phenomena is that they are expected to exhibit morphological convergence, which would obscure evolutionary relationships reconstructed on the basis of morphology alone.

## Methods

### Molecular phylogenetics

Representatives of 39 genera of avian feather lice were obtained using ethyl acetate fumigation [34] for inclusion in a molecular phylogenetic analysis (Table 1). Special effort was made to include lice from different microhabitats, but from the same group (family or order) of birds in our study (indicated in Table 1). This sample includes about 30% of described genera [15], and phylogenetic studies to date indicate that most genera of feather lice are monophyletic [24,35,36], although there are a few notable exceptions [37]. For an outgroup, we included four genera of the ischnoceran family Trichodectidae, which are parasites of mammals (see Table 1). Total genomic DNA was extracted from single specimens of lice by first removing the head from the body and placing both in the digestion buffer from a Qiagen Tissue Extraction kit (Qiagen Sciences, Maryland, USA), incubating for 56 hours, and following the manufacturer's protocols for the remainder of the extraction. After extraction, the head and body of each louse were mounted together on a microscope slide as a voucher for identification. Voucher slides are deposited in the Illinois Natural History Survey Insect Collection and the Price Institute of Phthirapteran Research, University of Utah.

**Table 1:** Species and specimens used for molecular phylogenetic analysis

Louse Species	Host	Host group	Niche	Voucher code
*Alcedoecus alatoclypeatus*	*Halcyon malimbica*	Alcidinidae	Head	Alsp.Hamal.1.16.2001.11
*Alcedoffula duplicata*	*Ceryle rudis*	Alcidinidae	Generalist	Afdup.3.16.2001.10
*Anaticola crassicornis*	*Anas platyrhynchos*	Anseriformes	Wing	Ancra.10.17.2000.3
*Anatoecus icterodes*	*Anas discors*	Anseriformes	Head	Atsp.Andis.9.27.2000.6
*Quadraceps punctata*	*Larus californica*	Charadriiformes	Generalist	Qupun.3.24.2001.8
*Saemundssonia lari*	*Larus cirrocephalus*	Charadriiformes	Head	Salar.4.7.1999.12
*Ardeicola expallidus*	*Bubulcus ibis*	Ciconiiformes	Wing	Arexp.9.27.2000.8
*Ibidoecus bisignatus*	*Plegadis chihi*	Ciconiiformes	Head	Ibbis.9.27.2000.3
*Campanulotes compar*	*Columba livia*	Columbiformes	Body	Cabid.6.29.1998.2
*Coloceras sp*.	*Phapitreron leucotis*	Columbiformes	Body	Ccsp.Phleu.7.1.1999.5
*Columbicola columbae*	*Columba livia*	Columbiformes	Wing	Cocol.6.29.1998.1
*Cuculicola atopus*	*Piaya cayana*	Cuculiformes	Generalist	Cuato.1.27.1999.4
*Vernoniella bergi*	*Guira guira*	Cuculiformes	Generalist	Veber.10.17.2000.7
*Craspedorrhynchus hirsutus*	*Buteo regalis*	Falconiformes	Head	Cfhir.1.15.2000.6
*Degeeriella carruthi*	*Falco sparvarius*	Falconiformes	Generalist	Dgcar.9.8.1999.7
*Falcolipeurus marginalis*	*Cathartes aura*	Falconiformes	Wing	Famar.6.9.2001.4
*Chelopistes sp*.	*Ortalis canicollis*	Galliformes	Body	Chsp.Orcan.11.10.2001.9
*Goniocotes chrysocephalus*	*Phasianus colchicus*	Galliformes	Body	Gosp.Phcol.11.10.2001.2
*Oxylipeurus chiniri*	*Ortalis vetula*	Galliformes	Wing	Oxchi.1.27.1999.6
*Incidifrons transpositus*	*Fulica americana*	Gruiformes	Head	Intra.1.15.2000.9
*Meropoecus sp*.	*Merops gularis*	Meropidae	Head	Mrsp.Megul.3.24.2001.11
*Meropsiella sp*.	*Merops gularis*	Meropidae	Generalist	Brsp.Megul.3.24.2001.10
*Osculotes curta*	*Opisthocomus hoazin*	Opisthocomidae	Body	Oscur.10.5.1999.2
*Pessoaiella absita*	*Opisthocomus hoazin*	Opisthocomidae	Generalist	Wiabs.10.5.1999.2
*Brueelia ornatissima*	*Molothrus ater*	Passeriformes	Generalist	Brsp.Moate.3.24.2001.3
*Sturnidoecus sp*.	*Turdus grayi*	Passeriformes	Head	Snsp.Tugra.10.16.2002.1
*Pectinopygus bassani*	*Morus serrator*	Pelecaniformes	Wing	Pgbas.11.10.2001.13
*Picicola porisma*	*Colaptes auratus*	Piciformes	Generalist	Pipor.10.17.2000.5
*Docophoroides brevis*	*Diomedea epomophora*	Procellariiformes	Head	DOCbrev1 (from GenBank)
*Harrisoniella densa*	*Diomedea immutabilis*	Procellariiformes	Wing	HARdensa (from GenBank)
*Forficuloecus palmai*	*Barnardius zonarius*	Psittaciformes	Head	Ffpal.11.22.2001.14
*Psittaconirmus forficuloides*	*Psephotus varius*	Psittaciformes	Wing	Pcfor.10.16.2002.8
*Psittoecus eos*	*Cacatua sanguinea*	Psittaciformes	Body	Qkeos.5.16.2002.5
*Strigiphilus crucigerus*	*Otus guatemalae*	Strigiformes	Head	Stcru.1.27.1999.10
*Struthiolipeurus nandu*	*Rhea americana*	Struthioniformes	Generalist	Slnan.2.4.2002.4
*Discocorpus mexicanus*	*Crypturellus cinnamomeus*	Tinamiformes	Body	Dimex.1.27.1999.8
*Pseudolipeurus similis*	*Crypturellus cinnamomeus*	Tinamiformes	Wing	Pssim.1.27.1999.5
*Pseudophilopterus hirsutus*	*Crypturellus undulatus*	Tinamiformes	Head	Qshir.2.1.2000.11
*Strongylocotes orbicularis*	*Crypturellus parvirostris*	Tinamiformes	Body	Sgorb.11.10.2001.10
**Outgroups**				
*Bovicola bovis*	*Bos taurus*	Mammals		Bobov.2.4.2002.2
*Felicola subrostratus*	*Felis domestica*	Mammals		Fesub.2.4.2002.7
*Stachiella larseni*	*Mustela vison*	Mammals		Shlar.3.16.2001.4
*Trichodectes octomaculatus*	*Procyon lotor*	Mammals		Tdoct.2.4.2002.1

Using PCR, we amplified portions of the mitochondrial cytochrome oxidase I (COI), nuclear elongation factor-1α (EF-1α), and nuclear wingless (WNG) genes for each of the species in the study. Primers for COI were L6625 and H7005 [38], for EF-1α were EF1-For3 and Cho10 [39], and for WNG were WG1 and WG2 [40]. PCR and sequencing protocols followed Cruickshank *et al. *[24] and sequences for complementary strands were resolved and the sequences aligned by eye across species in the program Sequencher 4.7 (GeneCodes Corporation, Ann Arbor, Michigan, USA). For 15 taxa, the wingless gene could not be successfully amplified and these taxa were coded as missing data for this gene. All sequences were deposited in GenBank (accession numbers Additional file 3).

To reconstruct a molecular phylogenetic tree for lice we conducted a combined maximum likelihood search using the GTR+I+G model using fixed model parameters in PAUP* [41], which was the preferred model indicated by a MrModeltest [42] analysis. The total sequence length of the dataset was 1,119 bp. We used TBR branch swapping with 10 random addition replicates. Bootstrap resampling replicates (100 total) using the fixed model parameters were used to evaluate branch support for this tree. We also performed a Bayesian maximum likelihood analysis partitioned by gene region. MrModeltest analyses indicated GTR+I+G as the preferred model for both COI and wingless, while an AIC selection criterion preferred the HKY+I+G for EF-1α. A partitioned Bayesian search was conducted using 10 million MCMC generations with the preferred model for each of the three gene partitions and sampling every 1,000 generations. The first one million generations were discarded as burn-in. This search was conducted twice to compare the results from independent runs. From these analyses, Bayesian posterior probabilities were calculated by evaluating the frequency of branches in the distribution of trees from the MCMC run. None of the Bayesian posterior probabilities for branches in the two runs differed by more than 1%, so we present only the results of the first run, given that the two are highly consistent.

### Morphological data

We followed the procedures of Smith [43] to code additional genera of avian Ischnocera (Additional file 1) for a morphological dataset of 138 adult and nymphal characters (Additional file 2). When possible, we matched species in the molecular analysis for the morphological character coding. However, because the molecular analysis relied on fresh material for sequencing, while the morphological study relied on slide mounted series of both nymphal and adult specimens, we were not able to match species for all the genera in this study (Table 1). Instead, we used a generic exemplar approach and analyzed the data at the generic level. Because genera of feather lice appear to capture most of the diversity in the patterns of host group use and microhabitat specialization, phylogenies using a generic exemplar approach should uncover broad trends in macroevolution of diversity in this group of parasites. We also extended the coding of morphology beyond just the taxa included in the molecular analysis to nearly all recognized genera of avian Ischnocera (sensu Price *et al. *[15]). Although detailed analysis of the morphological data for all genera is beyond the scope of this study, we include the full morphological data matrix in Additional file 1. The morphological dataset was analyzed using unordered parsimony with 1,000 random addition TBR branch swapping replicates using PAUP* [41], and we conducted 1,000 bootstrap replicates.

### Data comparisons

To evaluate the magnitude of difference between the morphological and molecular datasets, we first examined the effect of combining molecular and morphological datasets. We conducted an equally weighted parsimony analysis of the combined data. We then compared the tree topologies resulting from the molecular, morphological and combined analyses using the partition metric [44] in PAUP* [41], also called symmetric difference distance or Robinson-Foulds metric. This metric measures the number of incompatible partitions between tree topologies, and higher values mean more dissimilar trees. The partition metric scores were analyzed using a principle coordinates analyses to evaluate the degree of similarity between tree topologies. The combined tree was included to provide an evaluation of the relative distance between the molecular and morphological trees compared to the combined tree.

We also evaluated whether the molecular data could reject both 1) the morphological tree and 2) a tree with each microhabitat specialist constrained to be monophyletic, but otherwise identical to the ML tree. We used the SH-test [28] for this analysis using the parameters from the maximum likelihood analyses and the most likely tree from this analysis.

### Comparative methods

For each feather louse genus in the tree, we coded it according to one of four microhabitat specializations: wing, body, head or generalist (Figure 1). These were coded based on Clay [22], another previous study that coded head lice [45], and our own observations of the regions of the body from which lice are collected. A previous study [45] that coded louse ecomorph based on morphology found 100% consistency between three independent observers coding louse ecomorph based on morphology alone, because, in general, these ecomorphs are highly divergent morphologically (see Figure 1). We then mapped these four character states over both the molecular and morphological trees using parsimony. Because our methods involved identifying the minimum number of changes in these character states, and not reconstructing ancestral states *per se*, maximum parsimony is a suitable method for this reconstruction. We quantified the minimum number of changes (steps) in these character states as well as their consistency index using MacClade [46]. We also evaluated if these characters were significantly conserved over both trees by randomizing them to taxa 1,000 times and calculating whether the observed number of steps was much less than expected by chance according to the procedure of Maddison and Slatkin [29]. We also wanted to evaluate whether host group (family or order of birds) was significantly correlated with either the morphological or molecular phylogeny. We coded lice according to the group of birds on which they occurred (from Table 1) and repeated the Maddison and Slatkin [29] test using host group as a character.

We also evaluated the number of times lice of different microhabitat specializations from the same host group were terminal sister taxa in the tree. We counted the number of times this occurred for both the molecular and morphological trees. To evaluate whether these values were greater than expected by chance, we created 100 randomized trees in MacClade and counted the number of times lice of different microhabitat specializations, but from the same host group, were terminal sister pairs. We then compared the number of cases obtained from the molecular and morphological trees with this randomized distribution to calculate a *P*-value.

## Abbreviations

AIC: Akaike information criterion; COI: cytochrome oxidase I; EF-1α: elongation factor-1α; G: gamma distribution; GTR: general time reversible; HKY: Hasegawa, Kishino, and Yano; I: invariant sites; MCMC: Markov Chain Monte Carlo; ML: maximum likelihood; PCR: polymerase chain reaction; SH-test: Shimodaira-Haswgawa test; TBR: tree-bisection-reconnection; WNG: wingless.

## Competing interests

The authors declare that they have no competing interests.

## Authors' contributions

KPJ designed the study, performed laboratory DNA sequencing, analyzed the data and drafted the manuscript. SMS performed laboratory DNA sequencing, analyzed data and helped draft the manuscript. VSS collected and analyzed the morphological data and helped draft the manuscript.

## Supplementary Material

Additional file 1 **Morphological data matrix**. This file contains the morphological data matrix for 138 adult and nymphal discrete morphological characters coded in this study. Character codings for genera not included in the phylogenetic analyses are also included for completeness. Click here for file

Additional file 2 **Morphological character definitions**. This file contains descriptions of the morphological characters and character state definitions coded in this study. Click here for file

Additional file 3 **GenBank accession numbers**. This file contains GenBank accession numbers for the sequences used in this study. Click here for file
