# Supplementary material for: Repeated adaptive divergence of microhabitat specialization in avian feather lice
Source: BMC Biol. 2012 Jun 20;10:52. doi: 10.1186/1741-7007-10-52 (PMC3391173; doi:10.1186/1741-7007-10-52)
Supplement: Additional file 1 — Morphological data matrix. This file contains the morphological data matrix for 138 adult and nymphal discrete morphological characters coded in this study. Character codings for genera not included in the phylogenetic analyses are also included for completeness. [file 1741-7007-10-52-S1.PDF]

Additional File 1. Morphological character matrix. Character descriptions as in Smith, 2001 (see Addtional File 2). Taxa marked with an asterisk are included in the morphological analysis shown in Fig. 3.

	**1**	**2**	**3**	**4**	**5**	**6**	**7**	**8**	**9**	**10**	**11**	**12**	**13**	**14**	**15**	**16**	**17**	**18**	**19**	**20**
Acidoproctus hilli	0	1	2	0	1	2	0	1	2	0	?	?	?	0	0	-	-	-	0	0
Acidoproctus rostratus	0	1	2	0	1	2	0	1	2	0	1	2	0	0	0	-	-	-	0	0
Acutifrons chimango	0	1	0	0	?	?	?	?	?	?	?	?	?	0	0	-	-	-	?	?
Aegypoecus brevicollis	2	1	2	2	1	2	2	0	-	0	?	?	?	0	1	0	0	-	1	0
Alcedoecus delphax*	3	1	4	3	0	-	0	0	-	0	0	-	0	0	1	0	1	1	0	0
Alcedoffula alcyonae*	2	1	4	3	?	?	?	1	2	0	?	?	?	0	1	0	1	0	?	1
Anaticola crassicornis*	2	1	4	1	0	-	0	0	-	0	0	-	0	0	1	0	0	-	0	0
Anatoecus icterodes*	3	1	4	3	1	0	0	1	0	0	0	-	0	0	1	0	1	1	1	1
Aquanirmus australis	2	1	4	3	1	4	2	?	?	?	1	2	0	0	1	0	1	0	2	?
Archolipeurus nandu	0	1	0	0	1	0	0	1	0	0	0	-	0	0	0	-	-	-	0	0
Ardeicola elongata	2	1	2	2	1	2	2	1	2	2	0	-	-	2	1	1	1	0	1	1
Ardeicola smithersi*	2	1	2	0	?	?	?	1	2	0	?	?	?	2	1	1	1	0	?	1
Ardeiphagus cochlearius	2	1	4	2	?	?	?	?	?	?	?	?	?	?	1	0	1	0	?	?
Auricotes affinis	0	0	-	0	0	-	0	?	?	?	?	?	?	0	0	-	-	-	0	?
Austrogoniodes waterstoni	0	0	-	0	0	-	0	0	-	0	0	-	0	0	0	-	-	-	0	0
Austrophilopterus spinosus	2	1	2	0	1	2	0	1	2	0	1	2	0	0	1	0	1	1	2	2
Bedfordiella unica	2	1	4	3	0	-	0	?	?	?	?	?	?	1	1	0	1	0	0	?
Bizarrifrons magus	2	1	4	2	?	?	?	1	4	2	?	?	?	0	1	0	0	0	0	1
Bothriometopus macrocnemis	0	1	2	0	1	2	0	1	2	0	1	2	0	0	0	-	-	-	0	0
Bovicola limbatus*	0	1	0	0	?	?	0	1	0	0	1	0	0	0	0	-	-	-	0	0
Brueelia ornatissima	1	1	2	0	1	0	0	1	0	0	0	-	0	0	0	-	-	-	0	0
Brueelia semiannulata*	1	1	0	0	1	0	0	1	0	0	0	-	0	0	0	-	-	-	0	0
Bucerocophorus pachycnemis	2	1	2	0	?	?	?	?	?	?	?	?	?	0	1	0	1	1	?	?
Buceroemersonia clarkei	0	1	3	2	?	?	?	1	3	2	?	?	?	0	0	-	-	-	?	0
***Buceronirmus* sp.**	0	0	-	0	0	-	0	0	-	0	0	-	0	0	0	-	-	-	0	0
Buceronirmus deignani	0	0	-	0	?	?	?	?	?	?	?	?	?	0	0	-	-	-	?	?

	1	2	3	4	5	6	7	8	9	10	11	12	13	14	15	16	17	18	19	20
Bucorvellus docophorus	0	1	3	2	0	-	0	?	?	?	?	?	?	0	0	-	-	-	0	?
Buerelius longiceps	2	1	4	2	1	4	2	?	?	?	?	?	?	0	0	-	-	-	0	?
Campanulotes compar*	0	0	-	0	0	-	0	0	-	0	?	?	?	0	0	-	-	-	0	0
***Capraiella* sp.**	0	0	-	0	1	3	2	1	3	2	1	3	2	0	0	-	-	-	0	0
Capraiella sabzak	0	0	-	0	?	?	?	?	?	?	?	?	?	0	0	-	-	-	?	?
Carduiceps cingulatus	3	1	4	3	1	4	3	1	2	0	?	?	?	0	1	0	0	-	2	0
Centropodiella borneoensis	1	1	0	0	?	?	?	?	?	?	?	?	?	0	0	-	-	-	?	?
Chelopistes guttatus*	0	0	-	0	0	-	0	0	-	0	0	-	0	?	0	-	-	-	0	0
Chelopistes meleagridis	0	0	-	0	0	-	0	0	-	0	?	?	?	0	0	-	-	-	0	0
Cirrophthirius testudinarius	2	1	4	3	1	0	0	1	0	0	1	0	0	0	1	0	1	0	0	0
Colilipeurus colius	1	0	-	0	0	-	0	0	-	0	0	-	0	0	0	-	-	-	0	0
Colinicola mearnsi	0	0	-	0	0	-	0	0	-	0	?	?	?	0	0	-	-	-	0	0
Coloceras damicorne*	0	0	-	0	0	-	0	0	-	0	?	?	?	0	0	-	-	-	0	0
Columbicola columbae*	0	1	3	3	1	3	3	1	3	3	0	-	2	0	0	-	-	-	0	0
Cotingacola rupicolae	0	0	-	0	0	-	0	0	-	0	0	-	0	0	0	-	-	-	0	0
Craspedonirmus colymbinus	3	1	4	3	1	4	3	1	0	0	1	0	0	0	1	0	0	-	0	0
Craspedorrhynchus platystomus*	3	1	4	3	1	2	0	1	2	0	1	2	0	0	1	0	1	1	2	2
Cuclotocephalus extraneus	0	0	-	0	?	?	?	?	?	?	?	?	?	0	0	-	-	-	?	?
Cuclotogaster madagascariensis	0	0	-	0	0	-	0	0	-	0	?	?	?	0	0	-	-	-	0	0
Cuculicola atopus*	0	1	3	0	1	3	0	1	3	0	1	3	0	0	0	-	-	-	0	0
Cuculoecus latifrons	3	1	4	2	1	4	2	1	4	2	1	4	2	0	1	0	0	-	2	2
Cummingsiella ambigua	2	1	4	2	1	4	2	1	4	2	1	1	0	0	1	0	1	1	2	2
Dahlemhornia asymmetrica	0	1	0	0	1	0	0	1	0	0	?	?	?	0	0	-	-	-	0	0
Damalinia (Damalinia) crenelata	0	1	0	0	1	0	0	1	0	0	1	0	0	0	0	-	-	-	0	0
Degeeriella rufa*	0	0	-	0	0	-	0	0	-	0	0	-	0	0	0	-	-	-	0	0
Discocorpus c. cephalosus*	0	1	2	0	0	-	0	0	-	0	?	?	?	0	0	-	-	-	0	0

	1	2	3	4	5	6	7	8	9	10	11	12	13	14	15	16	17	18	19	20
Docophoroides brevis*	2	1	4	3	1	2	2	1	2	2	0	-	0	1	1	0	1	1	2	2
Echinophilopterus protrusus	2	1	4	2	1	4	2	1	4	2	1	4	2	0	1	0	1	1	2	2
Emersoniella bracteata	1	1	1	0	1	1	0	0	-	0	0	-	0	0	0	-	-	-	0	0
Episbates pederiformis	0	0	-	0	0	-	0	0	-	0	0	-	0	0	0	-	-	-	0	0
Esthiopterum giganteum	0	1	0	0	1	0	0	1	0	0	0	-	0	0	0	-	-	-	0	0
Falcolipeurus affulgeus*	0	0	-	0	0	-	0	0	-	0	?	?	?	0	0	-	-	-	0	0
Falcolius elbeli	0	1	0	0	1	0	0	0	-	0	0	-	0	0	0	-	-	-	0	0
Felicola (F.) viverriculae*	0	1	0	0	1	0	0	1	0	0	1	0	0	0	0	-	-	-	0	0
Forficuloecus emersoni*	2	1	2	2	1	2	2	1	2	2	1	2	0	0	1	0	1	1	2	2
Formicaphagus pittasomae	2	1	4	3	1	4	3	1	4	3	1	4	3	0	1	0	1	0	2	2
Formicaricola willisi	2	1	4	2	1	2	0	1	2	0	1	0	0	0	1	0	1	0	2	2
Fulicoffula longipila	2	1	2	0	1	0	0	1	0	0	1	0	0	2	1	1	1	0	0	0
Furnariphilus pagei	2	1	2	0	1	2	0	1	3	2	1	3	2	0	1	0	1	0	2	0
Geomydoecus (G.) heaneyi	0	1	0	0	1	0	0	1	0	0	1	0	0	0	0	-	-	-	0	0
Goniocotes gallinae*	0	0	-	0	0	-	0	0	-	0	0	-	0	0	0	-	-	-	0	0
Goniodes kéleri	0	0	-	0	0	-	0	0	-	0	0	-	0	0	0	-	-	-	0	0
Goniodes pavonis	0	0	-	0	0	-	0	0	-	0	0	-	0	0	0	-	-	-	0	0
Haffneria grandis	2	1	2	0	1	1	0	1	1	0	0	-	0	1	1	0	1	0	1	1
Halipeurus pelagicus	2	1	4	2	1	1	0	1	1	0	1	1	0	1	1	0	1	0	0	0
Harrisoniella copei	2	1	4	2	1	1	0	1	1	0	0	-	0	1	1	0	1	0	0	0
Harrisoniella hopkinsi*	2	1	4	2	1	1	0	1	1	0	?	?	?	1	1	0	1	0	1	1
Heptapsogaster temporalis	0	0	-	0	0	-	0	0	-	0	?	?	?	0	0	-	-	-	0	0
Hopkinsiella clavigera	1	1	0	0	?	?	?	?	?	?	?	?	?	0	0	-	-	-	?	?
Ibidoecus plataleae*	2	1	4	2	1	4	2	1	4	2	1	4	2	0	1	2	1	1	2	2
Incidifrons fulicae*	3	1	4	3	1	2	0	1	2	0	?	?	?	0	1	0	1	1	2	2
Incidifrons transpositus	2	1	4	2	?	?	?	1	4	2	1	0	0	0	1	0	1	1	?	2

	1	2	3	4	5	6	7	8	9	10	11	12	13	14	15	16	17	18	19	20
Kelloggia coniceps	0	0	-	0	0	-	0	0	-	0	?	?	?	0	0	-	-	-	0	0
Kodocephalon latum	0	0	-	0	?	?	?	?	?	?	?	?	?	0	0	-	-	-	?	?
Labicotes guttatus	0	0	-	0	?	?	?	?	?	?	?	?	?	0	0	-	-	-	?	?
Lagopoecus affinis	0	0	-	0	0	-	0	0	-	0	0	-	0	0	0	-	-	-	0	0
Lamprocorpus hirsutus	0	0	-	0	0	-	0	0	-	0	?	?	?	0	0	-	-	-	0	0
Lipeurus caponis	0	0	-	0	0	-	0	0	-	0	0	-	0	0	0	-	-	-	0	0
Lunaceps numenii	0	1	0	2	1	0	0	1	0	0	?	?	?	0	0	-	-	-	0	0
Megaginus sordidus	0	0	-	0	0	-	0	0	-	0	?	?	?	0	0	-	-	-	0	0
Megapeostus asymmetricus	0	0	-	0	0	-	0	0	-	0	0	-	0	0	0	-	-	-	0	0
Megapodiella nakatae	0	0	-	0	?	?	?	?	?	?	?	?	?	0	0	-	-	-	?	?
Meinertzhageniella lata	0	1	0	0	?	?	?	?	?	?	?	?	?	0	0	-	-	-	?	?
Meropoecus meropis*	3	1	2	1	1	2	1	1	2	1	1	4	2	0	1	0	1	0	2	2
Meropsiella* sp.	1	1	2	0	?	?	?	?	?	?	?	?	?	0	0	-	-	-	?	?
***Mulcticola* sp.**	2	1	4	2	1	2	0	1	2	0	0	-	0	0	1	0	1	0	2	2
Naubates fuliginosus	2	1	4	3	1	1	0	1	1	0	1	1	0	1	1	0	1	0	1	0
Neophilopterus heteropygus	2	1	4	2	1	2	2	1	0	0	1	0	0	0	0	-	-	-	0	0
Neopsittaconirmus borgiolii	0	1	1	0	0	-	0	0	-	0	0	-	0	0	0	-	-	-	0	0
Nesiotinus demersus	0	1	0	0	?	?	?	?	?	?	?	?	?	0	0	-	-	-	?	?
Nothocotus subsimilis	0	0	-	0	?	?	?	?	?	?	?	?	?	0	0	-	-	-	?	?
Nyctibicola longirostris	2	1	4	3	?	?	?	?	?	?	?	?	?	0	1	0	1	0	?	?
Ornicholax robustus	0	0	-	0	0	-	0	?	?	?	?	?	?	0	0	-	-	-	0	?
Ornithobius goniopleurus	0	1	2	0	1	2	0	1	2	0	0	-	0	0	0	-	-	-	0	0
Osculotes curtus	0	1	2	0	1	0	0	1	0	0	?	?	?	0	0	-	-	-	0	0
Osculotes macropoda*	0	1	2	0	1	0	0	1	0	0	?	?	?	0	0	-	-	-	0	0
***Otidoecus* sp.**	0	0	-	0	0	-	0	0	-	0	0	-	0	0	0	-	-	-	0	0
Oxylipeurus dentatus*	0	0	-	0	0	-	0	0	-	0	0	-	0	0	0	-	-	-	0	0

	1	2	3	4	5	6	7	8	9	10	11	12	13	14	15	16	17	18	19	20
Pachyskelotes orthopleurus	0	0	-	0	0	-	0	0	-	0	?	?	?	0	0	-	-	-	0	0
Paraclisis diomedea	2	1	4	2	1	1	0	1	1	0	0	-	0	1	1	0	1	0	1	0
Paragoniocotes rotundus	0	0	-	0	0	-	0	0	-	0	?	?	?	0	0	-	-	-	0	0
Paragoniocotes venezolanus	0	0	-	0	0	-	0	0	-	0	0	-	0	0	0	-	-	-	0	0
Paroncophorus javanicus	2	0	-	0	?	?	?	0	-	0	?	?	?	0	0	-	-	-	?	0
Passonomedea hopkinsi	0	0	-	0	0	-	0	0	-	0	0	-	0	0	0	-	-	-	0	0
Pectenosoma verrucosa	0	0	-	0	0	-	0	0	-	0	0	-	0	0	0	-	-	-	0	0
*Pectinopygus bassani**	2	1	4	2	1	1	0	1	1	0	1	1	0	1	1	0	1	0	1	1
Pectinopygus sulae	2	1	4	2	?	?	?	1	1	0	1	1	0	1	1	0	1	0	?	1
Pelmatocerandra setosa	2	1	4	2	1	1	0	1	1	0	1	3	3	1	1	0	1	0	0	0
Penenirmus auritus	2	1	4	2	1	4	2	1	4	2	1	4	2	0	1	0	1	0	2	2
Perineus nigrolimbatus	2	0	-	2	0	-	0	0	-	0	0	-	0	1	0	-	-	-	0	0
*Pessoaiella absita**	2	1	4	2	1	2	0	1	2	0	0	-	0	0	1	0	1	0	1	1
Philoceanus garrodiae	2	1	2	0	1	1	0	1	1	0	?	?	?	1	1	0	1	0	?	?
Philopterus ornatus	2	1	4	3	1	4	3	1	4	3	?	?	?	0	1	0	0	-	2	2
Physconella kelloggi	0	0	-	0	?	?	?	?	?	?	?	?	?	0	0	-	-	-	?	?
Physconelloides cubanus	0	0	-	0	0	-	0	0	-	0	0	-	0	0	0	-	-	-	0	0
*Picicola snodgrassi**	0	0	-	0	0	-	0	?	?	?	0	-	0	0	0	-	-	-	0	?
Podargoecus strigoides	3	1	4	3	1	2	0	0	-	0	0	-	0	0	1	0	1	0	1	0
Pseudocophorus perijanus	0	1	4	2	?	?	?	?	?	?	?	?	?	0	0	-	-	-	?	?
*Pseudolipeurus similis**	2	1	4	2	?	?	?	?	?	?	?	?	?	0	1	0	0	-	?	?
Pseudonirmus gurlti	2	1	4	2	1	1	0	1	1	0	0	-	0	0	1	0	1	0	0	0
*Pseudophilopterus hirsutus**	2	1	2	2	?	?	?	?	?	?	?	?	?	0	1	0	0	-	?	?
*Psittaconirmus (P.) zinki**	0	1	2	2	?	?	?	?	?	?	?	?	?	0	0	-	-	-	?	?
*Psittoecus vanzolinii**	0	1	2	0	1	2	0	1	2	0	?	?	?	0	0	-	-	-	0	0
Pterocotes aberrans	0	0	-	0	0	-	0	0	-	0	?	?	?	0	0	-	-	-	0	0

	1	2	3	4	5	6	7	8	9	10	11	12	13	14	15	16	17	18	19	20
Quadraceps coenocoryphae*	2	1	4	3	1	2	0	1	2	0	0	-	0	0	1	0	1	1	2	2
Rallicola lugens	2	1	2	0	1	1	0	1	1	0	0	-	0	0	1	0	1	0	2	2
Rhopaloceras rudimentarius	0	0	-	0	0	-	0	0	-	0	0	-	0	0	0	-	-	-	0	0
Rhynonirmus scolopacis	0	0	-	0	0	-	0	0	-	0	0	-	0	0	0	-	-	-	0	0
Rotundiceps cordatus	0	0	-	0	0	-	0	?	?	?	?	?	?	0	0	-	-	-	0	?
Saemundssonia desolata*	2	1	4	3	1	2	2	1	2	2	1	2	0	0	1	0	1	1	2	2
Saemundssonia haematopi	2	1	4	3	1	2	0	?	?	?	?	?	?	0	1	0	1	1	2	?
Splendoroffula ruwenzorornis	0	0	-	0	?	?	?	0	-	0	0	-	0	0	0	-	-	-	?	0
Strigiphilus vapidus*	2	1	4	3	1	2	0	1	2	0	0	-	0	0	1	0	1	1	2	2
Strongylocotes angulocapitis*	0	0	-	0	?	?	?	0	-	0	0	-	0	0	0	-	-	-	?	0
Strongylocotes complanatus	0	0	-	0	0	-	0	0	-	0	0	-	0	0	0	-	-	-	0	0
Struthiolipeurus struthionis*	0	1	0	0	0	-	0	0	-	0	0	-	0	0	0	-	-	-	0	0
Sturnidoecus sturni*	2	1	4	2	1	4	2	1	4	2	1	2	0	0	1	0	0	-	2	2
Syrrhaptoecus falcatus	0	0	-	0	0	-	0	0	-	0	?	?	?	0	0	-	-	-	0	0
Theresiella gemina	3	1	4	3	1	4	3	1	4	3	1	4	0	0	1	0	1	?	2	2
***Tinamotaecola* sp.**	0	0	-	0	?	?	?	?	?	?	?	?	?	0	0	-	-	-	?	?
Trabeculus schillingi	2	1	4	3	1	4	3	1	4	3	1	4	2	0	1	0	1	0	2	2
Trichodectes (T.) melis*	0	1	0	0	1	0	0	1	0	0	?	?	?	0	0	-	-	-	0	0
***Trichodopeostus* sp.**	0	0	-	0	0	-	0	0	-	0	0	-	0	0	0	-	-	-	0	0
Trichophilopterus babakotophilus	0	0	-	0	0	-	0	0	-	0	0	-	0	0	0	-	-	-	0	0
Trogoniella aequatoriale	0	0	-	0	?	?	?	?	?	?	?	?	?	0	0	-	-	-	?	?
Trogoninirmus strigilatus	0	0	-	0	?	?	?	?	?	?	?	?	?	0	0	-	-	-	?	?
Turnicola angustissimus	2	1	4	3	1	4	2	?	?	?	?	?	?	0	1	0	1	0	2	?
Turturicola salimalii	0	1	3	3	?	?	?	?	?	?	?	?	?	0	0	-	-	-	?	?
Upupicola upupae	0	0	-	2	0	-	2	0	-	2	0	-	0	0	0	-	-	-	0	0
Vernoniella guimaraesi*	3	1	2	0	1	2	0	1	2	0	1	1	0	0	1	0	1	0	2	2

	21	22	23	24	25	26	27	28	29	30	31	32	33	34	35	36	37	38	39	40
Acidoproctus hilli	?	?	0	0	0	?	0	0	-	4	4	4	?	1	0	0	-	0	0	?
Acidoproctus rostratus	0	-	0	0	0	0	0	0	-	4	4	4	4	1	0	0	-	0	0	1
Acutifrons chimango	?	?	0	?	?	?	0	1	-	1	-	?	?	0	1	1	0	0	1	1
Aegypoecus brevicollis	?	?	0	0	0	?	3	?	?	3	3	2	?	1	1	1	?	0	1	0
Alcedoecus delphax*	0	-	1	0	0	0	3	2	4	3	2	2	2	1	1	1	1	0	0	1
Alcedoffula alcyonae*	?	?	1	?	1	?	3	2	4	3	?	2	?	1	1	1	1	0	0	1
Anaticola crassicornis*	0	-	0	0	0	0	3	2	4	1	1	1	1	0	1	1	0	0	0	1
Anatoecus icterodes*	0	-	1	1	1	0	3	2	4	3	3	3	3	1	1	1	1	0	1	1
Aquanirmus australis	2	0	1	1	?	1	3	2	4	3	3	?	3	1	1	1	0	0	0	1
Archolipeurus nandu	0	-	0	0	0	0	0	0	-	3	3	3	1	1	1	1	0	0	0	0
Ardeicola elongata	0	-	0	0	0	0	3	2	2	1	2	2	2	0	0	1	0	0	0	0
Ardeicola smithersi*	?	?	1	?	0	?	3	2	4	3	?	3	?	1	0	0	-	0	0	0
Ardeiphagus cochlearius	?	?	?	?	?	?	3	2	4	2	?	?	?	1	?	1	0	0	1	0
Auricotes affinis	?	?	0	0	?	?	0	0	-	0	0	?	?	0	1	1	0	0	0	1
Austrogoniodes waterstoni	0	-	0	0	0	0	0	0	-	0	0	0	0	0	0	1	0	0	0	0
Austrophilopterus spinosus	1	1	0	0	0	0	3	2	4	1	1	1	1	0	1	1	1	0	1	1
Bedfordiella unica	?	?	0	0	?	?	3	2	2	3	1	?	?	0	0	0	-	0	0	?
Bizarrifrons magus	?	?	0	?	1	?	3	2	4	3	?	3	?	1	1	1	0	0	1	0
Bothriometopus macrocnemis	0	-	0	0	0	0	0	0	-	4	4	4	4	1	0	0	-	0	0	1
Bovicola limbatus*	0	-	0	0	0	0	0	0	-	3	3	3	2	0	1	1	0	0	0	2
Brueelia ornatissima	0	-	0	0	0	0	0	0	-	2	2	2	2	1	0	0	-	0	0	0
Brueelia semiannulata*	0	-	0	0	0	0	0	0	-	2	2	2	2	1	1	1	0	0	0	0
Bucerocophorus pachycnemis	?	?	0	?	?	?	3	2	4	5	?	?	?	0	1	1	0	0	1	1
Buceroemersonia clarkei	?	?	0	?	0	?	0	1	-	1	?	1	?	0	1	1	1	0	1	1
***Buceronirmus* sp.**	0	-	0	0	0	0	2	1	-	1	1	1	1	0	1	1	1	0	0	1
Buceronirmus deignani	?	?	0	?	?	?	2	1	-	1	?	?	?	0	1	1	1	0	0	1

	21	22	23	24	25	26	27	28	29	30	31	32	33	34	35	36	37	38	39	40
Bucorvellus docophorus	?	?	0	0	?	?	0	1	-	3	3	?	?	1	1	1	0	0	1	0
Buerelius longiceps	?	?	1	1	?	?	3	2	1	3	3	?	?	1	1	1	1	0	1	0
Campanulotes compar*	?	?	0	0	0	?	0	0	-	0	0	0	?	0	0	0	-	0	0	1
Capraiella **sp.**	0	-	0	0	0	0	0	0	-	0	1	1	1	0	1	1	0	0	1	1
Capraiella sabzak	?	?	0	?	?	?	0	0	-	0	?	?	?	0	1	1	0	0	1	1
Carduiceps cingulatus	?	?	1	1	0	?	3	2	4	3	3	3	?	0	1	1	0	0	1	0
Centropodiella borneoensis	?	?	0	?	?	?	2	1	-	?	?	?	?	0	1	?	?	0	1	1
Chelopistes guttatus*	0	-	?	0	0	0	0	0	-	0	0	0	0	0	?	1	0	0	0	1
Chelopistes meleagridis	?	?	0	0	0	?	0	0	-	0	0	0	?	0	0	1	0	0	0	1
Cirrophthirius testudinarius	0	-	1	0	0	0	3	2	4	3	3	3	3	0	0	0	-	0	1	1
Colilipeurus colius	0	-	0	0	0	0	2	1	-	2	2	2	0	0	1	1	0	0	0	1
Colinicola mearnsi	?	-	0	0	0	?	0	0	-	0	0	0	?	0	1	1	0	0	0	1
Coloceras damicorne*	?	?	0	0	0	?	0	0	-	0	0	0	?	0	0	1	0	0	0	1
Columbicola columbae*	0	-	0	0	0	0	1	1	-	0	0	0	0	0	1	1	0	0	1	1
Cotingacola rupicolae	0	-	0	0	0	0	2	1	-	1	1	1	1	0	1	1	0	0	1	1
Craspedonirmus colymbinus	0	-	0	0	0	0	3	2	4	3	3	3	3	1	1	1	1	0	1	1
Craspedorrhynchus platystomus*	2	0	1	1	1	1	3	2	4	3	2	2	2	1	1	1	1	0	0	0
Cuclotocephalus extraneus	?	?	0	?	?	?	0	0	-	0	?	?	?	0	0	0	-	0	0	1
Cuclotogaster madagascariensis	?	?	0	0	0	?	0	0	-	0	0	0	?	0	1	1	0	0	0	1
Cuculicola atopus*	0	-	0	0	0	0	2	1	-	1	1	1	1	0	1	1	0	0	0	1
Cuculoecus latifrons	2	1	1	1	1	1	3	2	4	3	3	3	3	0	1	1	1	0	1	0
Cummingsiella ambigua	0	-	1	1	1	0	3	2	4	3	3	3	3	0	1	1	0	0	1	1
Dahlemhornia asymmetrica	?	-	0	0	0	?	0	0	-	3	3	3	?	?	0	0	-	0	0	0
Damalinia (Damalinia) crenelata	0	-	0	0	0	0	0	0	-	3	3	3	3	0	1	1	0	0	0	2
Degeeriella rufa*	0	-	0	0	0	0	0	0	-	1	1	1	1	0	1	1	0	0	0	1
Discocorpus c. cephalosus*	?	?	0	0	0	?	0	0	-	0	0	0	?	0	1	1	0	0	0	1

	21	22	23	24	25	26	27	28	29	30	31	32	33	34	35	36	37	38	39	40
Docophoroides brevis*	0	-	1	1	1	0	3	2	4	3	3	3	3	1	1	1	1	0	1	1
Echinophilopterus protrusus	2	2	1	1	1	1	3	2	4	3	3	3	3	0	1	1	1	0	1	0
Emersoniella bracteata	0	-	0	0	0	0	0	0	-	1	1	1	1	0	0	0	-	0	0	1
Episbates pederiformis	0	-	0	0	0	0	0	0	-	0	0	0	0	0	0	0	-	0	0	1
Esthiopterum giganteum	0	-	0	0	0	0	0	0	-	3	3	3	3	1	0	0	-	0	0	1
Falcolipeurus affulgeus*	?	?	0	0	0	?	0	0	-	2	2	2	?	1	0	0	-	0	0	0
Falcolius elbeli	0	-	0	0	0	0	0	0	-	1	1	1	1	0	0	0	-	0	0	1
Felicola (F.) viverriculae*	0	-	0	0	0	0	0	0	-	3	3	3	3	1	1	1	0	0	0	2
Forficuloecus emersoni*	1	0	1	1	1	0	3	2	4	2	2	2	2	0	1	1	0	0	1	0
Formicaphagus pittasomae	2	0	1	1	1	1	3	2	4	3	3	3	2	1	1	1	0	0	0	0
Formicaricola willisi	0	-	1	1	1	0	3	2	4	3	3	3	3	1	1	1	0	0	0	1
Fulicoffula longipila	0	-	1	0	0	0	3	2	4	2	2	2	2	1	0	0	-	0	0	0
Furnariphilus pagei	0	-	1	1	0	0	3	2	4	2	2	2	2	1	0	0	-	0	0	1
Geomydoecus (G.) heaneyi	0	-	0	0	0	0	0	0	-	3	3	3	3	1	1	1	0	0	0	2
Goniocotes gallinae*	0	-	0	0	0	0	0	0	-	0	0	0	0	0	1	1	0	0	0	1
Goniodes kéleri	0	-	0	0	0	0	0	0	-	0	0	0	0	0	0	1	0	0	0	1
Goniodes pavonis	0	-	0	0	0	0	0	0	-	0	0	0	0	0	1	1	0	0	0	1
Haffneria grandis	0	-	0	0	0	0	2	2	2	1	1	1	1	0	0	1	0	0	0	0
Halipeurus pelagicus	0	-	0	0	0	0	3	2	2	1	1	1	1	0	1	1	0	0	0	0
Harrisoniella copei	0	-	0	0	0	0	3	2	2	1	1	1	1	0	0	0	-	0	0	0
Harrisoniella hopkinsi*	1	-	0	0	0	?	3	2	2	1	1	1	?	1	0	0	-	0	0	0
Heptapsogaster temporalis	?	?	0	0	0	?	0	0	-	1	1	1	?	0	0	0	-	0	0	1
Hopkinsiella clavigera	?	?	0	?	?	?	0	1	-	1	?	?	?	0	0	1	0	0	0	1
Ibidoecus plataleae*	2	0	0	0	0	0	3	2	2	3	3	3	3	1	1	1	0	0	0	0
Incidifrons fulicae*	?	?	1	1	1	?	3	2	4	3	3	3	?	1	1	1	1	0	0	0
Incidifrons transpositus	0	-	1	?	1	1	3	2	4	3	?	3	3	1	1	1	1	0	1	1

	21	22	23	24	25	26	27	28	29	30	31	32	33	34	35	36	37	38	39	40
Kelloggia coniceps	?	?	0	0	0	?	0	0	-	0	0	0	0	0	0	0	-	0	0	1
Kodocephalon latum	?	?	0	?	?	?	0	0	-	0	?	?	?	0	1	1	0	0	0	1
Labicotes guttatus	?	?	0	?	?	?	0	0	-	0	?	?	?	0	0	0	-	0	0	1
Lagopoecus affinis	0	-	0	0	0	0	0	1	-	1	1	1	1	0	1	1	0	0	0	1
Lamprocorpus hirsutus	?	?	0	0	0	?	0	0	-	0	0	0	?	0	0	0	-	0	0	0
Lipeurus caponis	0	-	0	0	0	0	0	0	-	0	0	0	0	0	1	1	0	0	0	1
Lunaceps numenii	0	-	1	1	1	?	2	1	-	3	3	3	?	0	0	0	-	0	0	1
Megaginus sordidus	?	?	0	0	0	?	0	0	-	0	0	0	?	0	1	1	0	0	0	0
Megapeostus asymmetricus	0	-	0	0	0	0	0	0	-	1	1	1	1	0	0	0	-	0	0	1
Megapodiella nakatae	?	?	0	?	?	?	2	1	-	5	?	?	?	0	1	1	0	0	0	2
Meinertzhageniella lata	?	?	0	?	?	?	0	0	-	3	?	?	?	1	1	0	-	0	0	0
Meropoecus meropis*	2	0	1	1	1	1	3	2	4	3	3	3	3	1	1	1	0	?	0	0
Meropsiella* sp.	?	?	0	?	?	?	0	0	-	3	?	?	?	1	1	1	0	0	0	0
***Mulcticola* sp.**	0	-	1	1	1	0	3	2	4	3	3	3	2	?	1	1	0	0	0	1
Naubates fuliginosus	0	-	1	0	0	0	3	2	2	4	3	3	1	0	1	1	0	0	0	0
Neophilopterus heteropygus	0	-	0	0	0	0	3	?	4	3	3	3	3	1	1	1	0	0	0	0
Neopsittaconirmus borgiolii	0	-	0	0	0	0	0	0	-	2	2	2	2	1	1	0	-	0	0	1
Nesiotinus demersus	?	?	0	?	?	?	0	0	-	3	?	?	?	1	0	0	-	0	0	0
Nothocotus subsimilis	?	?	0	?	?	?	0	0	-	1	?	?	?	0	0	0	-	0	0	1
Nyctibicola longirostris	?	?	1	?	?	?	3	2	4	3	?	?	?	1	1	?	?	0	0	1
Ornicholax robustus	?	?	0	0	?	?	0	0	-	0	0	?	?	0	1	1	0	0	0	1
Ornithobius goniopleurus	0	-	0	0	0	0	0	0	-	4	4	4	3	1	0	0	-	0	0	0
Osculotes curtus	?	?	0	0	0	?	0	1	-	3	3	2	?	?	1	1	0	0	0	?
Osculotes macropoda*	?	?	0	0	0	?	0	1	-	3	3	3	?	?	1	1	0	0	0	?
***Otidoecus* sp.**	0	-	0	0	0	0	0	0	-	1	1	1	0	0	0	0	-	0	0	1
Oxylipeurus dentatus*	0	-	0	0	0	0	0	1	-	0	0	0	0	0	1	1	0	0	0	1

	21	22	23	24	25	26	27	28	29	30	31	32	33	34	35	36	37	38	39	40
Pachyskelotes orthopleurus	?	?	0	0	0	?	0	0	-	0	0	0	?	0	0	0	-	0	0	1
Paraclisis diomedea	0	-	1	0	0	0	3	2	2	1	1	1	1	0	0	1	-	0	0	1
Paragoniocotes rotundus	?	?	0	0	0	?	0	0	-	1	1	1	?	0	1	1	0	0	0	1
Paragoniocotes venezolanus	0	-	0	0	0	0	0	0	-	1	1	1	1	0	1	1	0	0	0	1
Paroncophorus javanicus	?	?	0	?	0	?	2	1	-	5	?	1	?	0	1	1	0	0	0	0
Passonomedea hopkinsi	0	-	0	0	0	0	0	0	-	0	0	0	0	0	0	0	-	0	0	1
Pectenosoma verrucosa	0	-	0	0	0	0	0	0	-	1	1	1	1	0	0	0	-	0	0	1
Pectinopygus bassani*	1	-	1	0	0	0	3	2	2	3	3	3	3	1	1	1	0	0	0	0
Pectinopygus sulae	1	-	1	?	0	0	3	2	2	3	?	3	3	1	1	1	0	0	0	0
Pelmatocerandra setosa	0	-	0	0	0	0	3	2	2	3	2	2	2	0	1	1	0	0	0	0
Penenirmus auritus	2	0	1	1	1	1	3	2	4	3	3	3	3	?	1	1	0	0	0	1
Perineus nigrolimbatus	0	-	0	0	0	0	2	2	2	1	1	1	1	0	0	0	-	0	0	0
Pessoaiella absita*	0	-	0	1	1	0	3	2	4	3	3	3	3	1	1	1	0	0	0	0
Philoceanus garrodiae	?	?	0	0	0	?	3	2	3	3	3	3	?	0	0	1	0	0	0	0
Philopterus ornatus	?	?	1	1	1	?	3	2	4	3	3	3	?	1	1	1	0	1	0	1
Physconella kelloggi	?	?	0	?	?	?	0	0	-	0	?	?	?	0	1	1	1	0	0	1
Physconelloides cubanus	0	-	0	0	0	0	0	0	-	0	0	0	0	0	1	1	0	0	0	1
Picicola snodgrassi*	0	-	0	0	?	0	0	0	-	1	1	?	1	0	1	1	0	0	0	0
Podargoecus strigoides	0	-	1	0	0	0	3	2	4	3	2	2	2	1	1	1	1	0	0	0
Pseudocophorus perijanus	?	?	1	?	?	?	2	1	-	3	?	?	?	1	1	1	0	0	0	0
Pseudolipeurus similis*	?	?	1	?	?	?	3	2	4	3	?	?	?	0	1	1	1	0	0	1
Pseudonirmus gurlti	0	-	0	0	0	0	3	2	1	3	3	3	3	0	0	0	-	0	0	0
Pseudophilopterus hirsutus*	?	?	1	?	?	?	3	2	4	2	?	?	?	1	1	1	0	0	1	0
Psittaconirmus (P.) zinki*	?	?	0	?	?	?	0	0	-	3	?	?	?	0	1	1	0	0	0	0
Psittoecus vanzolinii*	?	?	0	0	0	?	0	0	-	3	2	2	?	0	1	1	0	0	0	0
Pterocotes aberrans	?	?	0	0	0	-	0	0	-	1	1	1	?	0	1	1	0	0	0	1

	21	22	23	24	25	26	27	28	29	30	31	32	33	34	35	36	37	38	39	40
Quadraceps coenocoryphae*	0	-	1	0	0	0	3	2	4	3	3	3	3	1	1	1	1	0	1	1
Rallicola lugens	0	-	0	0	0	0	0	0	-	3	3	2	2	1	1	1	0	0	0	1
Rhopaloceras rudimentarius	0	-	0	0	0	0	0	0	-	0	0	0	0	0	0	1	0	0	0	1
Rhynonirmus scolopacis	0	-	0	0	0	0	2	1	-	0	0	0	0	0	1	1	0	0	0	1
Rotundiceps cordatus	?	?	0	0	?	?	2	1	-	3	3	?	?	0	0	0	-	0	0	1
Saemundssonia desolata*	2	2	1	1	1	1	3	2	4	3	3	3	3	1	1	1	1	0	1	1
Saemundssonia haematopi	?	?	1	1	?	?	3	2	4	3	3	?	?	1	1	1	1	0	1	1
Splendoroffula ruwenzorornis	0	-	0	?	0	0	0	1	-	0	?	0	0	0	0	0	-	0	0	1
Strigiphilus vapidus*	0	-	1	1	1	0	3	2	4	3	2	2	2	1	1	1	1	0	0	0
Strongylocotes angulocapitis*	0	-	0	?	0	0	0	0	-	0	?	0	0	0	1	1	0	0	0	1
Strongylocotes complanatus	0	-	0	0	0	0	0	0	-	0	0	0	0	0	1	1	0	0	0	1
Struthiolipeurus struthionis*	0	-	0	0	0	0	0	0	-	3	3	3	1	1	1	1	0	0	0	0
Sturnidoecus sturni*	1	-	1	1	1	0	3	2	4	3	3	2	2	1	1	1	1	0	0	0
Syrrhaptoecus falcatus	?	?	0	0	0	?	0	0	-	0	1	1	?	0	1	1	0	0	0	1
Theresiella gemina	2	0	1	1	1	1	3	2	4	3	3	3	3	0	1	1	0	0	0	0
***Tinamotaecola* sp.**	?	?	0	?	?	?	2	?	1	?	?	?	?	0	1	1	0	0	1	1
Trabeculus schillingi	2	0	0	0	0	0	3	2	4	5	3	3	3	0	1	1	1	0	1	1
Trichodectes (T.) melis*	?	?	0	0	0	?	0	0	-	3	3	3	?	1	0	1	0	0	0	2
***Trichodopeostus* sp.**	0	-	0	0	0	0	0	0	-	0	0	0	0	0	1	1	0	0	0	1
Trichophilopterus babakotophilus	0	-	0	0	0	0	0	0	-	2	1	1	1	0	1	1	0	0	0	2
Trogoniella aequatoriale	?	?	0	?	?	?	0	0	-	1	?	?	?	0	0	0	-	0	0	1
Trogoninirmus strigilatus	?	?	0	?	?	?	2	1	-	1	?	?	?	0	1	1	1	0	0	1
Turnicola angustissimus	?	?	0	0	?	?	3	2	1	3	3	?	?	0	1	1	0	0	1	1
Turturicola salimalii	?	?	0	?	?	?	1	1	-	0	?	?	?	0	0	0	-	0	1	1
Upupicola upupae	0	-	0	0	0	0	0	1	-	1	1	1	1	0	1	1	0	0	0	1
Vernoniella guimaraesi*	2	0	0	0	0	0	2	2	1	3	3	2	2	0	1	1	1	0	0	0

	41	42	43	44	45	46	47	48	49	50	51	52	53	54	55	56	57	58	59	60
Acidoproctus hilli	1	0	?	1	1	2	0	0	0	0	1	0	0	1	2	1	0	0	1	0
Acidoproctus rostratus	1	0	1	1	0	0	-	0	0	0	-	-	-	1	2	1	0	1	1	0
Acutifrons chimango	1	1	?	?	0	?	-	0	0	0	-	-	-	1	2	1	2	0	0	1
Aegypoecus brevicollis	0	0	0	0	0	?	-	0	0	0	-	-	-	1	2	1	2	0	1	1
*Alcedoecus delphax**	1	0	1	1	0	0	-	0	0	0	-	-	-	1	2	1	2	0	0	0
*Alcedoffula alcyonae**	1	1	?	?	0	?	-	0	0	0	-	-	-	1	0	-	-	0	1	1
*Anaticola crassicornis**	1	1	1	1	1	2	0	0	0	0	1	0	0	1	2	1	0	0	0	0
*Anatoecus icterodes**	1	1	1	1	0	?	-	0	0	0	-	-	-	1	2	1	0	0	1	0
Aquanirmus australis	1	1	1	1	0	2	-	0	0	0	-	-	-	1	2	1	0	0	1	0
Archolipeurus nandu	1	0	0	1	1	2	0	0	0	0	1	1	0	1	2	1	0	0	0	1
Ardeicola elongata	1	1	0	1	1	?	0	0	0	0	0	0	0	1	2	1	0	0	0	0
*Ardeicola smithersi**	0	1	?	?	1	2	0	0	0	0	1	0	0	1	2	1	0	0	0	0
Ardeiphagus cochlearius	1	0	?	?	?	?	?	?	?	?	?	?	?	1	2	1	0	0	0	0
Auricotes affinis	1	0	1	1	0	?	-	0	0	0	-	-	-	0	-	-	-	1	1	0
Austrogoniodes waterstoni	1	1	0	1	1	2	0	0	0	0	0	0	1	1	1	0	-	0	0	0
Austrophilopterus spinosus	1	1	1	1	0	?	-	0	0	0	-	-	-	1	2	1	2	0	0	0
Bedfordiella unica	1	1	1	1	1	?	0	0	0	0	1	1	0	1	2	0	-	0	0	0
Bizarrifrons magus	1	0	?	?	0	?	-	0	0	0	-	-	-	1	2	1	2	0	0	0
Bothriometopus macrocnemis	1	0	1	1	1	?	1	0	0	0	1	1	0	1	2	0	-	0	1	1
*Bovicola limbatus**	1	0	2	1	0	0	-	0	1	1	-	-	-	1	0	-	-	0	0	0
Brueelia ornatissima	1	0	0	1	0	?	0	0	0	0	-	-	-	1	2	1	?	0	0	0
*Brueelia semiannulata**	1	0	0	1	0	0	-	0	0	0	-	-	-	1	2	1	2	0	0	0
Bucerocophorus pachycnemis	1	1	?	?	1	?	0	0	0	0	0	0	0	0	-	-	-	0	0	1
Buceroemersonia clarkei	1	?	?	?	0	?	-	0	0	0	-	-	-	1	2	0	-	0	0	1
Buceronirmus sp.	1	1	1	1	0	?	-	0	0	0	-	-	-	1	2	1	0	0	0	0
Buceronirmus deignani	1	1	?	?	0	?	-	0	0	0	-	-	-	1	2	1	0	0	0	0

	41	42	43	44	45	46	47	48	49	50	51	52	53	54	55	56	57	58	59	60
Bucorvellus docophorus	1	0	0	1	1	?	0	0	0	0	-	-	-	1	2	1	2	0	0	1
Buerelius longiceps	1	0	0	1	0	?	-	0	0	0	-	-	-	1	2	1	0	0	0	0
Campanulotes compar*	1	0	1	1	0	0	-	0	0	0	-	-	-	0	-	-	-	1	0	0
***Capraiella* sp.**	1	1	1	1	0	?	-	0	0	0	-	-	-	1	2	1	0	0	0	1
Capraiella sabzak	1	1	?	?	0	?	-	0	0	0	-	-	-	1	2	1	0	0	0	1
Carduiceps cingulatus	1	1	1	1	0	?	-	0	0	0	-	-	-	1	2	1	0	0	0	0
Centropodiella borneoensis	1	0	?	?	0	?	-	0	0	0	-	-	-	1	2	1	1	0	0	?
Chelopistes guttatus*	1	0	1	1	?	?	?	?	0	?	?	?	?	0	-	-	-	1	1	1
Chelopistes meleagridis	1	0	1	1	1	?	1	0	0	0	1	1	0	0	-	-	-	1	1	1
Cirrophthirius testudinarius	1	1	1	1	0	?	0	0	0	0	-	-	-	1	2	?	?	0	1	1
Colilipeurus colius	1	0	1	1	1	?	0	0	0	0	0	1	0	1	2	0	-	0	0	1
Colinicola mearnsi	1	1	1	1	1	?	0	0	0	0	1	1	0	1	2	1	0	0	0	1
Coloceras damicorne*	1	0	1	1	1	0	0	0	0	0	1	0	1	0	-	-	-	1	0	0
Columbicola columbae*	0	1	1	1	1	0	0	0	0	0	1	0	0	1	0	-	-	0	0	0
Cotingacola rupicolae	1	1	1	1	0	?	-	0	0	0	-	-	-	1	2	1	0	0	0	0
Craspedonirmus colymbinus	1	1	1	1	0	0	-	0	0	0	-	-	-	1	2	0	-	0	1	0
Craspedorrhynchus platystomus*	1	0	0	1	0	0	-	0	0	0	-	-	-	1	2	1	2	0	1	0
Cuclotocephalus extraneus	1	1	?	?	1	?	0	0	0	0	0	0	0	0	-	-	-	0	0	1
Cuclotogaster madagascariensis	1	0	1	1	1	2	0	0	0	0	1	1	0	1	0	-	-	0	0	1
Cuculicola atopus*	1	1	1	1	0	?	0	0	0	0	-	-	-	1	2	1	2	0	0	1
Cuculoecus latifrons	0	1	0	0	0	?	-	0	0	0	-	-	-	1	2	1	2	0	0	1
Cummingsiella ambigua	1	0	1	1	0	?	-	0	0	0	-	-	-	1	2	1	0	0	0	1
Dahlemhornia asymmetrica	1	0	0	1	0	?	-	0	0	0	-	-	-	1	2	1	0	0	1	1
Damalinia (Damalinia) crenelata	1	0	2	0	1	0	0	0	1	1	-	-	-	1	2	1	0	0	0	0
Degeeriella rufa*	1	1	1	1	0	0	-	0	0	0	-	-	-	1	2	1	0	0	1	1
Discocorpus c. cephalosus*	1	0	1	1	0	0	-	0	0	0	-	-	-	1	2	0	-	0	1	0

	41	42	43	44	45	46	47	48	49	50	51	52	53	54	55	56	57	58	59	60
Docophoroides brevis*	1	1	1	1	1	0	0	0	0	0	1	1	0	1	2	1	2	0	1	0
Echinophilopterus protrusus	0	0	0	0	0	?	-	0	0	0	-	-	-	1	2	1	0	0	0	0
Emersoniella bracteata	1	0	1	1	0	?	-	0	0	0	-	-	-	1	2	1	0	0	0	1
Episbates pederiformis	1	1	1	1	0	?	-	0	0	0	-	-	-	1	2	0	-	0	1	0
Esthiopterum giganteum	1	1	1	1	1	?	1	0	0	0	1	1	0	1	2	1	0	0	1	0
Falcolipeurus affulgeus*	1	0	0	1	1	2	1	0	0	0	1	1	0	1	2	1	0	0	0	1
Falcolius elbeli	1	0	1	1	1	?	0	0	0	0	1	1	0	1	2	1	0	0	0	0
Felicola (F.) viverriculae*	1	0	2	1	1	0	0	1	0	1	-	-	-	1	2	0	-	0	0	0
Forficuloecus emersoni*	1	0	0	1	0	?	-	0	0	0	-	-	-	1	2	1	1	0	0	0
Formicaphagus pittasomae	0	0	0	0	0	?	-	0	0	0	-	-	-	1	1	1	2	0	0	0
Formicaricola willisi	1	0	1	1	0	?	-	0	0	0	-	-	-	1	2	1	0	0	0	0
Fulicoffula longipila	1	1	0	0	1	?	0	0	0	0	1	1	0	1	2	1	0	0	0	0
Furnariphilus pagei	1	1	1	1	1	?	0	0	0	0	0	0	0	1	2	1	0	0	0	0
Geomydoecus (G.) heaneyi	1	0	2	1	1	0	0	0	0	1	-	-	-	1	2	0	-	0	0	0
Goniocotes gallinae*	1	0	1	1	0	0	-	0	0	0	-	-	-	0	-	-	-	1	1	0
Goniodes kéleri	1	0	1	1	1	0	0	0	0	0	1	0	0	0	-	-	-	1	1	1
Goniodes pavonis	1	0	1	1	1	0	1	0	0	0	1	1	0	0	-	-	-	1	1	0
Haffneria grandis	1	1	1	1	1	1	1	0	0	0	1	1	0	1	2	1	1	0	1	0
Halipeurus pelagicus	1	1	1	1	1	2	0	0	0	0	1	1	0	1	2	0	-	1	1	0
Harrisoniella copei	1	1	0	1	1	?	1	0	0	0	1	1	0	1	2	1	1	0	1	0
Harrisoniella hopkinsi*	1	1	0	1	1	1	1	0	0	0	1	1	0	0	-	-	-	0	1	0
Heptapsogaster temporalis	1	0	1	1	1	?	0	0	0	0	1	1	0	0	-	-	-	1	1	0
Hopkinsiella clavigera	0	1	?	?	0	?	-	0	0	0	-	-	-	0	-	-	-	0	0	1
Ibidoecus plataleae*	0	1	0	0	0	0	-	0	0	0	-	-	-	1	2	0	-	0	0	2
Incidifrons fulicae*	1	1	0	1	0	?	-	0	0	0	-	-	-	1	2	1	2	0	0	0
Incidifrons transpositus	1	1	1	1	0	?	-	0	0	0	-	-	-	1	1	1	2	0	0	0

	41	42	43	44	45	46	47	48	49	50	51	52	53	54	55	56	57	58	59	60
Kelloggia coniceps	1	0	1	1	0	?	-	0	0	0	-	-	-	0	-	-	-	0	1	0
Kodocephalon latum	1	0	?	?	1	?	0	0	0	0	0	0	1	0	-	-	-	1	1	0
Labicotes guttatus	1	0	?	?	0	?	-	0	0	0	-	-	-	0	-	-	-	1	1	0
Lagopoecus affinis	1	1	1	1	0	0	-	0	0	0	-	-	-	1	2	1	0	0	1	1
Lamprocorpus hirsutus	1	0	0	1	1	?	0	0	0	0	1	0	0	0	-	-	-	0	1	0
Lipeurus caponis	1	1	1	1	1	2	1	0	0	0	1	0	0	1	2	0	-	0	0	1
Lunaceps numenii	1	1	1	1	0	?	-	0	0	0	-	-	-	1	2	1	0	0	0	1
Megaginus sordidus	1	0	0	1	0	?	-	0	0	0	-	-	-	1	2	1	2	0	1	0
Megapeostus asymmetricus	1	0	1	1	1	?	0	0	0	0	1	1	0	0	-	-	-	1	1	0
Megapodiella nakatae	1	1	?	?	0	?	-	0	0	0	-	-	-	1	1	1	0	0	0	1
Meinertzhageniella lata	1	0	?	?	1	?	0	0	0	0	1	1	0	1	2	1	0	0	0	1
Meropoecus meropis*	0	1	0	0	0	?	-	0	0	0	-	-	-	1	1	1	2	0	0	0
Meropsiella* sp.	1	0	?	?	0	?	-	0	0	0	-	-	-	1	2	1	2	0	0	0
***Mulcticola* sp.**	1	1	1	1	0	?	-	0	0	0	-	-	-	1	1	1	0	0	0	1
Naubates fuliginosus	1	1	1	1	1	2	0	0	0	0	1	1	0	1	2	1	0	0	0	0
Neophilopterus heteropygus	0	1	0	0	0	?	-	0	0	0	-	-	-	1	1	1	2	0	1	0
Neopsittaconirmus borgiolii	1	0	1	1	1	0	0	0	0	0	0	0	0	1	2	1	0	0	0	0
Nesiotinus demersus	0	0	?	?	1	?	0	0	0	0	1	0	0	1	2	0	-	0	1	0
Nothocotus subsimilis	1	0	?	?	1	?	0	0	0	0	1	0	0	0	-	-	-	0	0	0
Nyctibicola longirostris	1	1	?	?	0	?	-	0	0	0	-	-	-	1	2	1	2	0	0	?
Ornicholax robustus	1	0	1	1	0	?	-	0	0	0	-	-	-	0	-	-	-	1	1	1
Ornithobius goniopleurus	0	0	0	0	1	?	0	0	0	0	1	0	0	1	2	1	0	0	1	0
Osculotes curtus	1	0	?	1	0	0	-	0	0	0	-	-	-	1	1	1	0	0	0	0
Osculotes macropoda*	1	0	?	1	0	0	-	0	0	0	-	-	-	1	1	1	0	0	0	0
***Otidoecus* sp.**	1	1	1	1	1	?	0	0	0	0	0	0	0	0	-	-	-	0	0	1
Oxylipeurus dentatus*	1	1	1	1	1	2	0	0	0	0	1	0	0	1	2	0	-	0	1	0

	41	42	43	44	45	46	47	48	49	50	51	52	53	54	55	56	57	58	59	60
Pachyskelotes orthopleurus	1	0	1	1	1	?	1	0	0	0	1	1	0	0	-	-	-	1	1	0
Paraclisis diomedea	1	1	1	1	1	2	0	0	0	0	1	1	0	1	2	1	1	0	1	0
Paragoniocotes rotundus	1	0	1	1	0	0	-	0	0	0	-	-	-	1	1	1	0	0	0	0
Paragoniocotes venezolanus	1	0	1	1	1	?	0	0	0	0	0	0	0	1	2	1	2	0	0	0
Paroncophorus javanicus	0	1	?	?	1	?	0	0	0	0	0	0	0	1	1	1	2	0	0	1
Passonomedea hopkinsi	1	0	1	1	1	?	0	0	0	0	1	0	0	0	-	-	-	0	0	0
Pectenosoma verrucosa	1	0	1	1	0	?	-	0	0	0	-	-	-	0	-	-	-	1	1	0
Pectinopygus bassani*	1	0	0	1	1	0	0	0	0	0	1	0	0	1	2	1	0	0	1	0
Pectinopygus sulae	1	0	?	?	1	2	0	0	0	0	1	0	0	1	2	1	0	0	1	0
Pelmatocerandra setosa	1	1	1	1	1	2	0	0	0	0	1	1	0	1	2	1	2	0	0	0
Penenirmus auritus	1	0	1	1	0	?	-	0	0	0	-	-	-	1	2	1	2	0	0	1
Perineus nigrolimbatus	1	1	1	1	1	2	0	0	0	0	1	1	0	1	2	1	1	0	0	0
Pessoaiella absita*	1	0	0	1	1	?	0	0	0	0	1	0	0	1	2	1	0	0	1	0
Philoceanus garrodiae	1	1	1	1	1	2	0	0	0	0	1	0	0	1	2	1	1	0	0	0
Philopterus ornatus	1	0	1	1	0	0	-	0	0	0	-	-	-	1	2	1	2	0	0	1
Physconella kelloggi	1	0	?	?	0	?	-	0	0	0	-	-	-	0	-	-	-	0	0	1
Physconelloides cubanus	1	0	1	1	0	?	-	0	0	0	-	-	-	0	-	-	-	1	1	0
Picicola snodgrassi*	0	1	0	0	0	?	-	0	0	0	-	-	-	1	2	1	0	0	0	1
Podargoecus strigoides	1	0	1	1	0	0	-	0	0	0	-	-	-	1	2	1	0	0	0	1
Pseudocophorus perijanus	0	0	?	?	1	?	0	0	0	0	1	1	0	1	2	1	2	0	0	0
Pseudolipeurus similis*	1	1	?	?	1	?	0	0	0	0	0	0	0	1	2	1	2	0	0	0
Pseudonirmus gurlti	1	1	1	1	1	0	0	0	0	0	1	0	0	1	2	1	1	0	0	0
Pseudophilopterus hirsutus*	1	0	?	?	1	?	0	0	0	0	0	0	0	1	2	1	0	0	0	1
Psittaconirmus (P.) zinki*	1	0	?	?	1	?	0	0	0	0	1	1	0	1	2	1	0	1	1	0
Psittoecus vanzolinii*	1	0	0	1	1	?	0	0	0	0	0	0	0	1	1	1	0	0	0	0
Pterocotes aberrans	1	0	1	1	1	?	0	0	0	0	1	1	0	0	-	-	-	0	1	0

	**41**	**42**	**43**	**44**	**45**	**46**	**47**	**48**	**49**	**50**	**51**	**52**	**53**	**54**	**55**	**56**	**57**	**58**	**59**	**60**
Quadraceps coenocoryphae*	1	1	1	1	0	0	-	0	0	0	-	-	-	1	2	1	2	0	1	1
Rallicola lugens	1	1	1	1	1	2	1	0	0	0	1	0	0	1	2	1	2	0	1	0
Rhopaloceras rudimentarius	1	0	1	1	1	?	0	0	0	0	0	0	0	0	-	-	-	1	1	0
Rhynonirmus scolopacis	1	1	1	1	1	?	0	0	0	0	0	0	0	1	2	1	0	0	0	2
Rotundiceps cordatus	1	0	1	1	0	?	-	0	0	0	-	-	-	1	1	1	2	0	1	1
Saemundssonia desolata*	1	1	1	1	0	0	-	0	0	0	-	-	-	1	2	1	2	0	1	1
Saemundssonia haematopi	1	1	1	1	0	0	-	0	0	0	-	-	-	1	2	1	2	0	1	1
Splendoroffula ruwenzorornis	1	1	?	?	1	2	0	0	0	0	1	0	0	1	1	1	1	0	1	0
Strigiphilus vapidus*	1	0	0	1	0	0	-	0	0	0	-	-	-	1	2	1	2	0	1	1
Strongylocotes angulocapitis*	1	1	?	?	1	0	0	0	0	0	0	0	0	1	1	0	-	1	1	0
Strongylocotes complanatus	1	1	1	1	0	?	-	0	0	0	-	-	-	1	1	1	0	1	1	0
Struthiolipeurus struthionis*	1	0	0	1	1	?	0	0	0	0	1	1	0	1	2	1	0	0	0	1
Sturnidoecus sturni*	1	0	0	1	0	0	-	0	0	0	-	-	-	1	2	1	1	0	1	0
Syrrhaptoecus falcatus	1	1	1	1	0	0	-	0	0	0	-	-	-	1	2	1	0	0	1	1
Theresiella gemina	1	0	0	1	1	?	0	0	0	0	0	0	0	1	2	1	0	0	1	1
***Tinamotaecola* sp.**	1	0	?	?	0	?	-	0	0	0	-	-	-	1	2	1	0	0	0	1
Trabeculus schillingi	1	1	1	1	1	0	1	0	0	0	1	0	0	1	2	0	-	0	1	0
Trichodectes (T.) melis*	1	0	2	1	1	0	0	1	0	1	-	-	-	1	2	0	-	0	0	1
***Trichodopeostus* sp.**	1	0	1	1	1	?	0	0	0	0	1	0	0	1	1	0	-	1	1	0
Trichophilopterus babakotophilus	0	0	2	0	0	?	-	0	1	0	-	-	-	1	2	1	1	1	1	0
Trogoniella aequatoriale	1	1	?	?	0	?	-	0	0	0	-	-	-	1	0	-	-	0	0	0
Trogoninirmus strigilatus	1	1	?	?	0	?	-	0	0	0	-	-	-	1	1	1	0	0	0	0
Turnicola angustissimus	1	1	1	1	0	?	-	0	0	0	-	-	-	0	-	-	-	0	0	1
Turturicola salimalii	1	1	?	?	1	?	0	0	0	0	0	0	0	1	0	-	-	0	0	0
Upupicola upupae	1	1	1	1	0	0	-	0	0	0	-	-	-	1	2	1	0	0	0	1
Vernoniella guimaraesi*	1	0	1	1	1	2	0	0	0	0	1	0	0	1	2	1	2	0	0	0

	61	62	63	64	65	66	67	68	69	70	71	72	73	74	75	76	77	78	79	80
Acidoproctus hilli	0	1	2	1	0	-	-	-	0	0	3	2	-	1	2	0	0	0	-	1
Acidoproctus rostratus	0	1	2	1	0	-	-	-	0	1	3	2	-	1	2	0	0	0	-	1
Acutifrons chimango	2	0	2	1	1	-	-	-	1	0	0	1	0	0	-	1	0	1	-	1
Aegypoecus brevicollis	1	1	2	3	-	0	-	-	?	1	0	1	1	1	2	0	1	0	-	?
Alcedoecus delphax*	0	1	2	1	1	-	-	-	0	1	1	1	0	1	0	0	0	0	-	1
Alcedoffula alcyonae*	0	0	2	1	1	-	-	-	0	0	1	1	1	1	0	0	1	1	0	1
Anaticola crassicornis*	0	1	2	5	-	-	0	-	0	1	3	1	1	1	1	0	1	1	1	1
Anatoecus icterodes*	0	1	2	5	-	-	1	-	0	1	0	2	-	1	-	0	0	0	-	1
Aquanirmus australis	0	1	2	5	-	-	0	-	0	1	0	2	-	0	-	0	1	0	-	1
Archolipeurus nandu	2	1	2	3	-	2	-	-	1	1	3	1	1	1	2	0	0	0	-	1
Ardeicola elongata	0	1	2	5	-	-	1	-	1	1	3	2	-	0	-	0	0	0	-	1
Ardeicola smithersi*	0	1	2	5	-	-	0	-	1	1	3	2	-	1	1	0	?	?	?	1
Ardeiphagus cochlearius	0	1	2	1	1	-	-	-	0	0	0	1	0	1	1	1	1	0	-	1
Auricotes affinis	0	1	2	1	1	-	-	-	0	0	0	1	0	1	2	0	0	0	-	1
Austrogoniodes waterstoni	0	1	2	6	-	-	-	-	1	1	1	1	0	1	2	0	0	0	-	0
Austrophilopterus spinosus	0	0	2	1	1	-	-	-	0	1	0	1	0	0	-	1	1	1	0	1
Bedfordiella unica	0	1	2	5	-	-	1	-	1	1	3	2	-	1	1	0	1	1	1	1
Bizarrifrons magus	0	1	2	5	-	-	1	-	0	1	0	1	0	1	2	1	1	1	0	1
Bothriometopus macrocnemis	0	1	2	5	-	-	1	-	0	1	3	2	-	1	2	0	0	0	-	1
Bovicola limbatus*	0	1	2	0	-	-	-	-	0	0	2	4	-	1	2	0	0	0	-	1
Brueelia ornatissima	0	1	2	5	-	-	1	-	0	0	0	1	0	1	2	1	1	1	0	1
Brueelia semiannulata*	0	1	2	5	-	-	1	-	0	0	0	1	0	1	2	0	1	1	0	1
Bucerocophorus pachycnemis	0	0	2	2	-	-	-	-	0	1	0	1	0	0	-	0	0	0	-	1
Buceroemersonia clarkei	0	1	?	?	?	?	?	?	0	?	0	1	0	0	-	0	0	0	-	1
***Buceronirmus* sp.**	0	1	2	2	-	-	-	-	0	1	1	1	1	1	0	0	1	0	-	1
Buceronirmus deignani	0	1	2	2	-	-	-	-	0	1	1	1	1	1	0	0	1	0	-	1

	61	62	63	64	65	66	67	68	69	70	71	72	73	74	75	76	77	78	79	80
Bucorvellus docophorus	0	0	2	1	1	-	-	-	0	?	0	1	0	0	-	0	0	0	-	1
Buerelius longiceps	0	0	2	5	-	-	1	-	0	1	0	1	1	1	2	0	0	1	-	1
Campanulotes compar*	2	1	2	1	1	-	-	-	0	0	0	1	0	1	2	0	0	0	-	0
Capraiella **sp.**	0	1	2	1	1	-	-	-	0	1	0	1	0	0	-	0	1	1	0	1
Capraiella sabzak	0	1	2	1	1	-	-	-	0	1	0	1	0	0	-	0	1	1	0	1
Carduiceps cingulatus	0	1	2	1	0	-	-	-	0	0	1	1	0	1	2	0	1	1	0	1
Centropodiella borneoensis	?	?	?	?	?	?	?	?	0	1	0	?	?	1	2	0	1	0	-	1
Chelopistes guttatus*	2	1	2	3	-	0	-	-	1	0	0	1	0	1	2	0	1	0	-	1
Chelopistes meleagridis	0	1	2	5	-	-	1	-	1	0	0	1	0	1	2	0	1	0	-	1
Cirrophthirius testudinarius	0	1	2	1	1	-	-	-	0	1	0	1	0	1	2	0	1	1	0	1
Colilipeurus colius	0	1	1	-	-	-	-	-	0	0	1	1	1	0	-	0	1	?	0	1
Colinicola mearnsi	0	1	2	1	1	-	-	-	0	1	1	1	0	0	-	0	1	0	-	1
Coloceras damicorne*	2	1	2	1	1	-	-	-	0	0	0	1	0	1	2	0	0	0	-	1
Columbicola columbae*	0	1	0	-	-	-	-	-	1	0	0	2	-	0	-	0	1	1	1	1
Cotingacola rupicolae	0	1	2	1	1	-	-	-	0	1	1	1	0	0	-	0	1	1	0	1
Craspedonirmus colymbinus	0	1	2	3	-	0	-	-	0	0	3	1	0	1	2	0	1	1	0	1
Craspedorrhynchus platystomus*	0	1	2	3	-	0	-	-	0	1	0	1	0	1	2	1	0	0	-	1
Cuclotocephalus extraneus	0	1	2	1	1	-	-	-	0	0	0	1	0	1	0	0	0	0	-	1
Cuclotogaster madagascariensis	0	1	2	1	1	-	-	-	1	0	1	1	1	0	-	0	1	0	-	1
Cuculicola atopus*	0	1	2	1	1	-	-	-	0	1	1	1	1	0	-	0	1	0	-	1
Cuculoecus latifrons	0	1	2	1	1	-	-	-	0	1	1	1	0	1	2	0	1	0	-	1
Cummingsiella ambigua	0	0	2	1	1	-	-	-	0	1	1	1	0	1	2	0	1	0	-	1
Dahlemhornia asymmetrica	0	1	2	?	?	?	?	?	0	0	0	1	0	0	-	0	1	1	0	1
Damalinia (Damalinia) crenelata	0	1	2	0	-	-	-	-	0	0	2	4	-	1	2	0	0	0	-	1
Degeeriella rufa*	0	1	2	1	1	-	-	-	0	0	0	1	0	0	-	0	1	1	0	1
Discocorpus c. cephalosus*	0	1	2	4	-	-	-	-	0	0	0	1	0	1	4	0	0	0	-	1

	61	62	63	64	65	66	67	68	69	70	71	72	73	74	75	76	77	78	79	80
Docophoroides brevis*	0	0	3	-	-	-	-	-	1	1	1	3	-	0	-	0	0	0	-	1
Echinophilopterus protrusus	0	0	2	1	0	-	-	-	0	1	0	1	0	1	2	0	1	0	-	1
Emersoniella bracteata	0	0	2	1	1	-	-	-	0	1	1	1	0	0	-	0	1	0	-	1
Episbates pederiformis	0	0	2	0	-	-	-	-	1	1	3	2	-	1	1	0	1	1	1	1
Esthiopterum giganteum	0	1	2	5	-	-	1	-	0	1	0	2	-	1	1	0	1	1	1	1
Falcolipeurus affulgeus*	0	1	1	-	-	-	-	-	0	0	3	2	-	0	-	0	0	0	-	1
Falcolius elbeli	0	1	1	-	-	-	-	-	0	0	0	1	0	0	-	0	1	1	0	1
Felicola (F.) viverriculae*	0	1	2	0	-	-	-	-	0	0	2	2	-	1	3	0	0	0	-	0
Forficuloecus emersoni*	0	1	2	7	-	-	-	-	1	1	0	1	0	1	2	1	0	0	-	1
Formicaphagus pittasomae	0	1	2	5	-	-	0	-	0	0	1	1	0	1	2	0	1	1	0	1
Formicaricola willisi	0	1	2	5	-	-	0	-	0	0	1	1	0	0	-	0	1	1	0	1
Fulicoffula longipila	0	1	2	5	-	-	1	-	1	1	1	2	-	1	1	0	1	1	0	1
Furnariphilus pagei	0	1	1	-	-	-	-	-	0	0	0	1	0	0	-	0	1	1	0	1
Geomydoecus (G.) heaneyi	0	1	2	0	-	-	-	-	0	0	2	2	-	1	3	0	0	0	-	0
Goniocotes gallinae*	0	1	2	1	1	-	-	-	0	0	0	1	0	1	2	0	0	0	-	1
Goniodes kéleri	2	1	2	1	1	-	-	-	0	0	0	1	0	1	2	0	0	0	-	0
Goniodes pavonis	2	1	2	1	1	-	-	-	0	1	0	1	0	1	2	1	0	0	-	1
Haffneria grandis	0	1	2	5	-	-	1	-	1	0	3	2	-	1	1	0	1	1	1	1
Halipeurus pelagicus	0	1	2	5	-	-	1	-	0	1	3	1	1	1	1	0	1	1	1	1
Harrisoniella copei	0	1	2	5	-	-	1	-	1	0	3	2	-	1	1	0	1	1	1	1
Harrisoniella hopkinsi*	0	1	2	5	-	-	1	-	1	0	3	2	-	1	1	0	1	1	1	1
Heptapsogaster temporalis	0	1	2	2	-	-	-	-	0	0	0	1	0	1	2	0	0	0	-	1
Hopkinsiella clavigera	0	0	2	2	-	-	-	-	0	1	1	1	0	0	-	0	1	1	0	1
Ibidoecus plataleae*	2	1	2	9	-	-	-	2	0	1	1	2	-	1	1	0	1	0	-	1
Incidifrons fulicae*	0	1	2	1	1	-	-	-	0	1	1	1	0	1	0	0	0	0	-	1
Incidifrons transpositus	0	1	2	1	1	-	-	-	0	1	0	1	0	1	0	0	0	1	-	1

	61	62	63	64	65	66	67	68	69	70	71	72	73	74	75	76	77	78	79	80
Kelloggia coniceps	0	1	2	2	-	-	-	-	0	0	0	1	0	1	2	0	1	0	-	0
Kodocephalon latum	0	1	2	1	1	-	-	-	0	0	0	1	0	1	2	0	0	0	-	0
Labicotes guttatus	0	1	2	1	1	-	-	-	0	0	0	1	0	1	4	0	0	0	-	1
Lagopoecus affinis	0	1	2	1	1	-	-	-	0	1	1	1	1	0	-	0	1	1	0	1
Lamprocorpus hirsutus	0	1	2	2	-	-	-	-	0	0	0	1	0	1	2	0	0	0	-	1
Lipeurus caponis	0	0	1	-	-	-	-	-	1	0	1	1	1	0	-	0	1	1	0	1
Lunaceps numenii	0	0	2	1	1	-	-	-	0	1	1	1	0	0	-	0	1	1	0	1
Megaginus sordidus	0	1	2	1	1	-	-	-	0	0	0	1	0	1	4	0	0	0	-	1
Megapeostus asymmetricus	0	1	2	2	-	-	-	-	0	0	0	1	0	1	4	0	0	0	-	1
Megapodiella nakatae	0	1	2	1	1	-	-	-	0	1	0	1	0	1	1	0	1	0	-	1
Meinertzhageniella lata	1	1	3	-	-	-	-	-	1	1	1	1	1	1	2	0	0	0	-	1
Meropoecus meropis*	0	1	2	5	-	-	1	-	0	1	0	1	0	1	2	0	0	0	-	1
Meropsiella* sp.	0	1	2	5	-	-	1	-	0	0	0	1	0	1	0	0	1	1	0	1
***Mulcticola* sp.**	0	1	2	1	1	-	-	-	0	1	0	1	0	1	0	0	1	1	0	1
Naubates fuliginosus	0	1	2	3	-	1	-	-	1	1	3	2	-	1	1	0	1	1	1	1
Neophilopterus heteropygus	0	1	2	9	-	-	-	3	1	0	1	2	-	1	0	0	0	0	-	1
Neopsittaconirmus borgiolii	0	1	2	5	-	-	0	-	0	0	1	1	1	0	-	0	1	0	-	1
Nesiotinus demersus	0	1	2	9	-	-	-	4	?	1	1	2	-	1	1	0	1	1	0	1
Nothocotus subsimilis	0	1	2	1	1	-	-	-	0	0	0	1	0	1	4	0	0	0	-	1
Nyctibicola longirostris	?	?	?	?	?	?	?	?	0	1	0	?	?	1	0	0	0	0	-	1
Ornicholax robustus	0	1	2	2	-	-	-	-	0	0	0	1	0	1	2	0	0	0	-	0
Ornithobius goniopleurus	0	1	2	1	0	-	-	-	1	1	1	1	0	1	0	0	1	0	-	1
Osculotes curtus	2	1	2	9	-	-	-	0	0	0	0	1	0	1	2	0	1	0	-	1
Osculotes macropoda*	2	1	2	9	-	-	-	0	0	0	0	1	0	1	2	0	1	0	-	1
***Otidoecus* sp.**	0	1	2	1	1	-	-	-	0	0	0	1	0	0	-	0	1	1	0	1
Oxylipeurus dentatus*	0	1	2	5	-	-	1	-	0	0	0	2	-	0	-	0	1	0	-	1

	61	62	63	64	65	66	67	68	69	70	71	72	73	74	75	76	77	78	79	80
Pachyskelotes orthopleurus	0	1	2	1	1	-	-	-	0	1	0	1	0	1	2	0	0	0	-	1
Paraclisis diomedea	0	1	2	5	-	-	1	-	1	1	3	2	-	1	1	0	1	1	1	1
Paragoniocotes rotundus	0	0	2	9	-	-	-	1	0	0	1	1	1	?	?	0	1	0	-	1
Paragoniocotes venezolanus	0	1	2	3	-	-	1	-	0	0	0	1	0	0	-	0	1	0	-	1
Paroncophorus javanicus	0	0	2	5	-	-	1	-	1	1	0	1	0	0	-	0	0	0	-	1
Passonomedea hopkinsi	0	1	2	1	1	-	-	-	0	1	0	1	0	1	2	0	0	0	-	1
Pectenosoma verrucosa	0	1	2	2	-	-	-	-	0	0	0	1	0	1	2	0	0	0	-	1
Pectinopygus bassani*	0	1	2	1	1	-	-	-	0	1	1	2	-	1	1	0	1	1	0	1
Pectinopygus sulae	0	1	2	1	1	-	-	-	0	1	3	1	1	1	1	1	1	1	0	1
Pelmatocerandra setosa	0	1	2	7	-	-	-	-	1	1	3	2	-	1	1	0	1	1	1	1
Penenirmus auritus	3	-	2	1	1	-	-	-	0	1	0	1	0	0	-	0	0	0	-	1
Perineus nigrolimbatus	0	1	2	5	-	-	1	-	1	1	3	2	-	1	1	0	1	1	1	1
Pessoaiella absita*	0	1	2	5	-	-	0	-	1	1	0	1	0	1	2	0	1	1	0	1
Philoceanus garrodiae	0	1	2	5	-	-	1	-	1	1	3	2	-	1	1	0	1	1	1	1
Philopterus ornatus	1	1	2	3	-	0	-	-	0	1	0	1	0	1	2	1	0	0	-	1
Physconella kelloggi	2	1	3	-	-	-	-	-	0	1	0	1	0	1	2	0	0	0	-	1
Physconelloides cubanus	0	1	2	1	1	-	-	-	0	1	0	1	0	1	2	0	0	0	-	1
Picicola snodgrassi*	0	0	2	1	1	-	-	-	1	1	0	1	0	0	-	0	1	0	-	1
Podargoecus strigoides	0	1	2	1	1	-	-	-	0	1	0	1	0	0	-	0	0	0	-	1
Pseudocophorus perijanus	0	1	2	5	-	-	1	-	0	0	0	2	-	1	2	0	0	0	-	1
Pseudolipeurus similis*	0	1	2	5	-	-	1	-	0	1	0	1	0	1	1	0	1	1	?	1
Pseudonirmus gurlti	0	1	2	5	-	-	1	-	1	1	3	2	-	1	1	0	1	1	1	1
Pseudophilopterus hirsutus*	0	1	2	1	1	-	-	-	0	1	0	1	1	0	-	0	0	0	-	1
Psittaconirmus (P.) zinki*	0	1	2	5	-	-	1	-	0	0	1	1	1	0	-	1	1	0	-	1
Psittoecus vanzolinii*	0	1	2	1	1	-	-	-	1	0	1	1	1	1	0	0	0	0	-	1
Pterocotes aberrans	0	1	2	2	-	-	-	-	0	1	0	1	0	1	2	0	0	0	-	1

	61	62	63	64	65	66	67	68	69	70	71	72	73	74	75	76	77	78	79	80
Quadraceps coenocoryphae*	0	0	2	1	1	-	-	-	0	1	3	1	0	1	2	0	0	0	-	1
Rallicola lugens	0	1	2	5	-	-	1	-	0	1	0	1	0	1	2	0	1	0	-	1
Rhopaloceras rudimentarius	0	1	2	2	-	-	-	-	0	1	0	1	0	1	2	0	1	1	-	1
Rhynonirmus scolopacis	0	1	2	1	1	-	-	-	0	1	0	1	0	0	-	0	1	0	-	1
Rotundiceps cordatus	0	0	2	1	1	-	-	-	0	1	0	1	0	0	-	0	1	0	-	1
Saemundssonia desolata*	0	0	2	2	-	-	-	-	0	1	1	1	0	1	0	1	0	0	-	1
Saemundssonia haematopi	0	0	2	2	-	-	-	-	1	1	1	1	0	1	0	1	0	0	-	1
Splendoroffula ruwenzorornis	0	1	1	-	-	-	-	-	0	0	0	2	-	0	-	0	1	1	-	1
Strigiphilus vapidus*	0	1	2	1	1	-	-	-	0	1	0	1	0	1	2	0	0	0	-	1
Strongylocotes angulocapitis*	2	1	2	8	-	-	-	-	1	0	0	1	0	1	2	0	0	0	-	0
Strongylocotes complanatus	0	1	2	5	-	-	1	-	1	1	0	1	0	1	2	0	0	0	-	0
Struthiolipeurus struthionis*	2	1	2	3	-	2	-	-	1	1	3	1	1	1	2	0	0	0	-	1
Sturnidoecus sturni*	0	1	2	5	-	-	1	-	0	1	0	1	0	1	2	0	0	0	-	1
Syrrhaptoecus falcatus	0	1	2	1	1	-	-	-	0	0	0	1	1	0	-	0	0	0	-	1
Theresiella gemina	0	1	2	5	-	-	1	-	0	1	0	1	1	1	2	0	1	0	-	1
***Tinamotaecola* sp.**	0	1	2	1	1	-	-	-	0	0	0	1	0	0	-	0	1	0	-	1
Trabeculus schillingi	0	1	2	8	-	-	-	-	1	1	1	0	-	0	-	0	0	0	-	1
Trichodectes (T.) melis*	2	1	2	5	-	-	1	-	0	0	2	2	-	1	3	0	0	0	-	0
***Trichodopeostus* sp.**	0	1	2	8	-	-	-	-	0	1	0	1	0	1	2	0	0	0	-	1
Trichophilopterus babakotophilus	0	1	3	-	-	-	-	-	1	0	1	2	-	1	0	0	0	0	-	1
Trogoniella aequatoriale	0	1	2	5	-	-	1	-	0	0	0	1	1	0	-	0	1	0	-	1
Trogoninirmus strigilatus	0	1	2	2	-	-	-	-	0	0	0	1	0	0	-	0	1	0	-	1
Turnicola angustissimus	3	-	2	1	1	-	-	-	0	0	0	1	0	0	-	0	1	1	0	1
Turturicola salimalii	0	1	0	-	-	-	-	-	1	0	0	2	-	0	-	0	1	1	1	1
Upupicola upupae	0	0	2	1	1	-	-	-	0	0	0	1	0	0	-	0	0	0	-	1
Vernoniella guimaraesi*	0	1	2	1	1	-	-	-	0	1	1	1	0	1	2	0	0	0	-	1

	81	82	83	84	85	86	87	88	89	90	91	92	93	94	95	96	97	98	99	100
Acidoproctus hilli	3	0	1	1	-	1	1	0	0	1	1	-	3	-	0	2	0	1	0	0
Acidoproctus rostratus	3	0	1	1	-	1	1	0	0	1	1	-	3	-	0	2	0	1	0	0
Acutifrons chimango	3	0	1	1	-	1	0	0	1	1	0	1	-	-	0	2	0	0	-	0
Aegypoecus brevicollis	?	0	?	1	-	1	?	?	?	?	?	?	?	?	?	?	0	1	5	0
Alcedoecus delphax*	2	0	0	1	-	1	0	1	1	1	2	-	-	-	0	2	0	0	-	0
Alcedoffula alcyonae*	2	0	?	1	0	1	0	1	1	1	0	1	-	-	0	2	0	0	-	0
Anaticola crassicornis*	2	2	1	0	0	0	0	0	0	1	1	-	3	-	0	2	0	0	-	0
Anatoecus icterodes*	2	0	1	1	0	1	0	1	0	1	0	1	-	-	1	2	0	0	-	0
Aquanirmus australis	3	1	1	1	-	0	0	0	0	1	0	1	-	-	0	1	0	0	-	0
Archolipeurus nandu	2	0	1	1	-	0	1	0	1	1	1	-	2	-	0	3	0	1	3	0
Ardeicola elongata	1	2	1	0	0	0	1	0	0	1	1	-	4	-	0	2	0	1	0	0
Ardeicola smithersi*	1	2	1	0	0	0	1	0	0	1	1	-	4	-	0	2	0	1	0	0
Ardeiphagus cochlearius	2	1	1	0	0	0	0	0	0	?	0	1	-	-	?	3	0	0	-	0
Auricotes affinis	0	0	0	1	-	1	0	1	1	0	1	-	1	1	0	?	0	0	-	0
Austrogoniodes waterstoni	-	0	0	1	-	0	0	0	0	0	0	0	-	-	0	2	0	1	2	0
Austrophilopterus spinosus	3	0	0	1	-	1	0	0	1	1	1	-	7	-	0	2	0	0	-	0
Bedfordiella unica	2	2	?	0	0	0	1	0	0	1	1	-	4	-	0	2	0	1	0	0
Bizarrifrons magus	2	0	0	1	-	1	0	0	1	1	0	0	-	-	0	1	0	0	-	0
Bothriometopus macrocnemis	3	0	0	1	-	1	1	0	0	0	1	-	3	-	0	2	0	1	0	0
Bovicola limbatus*	0	0	0	0	1	0	0	0	0	0	0	0	-	-	1	1	0	1	1	0
Brueelia ornatissima	2	0	0	1	-	1	0	0	0	1	0	1	-	-	0	1	0	0	-	0
Brueelia semiannulata*	2	0	0	1	-	1	0	0	1	1	0	1	-	-	0	1	0	0	-	0
Bucerocophorus pachycnemis	4	0	1	1	-	1	0	0	1	1	1	-	1	0	0	2	0	0	-	0
Buceroemersonia clarkei	2	1	?	1	-	1	?	?	1	1	?	?	?	?	0	?	0	?	?	0
***Buceronirmus* sp.**	5	1	?	0	0	0	0	0	0	1	1	-	4	-	0	2	0	0	-	0
Buceronirmus deignani	5	1	?	0	0	0	0	0	0	1	1	-	4	-	0	2	0	0	-	0

	81	82	83	84	85	86	87	88	89	90	91	92	93	94	95	96	97	98	99	100
Bucorvellus docophorus	2	1	1	0	0	0	0	0	1	1	1	-	5	-	0	3	0	0	-	0
Buerelius longiceps	3	0	0	1	-	1	0	0	1	1	0	0	-	-	0	0	0	0	-	0
Campanulotes compar*	-	0	0	1	-	1	0	1	1	0	1	-	1	1	0	0	0	0	-	0
***Capraiella* sp.**	3	1	0	1	-	0	0	0	1	1	1	-	1	0	0	2	0	0	-	0
Capraiella sabzak	3	1	0	1	-	0	0	0	1	1	1	-	1	0	0	2	0	0	-	0
Carduiceps cingulatus	2	0	0	0	1	1	0	0	0	1	0	1	-	-	0	1	0	1	0	0
Centropodiella borneoensis	2	0	0	1	-	0	?	?	?	?	?	?	?	?	0	?	?	?	?	0
Chelopistes guttatus*	3	0	0	1	-	1	0	0	1	1	1	-	1	0	?	2	0	0	-	0
Chelopistes meleagridis	3	0	0	1	-	1	1	1	1	1	1	-	1	0	0	?	0	0	-	0
Cirrophthirius testudinarius	2	0	0	1	-	1	0	0	1	1	0	1	-	-	0	2	0	0	-	0
Colilipeurus colius	2	1	1	1	-	0	0	0	1	1	1	-	1	0	0	2	0	0	-	0
Colinicola mearnsi	2	1	1	1	-	0	0	0	1	1	0	1	-	-	0	2	0	0	-	0
Coloceras damicorne*	0	0	0	0	1	1	0	1	1	0	1	-	1	1	0	0	0	0	-	0
Columbicola columbae*	2	2	1	0	0	0	1	1	0	1	1	-	4	-	0	3	0	0	-	0
Cotingacola rupicolae	4	1	0	0	0	0	0	0	1	1	1	-	1	0	0	2	0	0	-	0
Craspedonirmus colymbinus	0	0	0	1	-	1	1	0	0	1	0	1	-	-	0	2	0	1	0	0
Craspedorrhynchus platystomus*	2	0	1	1	-	1	0	0	1	1	2	-	-	-	0	1	0	0	-	0
Cuclotocephalus extraneus	2	0	0	1	-	1	0	1	1	1	1	-	0	-	0	2	1	0	-	0
Cuclotogaster madagascariensis	2	1	1	0	0	0	0	0	1	1	1	-	1	0	0	2	0	0	-	0
Cuculicola atopus*	3	1	1	1	-	0	0	0	1	1	1	-	3	-	0	2	0	0	-	0
Cuculoecus latifrons	3	0	1	1	-	0	0	0	1	1	0	0	-	-	0	2	0	0	-	0
Cummingsiella ambigua	2	1	1	1	-	1	0	0	1	1	0	0	-	-	0	2	0	0	-	0
Dahlemhornia asymmetrica	2	0	0	0	1	0	0	0	1	1	0	2	-	-	0	2	0	1	5	0
Damalinia (Damalinia) crenelata	0	0	0	0	1	0	0	0	0	0	0	0	-	-	1	1	0	1	1	0
Degeeriella rufa*	3	1	0	0	0	0	0	0	1	1	1	-	1	0	0	2	0	0	-	0
Discocorpus c. cephalosus*	2	0	0	0	1	?	0	0	1	1	1	-	0	-	0	1	1	0	-	0

	81	82	83	84	85	86	87	88	89	90	91	92	93	94	95	96	97	98	99	100
*Docophoroides brevis**	3	0	1	1	-	1	1	0	0	1	0	1	-	-	0	2	0	1	2	0
Echinophilopterus protrusus	2	0	1	0	0	0	0	0	1	1	1	-	3	-	0	?	0	0	-	0
Emersoniella bracteata	3	1	0	1	-	1	0	0	1	1	0	0	-	-	0	2	0	0	-	0
Episbates pederiformis	2	2	1	0	0	0	1	0	0	1	1	-	4	-	0	2	0	1	0	0
Esthiopterum giganteum	2	2	0	1	-	0	0	0	0	1	0	1	-	-	0	2	0	0	-	0
*Falcolipeurus affulgeus**	0	1	1	0	0	0	1	0	1	1	1	-	2	-	0	3	0	1	3	0
Falcolius elbeli	2	1	1	0	0	0	0	0	1	1	1	-	1	0	0	2	0	1	?	0
*Felicola (F.) viverriculae**	-	0	0	1	-	0	0	0	0	1	0	1	-	-	1	1	0	1	1	1
*Forficuloecus emersoni**	2	2	1	1	-	1	0	1	1	1	0	0	-	-	0	1	0	0	-	0
Formicaphagus pittasomae	2	2	0	1	-	1	0	0	1	1	0	1	-	-	0	0	0	0	-	0
Formicaricola willisi	2	1	0	1	-	1	0	1	1	1	0	1	-	-	0	2	0	0	-	0
Fulicoffula longipila	2	2	1	0	0	0	1	0	1	1	1	-	4	-	0	2	0	1	0	0
Furnariphilus pagei	2	2	1	1	-	0	0	0	0	1	0	1	-	-	0	2	0	0	-	0
Geomydoecus (G.) heaneyi	-	0	0	0	1	0	0	0	0	1	0	0	-	-	1	1	0	1	1	1
*Goniocotes gallinae**	0	0	0	1	-	1	0	1	1	0	1	-	1	0	0	2	0	0	-	0
Goniodes kéleri	-	0	0	0	1	1	0	0	1	0	1	-	1	?	0	?	0	0	-	0
Goniodes pavonis	2	0	0	0	1	1	0	1	1	1	1	-	1	0	0	2	0	0	-	0
Haffneria grandis	2	2	1	0	0	0	1	0	0	1	1	-	4	-	0	2	0	1	0	0
Halipeurus pelagicus	2	2	1	0	0	0	1	0	0	1	1	-	2	-	0	1	0	1	0	0
Harrisoniella copei	2	2	1	0	0	0	1	0	0	1	1	-	6	-	0	2	0	1	0	0
*Harrisoniella hopkinsi**	2	2	1	0	0	0	1	0	0	1	1	-	6	-	0	2	0	1	0	0
Heptapsogaster temporalis	2	0	0	1	-	?	0	1	1	1	1	-	0	-	0	?	1	0	-	0
Hopkinsiella clavigera	3	0	1	1	-	1	0	0	1	1	0	2	-	-	0	2	0	0	-	0
*Ibidoecus plataleae**	3	0	1	1	-	1	0	0	1	1	0	0	-	-	0	2	0	1	4	0
*Incidifrons fulicae**	2	0	0	1	-	1	0	0	1	1	0	0	-	-	0	1	0	0	-	0
Incidifrons transpositus	2	0	1	1	-	1	0	0	1	1	0	0	-	-	0	1	0	0	-	0

	**81**	**82**	**83**	**84**	**85**	**86**	**87**	**88**	**89**	**90**	**91**	**92**	**93**	**94**	**95**	**96**	**97**	**98**	**99**	**100**
Kelloggia coniceps	-	0	0	1	-	0	0	1	1	0	1	-	0	-	0	2	1	0	-	0
Kodocephalon latum	-	0	0	1	-	0	0	1	1	1	1	-	1	0	0	0	0	0	-	0
Labicotes guttatus	2	0	0	1	-	1	?	?	1	1	1	-	1	0	0	0	0	0	-	0
Lagopoecus affinis	2	1	0	0	0	0	0	0	1	1	1	-	1	0	0	2	0	0	-	0
Lamprocorpus hirsutus	2	0	0	1	-	0	1	0	1	1	1	-	0	-	0	2	1	0	-	0
Lipeurus caponis	2	1	1	0	0	0	0	0	1	1	1	-	4	-	0	2	0	0	-	0
Lunaceps numenii	2	1	0	1	-	0	0	0	1	1	0	1	-	-	0	2	0	0	-	0
Megaginus sordidus	2	0	0	1	-	?	0	1	1	0	1	-	0	-	0	2	1	0	-	0
Megapeostus asymmetricus	2	0	0	1	-	?	0	1	1	0	1	-	0	-	0	2	1	0	-	0
Megapodiella nakatae	0	0	1	1	-	1	0	0	1	1	1	-	8	-	0	2	0	0	-	0
Meinertzhageniella lata	2	0	1	1	-	0	1	0	1	1	1	-	2	-	0	3	0	1	3	0
Meropoecus meropis*	2	0	0	1	-	1	0	1	1	1	0	0	-	-	0	?	0	0	-	0
Meropsiella* sp.	0	1	1	1	-	0	0	0	1	1	0	1	-	-	0	1	0	0	-	0
***Mulcticola* sp.**	2	2	1	1	-	0	0	0	1	1	1	-	1	0	0	2	0	0	-	0
Naubates fuliginosus	2	2	1	0	0	0	1	0	0	1	1	-	4	-	0	1	0	1	0	0
Neophilopterus heteropygus	2	1	1	0	0	0	0	0	1	1	1	-	2	-	0	2	0	1	4	0
Neopsittaconirmus borgiolii	2	1	0	1	-	0	0	1	0	1	1	-	4	-	0	2	0	0	-	0
Nesiotinus demersus	6	0	0	1	-	1	0	0	0	1	0	0	-	-	0	3	0	1	0	0
Nothocotus subsimilis	2	0	0	1	-	0	0	0	1	1	1	-	0	-	0	?	1	0	-	0
Nyctibicola longirostris	2	1	1	1	-	1	?	?	?	?	?	?	?	?	0	?	?	?	?	0
Ornicholax robustus	-	0	0	1	-	0	0	1	1	1	1	-	0	-	0	1	1	0	-	0
Ornithobius goniopleurus	3	0	1	1	-	1	1	0	0	1	1	-	3	-	0	2	0	1	0	0
Osculotes curtus	0	0	0	1	-	1	0	0	1	1	1	-	1	0	0	1	0	0	-	0
Osculotes macropoda*	0	0	0	1	-	1	0	0	1	1	1	-	1	0	0	1	0	0	-	0
***Otidoecus* sp.**	2	1	1	1	-	0	0	0	1	1	0	1	-	-	0	2	0	0	-	0
Oxylipeurus dentatus*	3	1	1	0	0	0	1	1	1	1	1	-	4	-	0	2	0	0	-	0

	81	82	83	84	85	86	87	88	89	90	91	92	93	94	95	96	97	98	99	100
Pachyskelotes orthopleurus	1	0	0	1	-	1	0	1	1	1	1	-	2	-	0	2	0	0	-	0
Paraclisis diomedea	2	2	1	0	0	0	1	0	0	1	1	-	4	-	0	2	0	1	0	0
Paragoniocotes rotundus	2	0	0	1	-	0	0	1	1	1	0	1	-	-	0	2	0	0	-	0
Paragoniocotes venezolanus	2	1	0	1	-	0	0	0	1	1	0	2	-	-	0	2	0	0	-	0
Paroncophorus javanicus	2	1	0	1	-	0	0	0	1	1	0	0	-	-	0	2	0	0	-	0
Passonomedea hopkinsi	2	0	0	1	-	1	0	1	1	0	1	-	1	0	0	1	0	0	-	0
Pectenosoma verrucosa	2	0	1	1	-	?	0	1	1	1	1	-	0	-	0	2	1	0	-	0
Pectinopygus bassani*	3	2	0	0	0	0	0	0	1	0	1	-	5	-	0	2	0	0	-	0
Pectinopygus sulae	3	2	0	0	0	0	0	0	1	0	1	-	5	-	0	2	0	0	-	0
Pelmatocerandra setosa	2	2	1	0	0	0	1	0	0	1	1	-	3	-	0	2	0	1	0	0
Penenirmus auritus	2	0	1	1	-	1	0	0	0	1	0	1	-	-	0	2	0	0	-	0
Perineus nigrolimbatus	2	2	1	0	0	0	1	0	0	1	1	-	4	-	0	2	0	1	0	0
Pessoaiella absita*	2	0	0	0	0	0	0	0	1	1	1	-	?	-	0	1	0	0	-	0
Philoceanus garrodiae	2	2	1	0	0	1	1	0	0	1	1	-	4	-	0	0	0	1	0	0
Philopterus ornatus	2	0	0	1	-	1	0	0	1	1	0	0	-	-	0	2	0	0	-	0
Physconella kelloggi	2	0	1	1	-	1	0	1	1	1	1	-	0	-	0	1	1	0	-	0
Physconelloides cubanus	2	0	0	0	1	1	0	1	1	0	1	-	1	0	0	1	0	0	-	0
Picicola snodgrassi*	2	1	0	1	-	0	0	0	1	1	0	1	-	-	0	2	0	0	-	0
Podargoecus strigoides	2	0	1	1	-	1	0	0	1	1	0	0	-	-	0	2	0	0	-	0
Pseudocophorus perijanus	2	0	0	1	-	1	1	0	1	1	0	1	-	-	0	?	0	0	-	0
Pseudolipeurus similis*	?	2	0	0	0	0	0	0	1	0	1	-	4	-	0	?	0	0	-	0
Pseudonirmus gurlti	2	2	0	0	0	0	1	0	0	1	1	-	4	-	0	1	0	1	0	0
Pseudophilopterus hirsutus*	2	1	1	0	0	0	0	0	1	1	1	-	4	-	0	2	0	0	-	0
Psittaconirmus (P.) zinki*	2	1	0	0	0	0	0	1	1	1	1	-	3	-	0	2	0	1	0	0
Psittoecus vanzolinii*	2	0	1	1	-	1	0	1	1	1	0	1	-	-	0	1	0	0	-	0
Pterocotes aberrans	2	0	0	1	-	?	1	0	1	1	1	-	0	-	0	0	1	0	-	0

	81	82	83	84	85	86	87	88	89	90	91	92	93	94	95	96	97	98	99	100
Quadraceps coenocoryphae*	2	0	0	1	-	1	1	1	0	1	0	0	-	-	0	2	0	0	-	0
Rallicola lugens	2	0	0	1	-	1	0	1	1	1	1	-	1	0	0	1	0	1	0	0
Rhopaloceras rudimentarius	6	0	0	0	1	0	0	1	1	1	1	-	0	-	0	2	0	0	-	0
Rhynonirmus scolopacis	2	2	0	1	-	0	0	0	1	1	0	1	-	-	0	?	0	0	-	0
Rotundiceps cordatus	2	0	0	1	-	1	0	0	1	1	0	1	-	-	0	2	0	0	-	0
Saemundssonia desolata*	0	0	0	1	-	1	0	1	0	1	0	0	-	-	0	1	0	0	-	0
Saemundssonia haematopi	0	0	0	1	-	1	0	1	0	1	0	0	-	-	0	1	0	0	-	0
Splendoroffula ruwenzorornis	2	1	1	0	0	0	1	1	1	1	1	-	4	-	0	2	0	0	-	0
Strigiphilus vapidus*	3	0	1	1	-	1	0	0	1	1	2	-	-	-	0	2	0	0	-	0
Strongylocotes angulocapitis*	-	0	0	1	-	1	0	1	1	1	1	-	0	-	0	2	1	0	-	0
Strongylocotes complanatus	-	0	1	1	-	1	0	1	1	1	1	-	0	-	0	0	1	0	-	0
Struthiolipeurus struthionis*	2	0	0	1	-	0	1	0	1	1	1	-	2	-	0	3	0	1	3	0
Sturnidoecus sturni*	3	0	0	1	-	1	0	0	1	1	0	0	-	-	0	1	0	0	-	0
Syrrhaptoecus falcatus	2	1	1	0	0	0	0	0	1	0	0	1	-	-	0	2	0	0	-	0
Theresiella gemina	2	0	0	1	0	1	0	1	1	1	0	1	-	-	0	2	0	1	2	0
***Tinamotaecola* sp.**	2	1	1	1	-	1	0	0	0	1	0	1	-	-	0	2	0	0	-	0
Trabeculus schillingi	2	0	0	1	-	1	1	0	0	1	0	1	-	-	0	2	0	1	0	0
Trichodectes (T.) melis*	-	0	0	0	1	0	0	0	0	1	0	0	-	-	1	1	0	1	1	0
***Trichodopeostus* sp.**	2	0	0	1	-	?	0	1	1	1	1	-	0	-	0	?	1	0	-	0
Trichophilopterus babakotophilus	2	0	0	0	1	0	0	1	0	0	1	-	9	-	1	1	0	1	?	0
Trogoniella aequatoriale	0	1	0	1	-	1	0	0	1	1	1	-	1	0	0	2	0	0	-	0
Trogoninirmus strigilatus	3	1	0	1	-	1	0	0	1	1	1	-	1	0	0	2	0	0	-	0
Turnicola angustissimus	2	2	1	0	0	0	0	0	0	1	1	-	1	0	0	3	0	0	-	0
Turturicola salimalii	2	2	1	0	0	0	1	1	0	1	1	-	4	-	0	?	0	0	-	0
Upupicola upupae	2	1	1	1	-	1	0	0	1	1	0	0	-	-	0	2	0	0	-	0
Vernoniella guimaraesi*	2	0	1	1	-	1	0	0	1	1	0	1	-	-	0	1	0	0	-	0

	101	102	103	104	105	106	107	108	109	110	111	112	113	114	115	116	117	118	119	120
Acidoproctus hilli	0	0	1	3	1	1	2	5	1	2	0	-	-	1	0	-	0	2	1	1
Acidoproctus rostratus	0	0	1	3	1	1	2	5	1	2	0	-	-	1	0	-	0	2	1	0
Acutifrons chimango	0	0	1	4	1	0	-	4	0	-	1	2	-	-	1	1	2	0	1	-
Aegypoecus brevicollis	0	0	?	3	1	0	-	2	0	-	1	0	1	-	1	1	0	0	1	-
Alcedoecus delphax*	0	0	1	3	1	0	-	2	0	-	1	2	-	-	1	1	1	0	1	-
Alcedoffula alcyonae*	0	0	1	3	1	1	0	2	1	0	0	-	-	3	0	-	2	2	1	1
Anaticola crassicornis*	0	0	1	3	1	1	2	2	1	2	0	-	-	1	0	-	1	1	2	-
Anatoecus icterodes*	0	0	1	3	3	0	-	2	0	-	0	-	-	3	0	-	0	0	1	-
Aquanirmus australis	0	0	1	3	1	1	2	2	1	0	0	-	-	0	0	-	0	0	2	-
Archolipeurus nandu	0	0	1	3	1	1	2	4	1	2	1	1	-	-	1	1	2	2	1	1
Ardeicola elongata	0	0	1	3	0	1	2	4	1	2	0	-	-	8	0	-	1	0	1	-
Ardeicola smithersi*	0	0	1	3	0	1	2	4	1	2	0	-	-	6	0	-	1	0	1	-
Ardeiphagus cochlearius	0	?	1	3	0	1	0	?	?	?	0	-	-	4	1	1	2	2	1	1
Auricotes affinis	0	0	1	3	2	1	2	2	1	2	0	-	-	3	0	-	2	0	1	-
Austrogoniodes waterstoni	0	0	0	3	2	0	-	2	0	-	1	3	-	-	1	1	0	0	1	-
Austrophilopterus spinosus	0	0	1	3	1	1	0	2	1	0	0	-	-	9	1	1	1	0	1	-
Bedfordiella unica	0	0	1	4	3	1	0	4	1	0	0	-	-	1	0	-	1	2	2	1
Bizarrifrons magus	0	0	1	3	1	1	0	2	1	0	0	-	-	4	0	-	1	2	1	1
Bothriometopus macrocnemis	0	0	1	3	1	1	2	5	1	?	0	-	-	1	0	-	0	2	1	1
Bovicola limbatus*	1	1	1	2	1	1	0	1	1	0	1	0	0	-	1	0	0	0	1	-
Brueelia ornatissima	0	0	1	3	0	1	0	2	1	0	0	-	-	3	0	-	1	2	1	1
Brueelia semiannulata*	0	0	1	3	0	1	0	2	1	0	0	-	-	4	0	-	2	0	1	-
Bucerocophorus pachycnemis	0	0	1	3	1	1	2	2	1	2	1	2	-	-	1	1	0	0	1	-
Buceroemersonia clarkei	0	0	1	4	1	?	?	4	?	?	1	2	-	-	1	1	2	0	1	-
***Buceronirmus* sp.**	0	0	1	3	1	0	-	4	1	1	1	2	-	-	1	1	2	0	1	-
Buceronirmus deignani	0	0	1	3	1	0	-	4	1	1	1	2	-	-	1	1	2	0	1	-

	101	102	103	104	105	106	107	108	109	110	111	112	113	114	115	116	117	118	119	120
Bucorvellus docophorus	0	0	1	3	1	1	?	2	1	0	1	2	-	-	1	1	1	2	1	1
Buerelius longiceps	0	0	1	3	1	?	?	2	1	1	0	-	-	4	0	-	0	2	1	1
Campanulotes compar*	0	0	1	3	2	1	2	2	0	-	0	-	-	3	0	-	2	0	1	-
***Capraiella* sp.**	0	0	1	4	1	1	1	4	1	1	1	2	-	-	1	1	2	0	1	-
Capraiella sabzak	0	0	1	4	1	1	1	4	1	1	1	2	-	-	1	1	2	0	1	-
Carduiceps cingulatus	0	0	1	4	3	1	0	4	1	0	0	-	-	0	0	-	1	2	1	1
Centropodiella borneoensis	0	0	?	?	?	?	?	2	1	?	?	?	?	?	?	?	?	?	?	?
Chelopistes guttatus*	0	?	1	3	2	1	2	?	?	?	0	-	-	4	0	-	2	2	2	1
Chelopistes meleagridis	0	0	1	3	2	1	2	2	1	2	1	1	-	-	1	1	2	2	2	1
Cirrophthirius testudinarius	0	0	1	3	1	1	0	4	1	0	0	-	-	4	0	-	1	2	1	1
Colilipeurus colius	0	0	1	3	1	1	0	2	1	2	0	-	-	4	0	-	2	2	1	1
Colinicola mearnsi	0	0	1	3	1	1	2	2	1	2	1	2	-	-	?	?	2	2	1	1
Coloceras damicorne*	0	0	1	3	2	1	2	2	1	2	0	-	-	3	1	?	2	0	1	-
Columbicola columbae*	0	0	1	3	1	1	0	2	1	2	0	-	-	5	0	-	0	1	2	-
Cotingacola rupicolae	0	0	1	3	1	1	0	2	1	0	0	-	-	4	0	-	1	0	1	-
Craspedonirmus colymbinus	0	0	1	4	1	1	0	4	1	0	0	-	-	0	0	-	0	2	1	1
Craspedorrhynchus platystomus*	0	0	1	3	2	0	-	2	1	2	1	3	-	-	1	1	2	0	1	-
Cuclotocephalus extraneus	0	0	1	3	3	1	2	2	1	2	0	-	-	4	0	-	2	0	1	-
Cuclotogaster madagascariensis	0	0	1	3	1	0	-	6	1	0	0	-	-	5	1	1	2	0	2	-
Cuculicola atopus*	0	0	1	3	0	1	2	2	1	2	1	5	-	-	0	-	2	0	1	-
Cuculoecus latifrons	0	0	1	3	1	1	2	2	1	2	1	2	-	-	0	-	2	2	1	1
Cummingsiella ambigua	0	0	1	3	1	1	0	2	1	0	0	-	-	4	0	-	2	2	1	1
Dahlemhornia asymmetrica	0	0	1	3	1	0	-	2	1	?	1	1	-	-	1	1	0	2	2	1
Damalinia (Damalinia) crenelata	1	1	1	2	1	1	0	1	1	0	1	0	0	-	1	0	0	0	1	-
Degeeriella rufa*	0	0	1	4	1	1	2	4	1	0	1	3	-	-	1	1	2	0	1	-
Discocorpus c. cephalosus*	0	0	0	4	1	1	2	4	1	2	0	-	-	3	0	-	2	0	1	-

	101	102	103	104	105	106	107	108	109	110	111	112	113	114	115	116	117	118	119	120
Docophoroides brevis*	0	0	1	3	1	1	3	4	1	3	1	5	-	-	0	-	0	0	0	-
Echinophilopterus protrusus	0	0	1	3	1	1	2	2	1	2	1	2	-	-	1	?	0	2	1	1
Emersoniella bracteata	0	0	1	3	1	0	-	2	0	-	1	2	-	-	0	-	2	2	1	1
Episbates pederiformis	0	0	1	4	3	1	0	4	1	0	0	-	-	1	0	-	0	2	2	1
Esthiopterum giganteum	0	0	1	3	1	1	2	2	1	2	0	-	-	1	0	-	1	2	2	1
Falcolipeurus affulgeus*	0	0	1	?	0	1	?	?	1	?	1	1	-	-	1	1	2	2	?	1
Falcolius elbeli	0	0	1	?	0	1	?	?	1	0	1	1	-	-	1	1	1	2	1	1
Felicola (F.) viverriculae*	1	1	0	0	0	1	1	0	1	1	0	-	-	1	1	0	0	0	1	-
Forficuloecus emersoni*	0	0	1	3	1	0	-	2	0	-	1	2	-	-	1	1	1	2	1	1
Formicaphagus pittasomae	0	0	1	3	1	1	0	2	1	0	0	-	-	4	0	-	2	0	1	-
Formicaricola willisi	0	0	1	3	1	1	0	4	1	0	0	-	-	4	0	-	2	0	1	-
Fulicoffula longipila	0	0	1	3	1	1	0	4	1	0	0	-	-	1	0	-	2	2	1	1
Furnariphilus pagei	0	0	1	3	1	1	0	4	1	0	0	-	-	0	0	-	1	2	1	1
Geomydoecus (G.) heaneyi	1	1	0	0	0	1	1	0	1	1	1	2	-	-	1	1	0	0	1	-
Goniocotes gallinae*	0	0	1	3	2	1	2	2	1	2	0	-	-	4	0	-	2	0	1	-
Goniodes kéleri	0	0	1	3	2	1	2	2	1	2	1	4	-	-	1	?	2	1	1	-
Goniodes pavonis	0	0	1	3	2	1	2	2	1	2	1	4	-	-	1	1	2	1	1	-
Haffneria grandis	0	0	1	3	3	1	0	4	1	0	0	-	-	2	0	-	1	2	2	1
Halipeurus pelagicus	0	0	1	4	1	1	0	4	1	0	0	-	-	1	0	-	0	2	1	0
Harrisoniella copei	0	0	1	3	3	1	0	4	0	0	0	-	-	2	0	-	1	2	2	1
Harrisoniella hopkinsi*	0	0	1	3	3	1	0	4	1	0	0	-	-	2	0	-	1	2	2	1
Heptapsogaster temporalis	0	0	0	3	2	1	2	2	1	2	0	-	-	3	0	-	2	0	1	-
Hopkinsiella clavigera	0	0	1	3	3	1	0	2	1	0	0	-	-	4	0	-	2	0	1	-
Ibidoecus plataleae*	0	0	1	3	1	1	2	2	1	2	1	2	-	-	1	1	1	0	1	-
Incidifrons fulicae*	0	0	1	3	2	1	0	2	1	0	0	-	-	4	0	-	1	2	1	1
Incidifrons transpositus	0	0	1	3	1	1	0	2	1	0	0	-	-	4	0	-	2	2	1	1

	101	102	103	104	105	106	107	108	109	110	111	112	113	114	115	116	117	118	119	120
Kelloggia coniceps	0	0	0	3	1	1	2	2	1	2	0	-	-	3	0	-	2	0	1	-
Kodocephalon latum	0	0	1	3	1	0	-	2	0	-	0	-	-	3	0	-	2	0	1	-
Labicotes guttatus	0	0	1	3	1	1	2	3	1	2	0	-	-	?	?	?	2	2	1	1
Lagopoecus affinis	0	0	1	3	1	1	2	2	1	2	1	3	-	-	0	-	1	0	1	-
Lamprocorpus hirsutus	0	0	0	4	1	1	2	4	1	0	1	2	-	-	1	1	2	0	1	-
Lipeurus caponis	0	0	1	1	1	1	0	2	1	0	0	-	-	4	0	-	2	0	2	-
Lunaceps numenii	0	0	1	4	1	1	0	4	1	0	0	-	-	4	0	-	1	2	1	1
Megaginus sordidus	0	0	0	3	1	1	2	4	1	2	0	-	-	3	0	-	2	0	1	-
Megapeostus asymmetricus	0	0	1	4	2	1	2	4	1	0	0	-	-	4	0	-	2	0	1	-
Megapodiella nakatae	0	0	1	3	0	0	-	2	0	-	1	3	-	-	0	-	1	0	1	-
Meinertzhageniella lata	0	0	1	3	1	0	-	2	1	2	1	1	-	-	1	1	2	2	1	1
Meropoecus meropis*	0	0	1	3	2	1	0	2	1	0	1	3	-	-	1	1	1	2	1	1
Meropsiella* sp.	0	0	1	3	0	1	0	2	1	0	0	-	-	4	0	-	1	2	1	1
***Mulcticola* sp.**	0	0	1	4	1	1	2	4	1	0	1	3	-	-	0	-	1	2	1	1
Naubates fuliginosus	0	0	1	4	3	1	0	4	1	0	0	-	-	1	0	-	0	2	1	0
Neophilopterus heteropygus	0	0	1	3	3	0	-	2	0	-	1	2	-	-	1	1	1	0	1	-
Neopsittaconirmus borgiolii	0	0	1	4	0	1	2	4	1	2	0	-	-	3	0	-	2	2	1	1
Nesiotinus demersus	0	0	1	5	1	0	-	2	1	2	1	5	-	-	1	?	0	0	1	-
Nothocotus subsimilis	0	0	0	4	1	0	-	4	0	-	0	-	-	3	0	-	2	0	1	-
Nyctibicola longirostris	0	0	1	?	?	?	?	2	1	2	?	?	?	?	?	?	?	?	?	?
Ornicholax robustus	0	0	0	4	1	1	2	2	1	2	0	-	-	3	0	-	2	0	1	-
Ornithobius goniopleurus	0	0	1	3	1	1	2	3	1	2	0	-	-	1	0	-	0	2	1	1
Osculotes curtus	0	0	1	3	2	1	2	2	1	0	0	-	-	0	1	1	2	0	1	-
Osculotes macropoda*	0	0	1	3	2	1	2	2	1	0	0	-	-	0	1	1	2	0	1	-
***Otidoecus* sp.**	0	0	1	3	1	1	0	4	1	0	1	2	-	-	1	0	2	2	1	1
Oxylipeurus dentatus*	0	0	1	3	1	1	0	2	1	0	0	-	-	4	0	-	2	2	1	1

	101	102	103	104	105	106	107	108	109	110	111	112	113	114	115	116	117	118	119	120
Pachyskelotes orthopleurus	0	0	1	3	2	1	2	2	1	2	0	-	-	2	0	-	2	0	1	-
Paraclisis diomedea	0	0	1	4	3	1	0	4	1	0	0	-	-	1	0	-	1	2	1	0
Paragoniocotes rotundus	0	0	1	3	0	1	2	2	1	2	0	-	-	4	1	1	2	2	1	1
Paragoniocotes venezolanus	0	0	1	3	1	1	1	4	1	0	0	-	-	4	1	1	2	2	1	1
Paroncophorus javanicus	0	0	1	3	1	1	2	4	?	?	1	2	-	-	1	1	2	2	2	1
Passonomedea hopkinsi	0	0	1	3	2	1	2	3	1	2	1	2	-	-	1	1	2	0	1	-
Pectenosoma verrucosa	0	0	0	3	1	1	2	2	1	2	0	-	-	3	0	-	2	0	1	-
Pectinopygus bassani*	0	0	1	3	1	1	2	3	1	0	0	-	-	5	0	-	1	2	1	1
Pectinopygus sulae	0	0	1	3	1	1	2	3	1	0	0	-	-	5	0	-	1	0	2	-
Pelmatocerandra setosa	0	0	1	4	3	1	0	4	1	0	0	-	-	1	0	-	1	0	1	-
Penenirmus auritus	0	0	1	4	1	1	0	4	1	0	0	-	-	9	0	-	1	2	1	1
Perineus nigrolimbatus	0	0	1	4	3	1	0	4	1	0	0	-	-	1	0	-	0	2	1	1
Pessoaiella absita*	0	0	1	3	1	1	2	2	1	0	0	-	-	0	0	-	2	0	1	-
Philoceanus garrodiae	0	0	1	4	1	1	0	4	1	0	0	-	-	7	0	-	0	2	1	1
Philopterus ornatus	0	0	1	3	1	1	2	2	1	2	1	2	-	-	1	1	2	2	1	1
Physconella kelloggi	0	0	1	3	2	1	2	2	1	2	0	-	-	3	0	-	2	0	1	-
Physconelloides cubanus	0	0	1	3	2	1	2	2	1	2	0	-	-	4	0	-	2	0	1	-
Picicola snodgrassi*	0	0	1	3	1	1	1	2	1	1	1	3	-	-	1	1	2	2	1	1
Podargoecus strigoides	0	0	1	3	1	0	-	2	0	-	1	2	-	-	1	1	2	0	1	-
Pseudocophorus perijanus	0	0	1	3	1	1	?	2	1	?	0	-	-	9	0	-	2	2	1	1
Pseudolipeurus similis*	0	0	1	3	1	1	2	4	1	2	0	-	-	1	0	-	2	2	1	1
Pseudonirmus gurlti	0	0	1	4	3	1	0	4	1	0	0	-	-	1	0	-	0	2	1	1
Pseudophilopterus hirsutus*	0	0	1	3	1	1	2	2	0	-	0	-	-	4	0	-	1	2	1	1
Psittaconirmus (P.) zinki*	0	0	1	3	1	1	1	4	1	1	0	-	-	3	1	1	2	2	2	1
Psittoecus vanzolinii*	0	0	1	3	0	0	-	2	0	-	1	6	-	-	1	1	2	0	1	-
Pterocotes aberrans	0	0	0	4	0	1	0	4	1	0	1	0	1	-	1	1	2	0	2	-

	101	102	103	104	105	106	107	108	109	110	111	112	113	114	115	116	117	118	119	120
Quadraceps coenocoryphae*	0	0	1	4	1	1	0	4	1	0	1	3	-	-	0	-	0	2	1	1
Rallicola lugens	0	0	1	4	0	1	2	4	1	0	0	-	-	0	1	1	2	0	1	-
Rhopaloceras rudimentarius	0	0	0	3	2	1	0	4	1	0	1	3	-	-	1	1	2	2	1	1
Rhynonirmus scolopacis	0	0	1	3	0	1	2	6	1	0	0	-	-	9	0	-	2	0	1	-
Rotundiceps cordatus	0	0	1	4	1	1	0	4	1	0	0	-	-	4	0	-	0	2	1	1
Saemundssonia desolata*	0	0	1	3	1	1	3	2	1	3	1	3	-	-	0	-	0	2	1	1
Saemundssonia haematopi	0	0	1	3	1	0	-	2	1	3	1	3	-	-	0	-	0	2	1	1
Splendoroffula ruwenzorornis	0	0	1	3	1	1	0	2	1	0	0	-	-	4	0	-	2	2	1	1
Strigiphilus vapidus*	0	0	1	3	2	0	-	2	0	-	1	2	-	-	1	1	2	0	1	-
Strongylocotes angulocapitis*	0	0	0	3	1	1	2	2	1	2	0	-	-	3	0	-	2	0	1	-
Strongylocotes complanatus	0	0	0	3	2	1	2	2	1	2	0	-	-	3	0	-	2	0	1	-
Struthiolipeurus struthionis*	0	0	1	3	1	1	2	4	1	2	1	1	-	-	1	1	2	2	1	1
Sturnidoecus sturni*	0	0	1	3	1	0	-	2	0	-	1	2	-	-	0	-	2	2	1	1
Syrrhaptoecus falcatus	0	0	1	3	1	?	?	2	1	2	1	3	-	-	0	-	1	2	1	0
Theresiella gemina	0	0	1	3	1	1	2	4	1	0	0	-	-	9	0	-	2	2	1	1
***Tinamotaecola* sp.**	0	0	1	3	1	1	2	2	1	2	1	4	-	-	1	1	1	2	1	1
Trabeculus schillingi	0	0	1	4	3	1	0	4	1	0	0	-	-	4	1	1	0	0	1	-
Trichodectes (T.) melis*	1	1	0	0	0	1	1	0	1	1	1	0	1	-	1	0	0	0	1	-
***Trichodopeostus* sp.**	0	0	0	4	2	1	2	?	1	2	0	-	-	3	0	-	2	0	1	-
Trichophilopterus babakotophilus	0	0	1	3	0	0	-	2	0	-	1	0	1	-	1	1	2	0	1	-
Trogoniella aequatoriale	0	0	1	4	1	1	0	4	1	0	0	-	-	5	0	-	1	0	1	-
Trogoninirmus strigilatus	0	0	1	3	1	1	1	2	1	1	1	3	-	-	1	1	1	2	1	?
Turnicola angustissimus	0	0	1	3	1	1	1	2	1	1	0	-	-	9	0	-	1	2	1	1
Turturicola salimalii	0	0	1	3	1	1	2	2	1	2	0	-	-	5	0	-	1	1	2	-
Upupicola upupae	0	0	1	4	1	0	-	4	0	-	1	3	-	-	0	-	1	0	1	-
Vernoniella guimaraesi*	0	0	1	3	1	1	2	2	1	2	0	-	-	4	0	-	2	2	2	1

	121	122	123	124	125	126	127	128	129	130	131	132	133	134	135	136	137	138
Acidoproctus hilli	3	8	-	1	-	2	1	1	8	?	?	0	0	1	0	0	0	0
Acidoproctus rostratus	3	6	-	1	-	2	1	1	6	?	?	0	0	1	0	0	0	0
Acutifrons chimango	?	?	?	?	?	?	?	?	?	?	?	?	?	?	?	?	?	0
Aegypoecus brevicollis	1	0	-	0	1	-	-	-	0	?	?	?	?	?	?	?	?	?
*Alcedoecus delphax**	0	0	-	0	0	-	-	-	0	?	?	?	?	?	?	?	?	1
*Alcedoffula alcyonae**	?	?	?	?	?	?	?	?	5	?	?	?	?	?	?	?	?	?
*Anaticola crassicornis**	3	6	-	1	-	2	0	-	6	0	1	0	0	1	0	0	0	0
*Anatoecus icterodes**	0	0	-	0	0	-	-	-	0	?	?	?	?	?	?	?	?	?
Aquanirmus australis	3	6	-	1	-	2	0	-	?	?	?	0	1	1	0	1	0	0
Archolipeurus nandu	0	0	-	0	1	-	-	-	0	?	?	?	?	?	?	?	?	0
Ardeicola elongata	2	6	-	1	-	2	0	-	6	?	?	?	?	?	?	?	?	0
*Ardeicola smithersi**	?	?	?	?	?	?	?	?	6	1	2	0	0	1	0	0	0	0
Ardeiphagus cochlearius	?	?	?	?	?	?	?	?	?	?	?	?	?	?	?	?	?	1
Auricotes affinis	?	6	-	0	0	-	-	-	?	?	?	?	?	?	?	?	?	0
Austrogoniodes waterstoni	1	1	-	0	0	-	-	-	1	?	?	0	?	?	?	?	?	0
Austrophilopterus spinosus	1	9	-	0	0	-	-	-	2	?	?	?	?	?	?	?	?	1
Bedfordiella unica	3	8	-	1	-	2	1	1	?	?	?	?	?	?	?	?	?	0
Bizarrifrons magus	?	?	?	?	?	?	?	?	5	?	?	?	?	?	?	?	?	1
Bothriometopus macrocnemis	3	6	-	1	1	2	1	0	6	?	?	?	?	?	?	?	?	0
*Bovicola limbatus**	1	4	0	0	1	-	-	-	3	1	2	0	1	0	-	0	0	0
Brueelia ornatissima	2	6	-	1	-	2	0	-	6	?	?	?	?	?	?	?	?	1
*Brueelia semiannulata**	1	4	1	0	0	-	-	-	0	0	1	1	1	0	-	1	0	1
Bucerocophorus pachycnemis	?	?	?	?	?	?	?	?	?	?	?	?	?	?	?	?	?	1
Buceroemersonia clarkei	?	?	?	?	?	?	?	?	5	?	?	?	?	?	?	?	?	0
Buceronirmus sp.	2	3	-	0	1	-	-	-	?	?	?	?	?	?	?	?	?	1
Buceronirmus deignani	?	?	?	?	?	?	?	?	?	?	?	?	?	?	?	?	?	1

	121	122	123	124	125	126	127	128	129	130	131	132	133	134	135	136	137	138
Bucorvellus docophorus	2	6	-	1	-	2	1	0	?	?	?	?	?	?	?	?	?	1
Buerelius longiceps	3	8	-	1	-	2	0	-	?	?	?	?	?	?	?	?	?	1
Campanulotes compar*	2	6	-	0	0	-	-	-	6	?	?	1	0	0	-	?	?	0
***Capraiella* sp.**	1	9	-	0	0	-	-	-	9	?	?	?	?	?	?	?	?	1
Capraiella sabzak	?	?	?	?	?	?	?	?	?	?	?	?	?	?	?	?	?	0
Carduiceps cingulatus	3	6	-	1	-	2	1	0	0	?	?	?	?	?	?	?	?	0
Centropodiella borneoensis	?	?	?	?	?	?	?	?	?	?	?	?	?	?	?	?	?	?
Chelopistes guttatus*	3	6	-	1	-	0	-	-	6	?	?	0	1	0	-	1	0	0
Chelopistes meleagridis	3	6	-	1	-	0	-	-	0	?	?	?	?	?	?	?	?	1
Cirrophthirius testudinarius	3	6	-	1	-	1	-	-	6	?	?	?	?	?	?	?	?	0
Colilipeurus colius	3	6	-	1	-	2	1	0	6	?	?	?	?	?	?	?	?	1
Colinicola mearnsi	3	6	-	1	-	0	-	-	6	?	?	?	?	?	?	?	?	0
Coloceras damicorne*	2	6	-	0	0	-	-	-	6	?	?	1	0	0	-	?	?	0
Columbicola columbae*	1	6	-	1	-	2	1	0	6	?	?	0	0	1	0	0	0	1
Cotingacola rupicolae	1	9	-	0	0	-	-	-	9	?	?	?	?	?	?	?	?	0
Craspedonirmus colymbinus	0	0	-	0	1	-	-	-	0	?	?	?	?	?	?	?	?	1
Craspedorrhynchus platystomus*	1	0	-	0	0	-	-	-	0	1	1	0	1	1	1	1	0	1
Cuclotocephalus extraneus	?	?	?	?	?	?	?	?	?	?	?	?	?	?	?	?	?	0
Cuclotogaster madagascariensis	3	6	-	1	-	2	1	0	6	1	2	0	1	0	-	0	1	0
Cuculicola atopus*	1	9	-	0	0	-	-	-	9	?	?	?	?	?	?	?	?	1
Cuculoecus latifrons	1	2	-	0	0	-	-	-	0	?	?	?	?	?	?	?	?	0
Cummingsiella ambigua	3	6	-	0	1	-	-	-	6	?	?	?	?	?	?	?	?	1
Dahlemhornia asymmetrica	0	0	-	0	0	-	-	-	0	?	?	?	?	?	?	?	?	1
Damalinia (Damalinia) crenelata	1	0	-	0	1	-	-	-	0	?	?	?	?	?	?	?	?	0
Degeeriella rufa*	2	5	-	0	0	-	-	-	5	0	0	0	1	1	0	2	0	0
Discocorpus c. cephalosus*	4	6	-	1	-	0	-	-	6	?	?	0	?	?	?	?	?	0

	121	122	123	124	125	126	127	128	129	130	131	132	133	134	135	136	137	138
Docophoroides brevis*	3	6	-	1	-	2	1	0	0	?	?	?	?	?	?	?	?	0
Echinophilopterus protrusus	1	9	-	0	1	-	-	-	9	?	?	?	?	?	?	?	?	0
Emersoniella bracteata	?	?	?	?	?	?	?	?	0	?	?	?	?	?	?	?	?	1
Episbates pederiformis	3	8	-	1	-	2	0	-	8	?	?	?	?	?	?	?	?	0
Esthiopterum giganteum	3	6	-	1	-	2	0	-	6	?	?	?	?	?	?	?	?	0
Falcolipeurus affulgeus*	1	4	0	0	1	-	-	-	5	1	2	0	1	0	-	1	0	0
Falcolius elbeli	?	?	?	?	?	?	?	?	?	?	?	?	?	?	?	?	?	1
Felicola (F.) viverriculae*	0	0	-	0	0	-	-	-	0	?	?	?	?	?	?	?	?	0
Forficuloecus emersoni*	1	2	-	0	0	-	-	-	0	?	?	?	?	?	?	?	?	1
Formicaphagus pittasomae	3	5	-	0	0	-	-	-	?	?	?	?	?	?	?	?	?	1
Formicaricola willisi	3	6	-	0	1	-	-	-	6	?	?	?	?	?	?	?	?	0
Fulicoffula longipila	3	6	-	1	-	2	1	0	6	?	?	?	?	?	?	?	?	1
Furnariphilus pagei	3	6	-	1	-	2	1	1	0	?	?	?	?	?	?	?	?	1
Geomydoecus (G.) heaneyi	0	0	-	0	0	-	-	-	0	?	?	?	?	?	?	?	?	0
Goniocotes gallinae*	2	3	-	0	0	-	-	-	4	?	?	1	0	0	-	?	?	0
Goniodes kéleri	3	6	-	1	-	0	-	-	6	?	?	1	0	0	-	2	0	0
Goniodes pavonis	3	6	-	1	-	0	-	-	6	?	?	1	0	0	-	2	0	0
Haffneria grandis	3	8	-	1	-	2	1	0	8	?	?	?	?	?	?	?	?	0
Halipeurus pelagicus	3	8	-	1	-	2	1	1	8	?	?	?	?	?	?	?	?	0
Harrisoniella copei	3	8	-	1	-	2	1	0	8	?	?	?	?	?	?	?	?	0
Harrisoniella hopkinsi*	3	8	-	1	-	2	1	0	8	?	?	?	?	?	?	?	?	0
Heptapsogaster temporalis	4	6	-	1	-	0	-	-	6	?	?	?	?	?	?	?	?	0
Hopkinsiella clavigera	?	?	?	?	?	?	?	?	?	?	?	?	?	?	?	?	?	1
Ibidoecus plataleae*	2	6	-	1	-	2	1	0	6	?	?	0	1	0	-	1	0	0
Incidifrons fulicae*	3	4	0	0	1	-	-	-	3	?	?	?	?	?	?	?	?	1
Incidifrons transpositus	?	?	?	?	?	?	?	?	6	?	?	?	?	?	?	?	?	1

	121	122	123	124	125	126	127	128	129	130	131	132	133	134	135	136	137	138
Kelloggia coniceps	4	6	-	1	-	2	0	0	6	?	?	?	?	?	?	?	?	0
Kodocephalon latum	?	?	?	?	?	?	?	?	?	?	?	?	?	?	?	?	?	0
Labicotes guttatus	?	?	?	?	?	?	?	?	?	?	?	?	?	?	?	?	?	1
Lagopoecus affinis	3	5	-	0	1	-	-	-	5	0	1	0	1	1	1	2	0	0
Lamprocorpus hirsutus	4	6	-	1	-	?	?	?	6	?	?	?	?	?	?	?	?	1
Lipeurus caponis	3	6	-	1	-	2	0	-	6	1	2	0	1	0	-	1	0	0
Lunaceps numenii	3	6	-	1	-	2	0	0	6	?	?	?	?	?	?	?	?	1
Megaginus sordidus	3	6	-	1	-	1	-	-	6	?	?	?	?	?	?	?	?	1
Megapeostus asymmetricus	3	6	-	1	-	2	0	1	6	?	?	?	?	?	?	?	?	1
Megapodiella nakatae	?	?	?	?	?	?	?	?	?	?	?	?	?	?	?	?	?	1
Meinertzhageniella lata	?	?	?	?	?	?	?	?	?	?	?	?	?	?	?	?	?	0
Meropoecus meropis*	3	4	1	0	0	-	-	-	5	?	?	?	?	?	?	?	?	1
Meropsiella* sp.	?	?	?	?	?	?	?	?	?	?	?	?	?	?	?	?	?	1
***Mulcticola* sp.**	3	6	-	1	-	0	-	-	6	?	?	?	?	?	?	?	?	0
Naubates fuliginosus	3	8	-	1	-	2	1	0	8	?	?	?	?	?	?	?	?	0
Neophilopterus heteropygus	?	?	?	?	?	?	?	?	4	?	?	?	?	?	?	?	?	1
Neopsittaconirmus borgiolii	3	6	-	1	-	0	-	-	6	?	?	?	?	?	?	?	?	0
Nesiotinus demersus	?	?	?	?	?	?	?	?	?	?	?	?	?	?	?	?	?	0
Nothocotus subsimilis	?	?	?	?	?	?	?	?	?	?	?	?	?	?	?	?	?	0
Nyctibicola longirostris	?	?	?	?	?	?	?	?	?	?	?	?	?	?	?	?	?	?
Ornicholax robustus	4	6	-	1	-	1	-	-	?	?	?	?	?	?	?	?	?	0
Ornithobius goniopleurus	3	6	-	1	-	2	1	1	6	?	?	?	?	?	?	?	?	0
Osculotes curtus	3	6	-	1	-	0	-	-	6	?	?	0	?	?	?	?	?	0
Osculotes macropoda*	3	6	-	1	-	0	-	-	6	?	?	0	?	?	?	?	?	0
***Otidoecus* sp.**	3	6	-	0	1	-	-	-	1	?	?	?	?	?	?	?	?	0
Oxylipeurus dentatus*	3	6	-	1	-	2	1	0	6	0	1	0	1	1	1	1	0	0

	121	122	123	124	125	126	127	128	129	130	131	132	133	134	135	136	137	138
Pachyskelotes orthopleurus	2	6	-	1	-	0	-	-	6	?	?	?	?	?	?	?	?	0
Paraclisis diomedea	3	8	-	1	-	2	0	-	8	?	?	?	?	?	?	?	?	0
Paragoniocotes rotundus	3	6	-	?	?	?	?	?	0	?	?	?	?	?	?	?	?	0
Paragoniocotes venezolanus	2	6	-	0	?	?	?	?	6	?	?	?	?	?	?	?	?	1
Paroncophorus javanicus	?	?	?	?	?	?	?	?	6	?	?	?	?	?	?	?	?	0
Passonomedea hopkinsi	3	6	?	?	?	?	?	?	6	?	?	?	?	?	?	?	?	0
Pectenosoma verrucosa	4	6	-	1	-	0	-	-	6	?	?	?	?	?	?	?	?	0
Pectinopygus bassani*	3	7	-	1	-	0	-	-	7	?	?	?	?	?	?	1	0	0
Pectinopygus sulae	?	?	?	?	?	?	?	?	6	?	?	?	?	?	?	1	0	0
Pelmatocerandra setosa	3	8	-	1	-	2	1	1	8	?	?	?	?	?	?	?	?	0
Penenirmus auritus	1	3	-	0	0	-	-	-	?	?	?	?	?	?	?	?	?	1
Perineus nigrolimbatus	3	8	-	1	-	2	1	0	8	?	?	?	?	?	?	?	?	0
Pessoaiella absita*	3	6	-	1	-	0	-	-	6	?	?	?	?	?	?	?	?	?
Philoceanus garrodiae	3	8	-	1	-	2	1	1	8	?	?	?	?	?	?	?	?	0
Philopterus ornatus	1	2	-	0	0	-	-	-	0	1	1	0	0	1	0	1	0	1
Physconella kelloggi	?	?	?	?	?	?	?	?	?	?	?	?	?	?	?	?	?	0
Physconelloides cubanus	2	6	-	0	1	-	-	-	6	?	?	?	?	?	?	?	?	0
Picicola snodgrassi*	?	?	?	?	?	?	?	?	?	?	?	?	?	?	?	?	?	1
Podargoecus strigoides	0	0	-	0	0	-	-	-	0	?	?	?	?	?	?	?	?	1
Pseudocophorus perijanus	?	?	?	?	?	?	?	?	?	?	?	?	?	?	?	?	?	1
Pseudolipeurus similis*	?	?	?	?	?	?	?	?	?	?	?	?	?	?	?	?	?	1
Pseudonirmus gurlti	3	8	-	1	-	2	1	-	8	?	?	?	?	?	?	?	?	0
Pseudophilopterus hirsutus*	?	?	?	?	?	?	?	?	?	?	?	?	?	?	?	?	?	0
Psittaconirmus (P.) zinki*	?	?	?	?	?	?	?	?	?	?	?	?	?	?	?	?	?	0
Psittoecus vanzolinii*	0	0	-	0	0	-	-	-	0	?	?	?	?	?	?	?	?	1
Pterocotes aberrans	4	6	-	1	-	1	-	-	6	?	?	?	?	?	?	?	?	0

	121	122	123	124	125	126	127	128	129	130	131	132	133	134	135	136	137	138
Quadraceps coenocoryphae*	0	0	-	0	1	-	-	-	0	1	1	0	0	1	0	1	0	0
Rallicola lugens	3	6	-	1	-	0	-	-	6	0	2	0	0	0	-	1	0	1
Rhopaloceras rudimentarius	4	6	-	1	-	1	-	-	6	?	?	?	?	?	?	?	?	0
Rhynonirmus scolopacis	3	6	-	1	-	1	-	-	6	?	?	?	?	?	?	?	?	0
Rotundiceps cordatus	3	6	-	1	-	2	0	-	?	?	?	?	?	?	?	?	?	1
Saemundssonia desolata*	1	4	1	0	1	-	-	-	3	1	1	0	0	1	0	0	0	1
Saemundssonia haematopi	1	4	1	0	1	-	-	-	?	1	1	0	0	1	0	0	0	1
Splendoroffula ruwenzorornis	?	?	?	?	?	?	?	?	6	?	?	?	?	?	?	?	?	0
Strigiphilus vapidus*	1	2	-	0	0	-	-	-	2	?	?	0	1	1	1	1	0	1
Strongylocotes angulocapitis*	?	?	?	?	?	?	?	?	6	?	?	0	?	?	?	?	?	0
Strongylocotes complanatus	4	6	-	1	-	2	1	0	6	?	?	?	?	?	?	?	?	0
Struthiolipeurus struthionis*	0	0	-	0	1	-	-	-	0	?	?	?	?	?	?	?	?	0
Sturnidoecus sturni*	1	2	-	0	0	-	-	-	0	1	1	0	1	0	-	1	0	1
Syrrhaptoecus falcatus	3	6	-	1	-	0	-	-	?	0	?	0	0	1	0	1	0	0
Theresiella gemina	2	6	-	1	-	1	-	-	6	?	?	?	?	?	?	?	?	0
***Tinamotaecola* sp.**	?	?	?	?	?	?	?	?	?	?	?	?	?	?	?	?	?	1
Trabeculus schillingi	3	8	-	1	-	1	-	-	8	?	?	?	?	?	?	?	?	0
Trichodectes (T.) melis*	0	0	-	0	1	-	-	-	0	1	2	?	?	?	?	1	0	0
***Trichodopeostus* sp.**	4	6	-	1	-	2	0	0	6	?	?	?	?	?	?	?	?	0
Trichophilopterus babakotophilus	0	0	-	0	1	-	-	-	0	?	?	?	?	?	?	?	?	0
Trogoniella aequatoriale	?	?	?	?	?	?	?	?	?	?	?	?	?	?	?	?	?	0
Trogoninirmus strigilatus	?	?	?	?	?	?	?	?	?	?	?	?	?	?	?	?	?	0
Turnicola angustissimus	3	6	-	1	-	0	-	-	?	?	?	?	?	?	?	?	?	1
Turturicola salimalii	?	?	?	?	?	?	?	?	?	?	?	?	?	?	?	?	?	1
Upupicola upupae	3	5	-	0	1	-	-	-	5	?	?	?	?	?	?	?	?	0
Vernoniella guimaraesi*	3	6	-	1	-	2	1	0	6	?	?	?	?	?	?	?	?	1
