# Supplementary material for: Repeated adaptive divergence of microhabitat specialization in avian feather lice
Source: BMC Biol. 2012 Jun 20;10:52. doi: 10.1186/1741-7007-10-52 (PMC3391173; doi:10.1186/1741-7007-10-52)
Supplement: Additional file 2 — Morphological character definitions. This file contains descriptions of the morphological characters and character state definitions coded in this study. [file 1741-7007-10-52-S2.PDF]

Additional File 2. Morphological character list corresponding to characters in Additional File 1. Figure numbers refer to illustrations in Smith 2001.

Head

Preantennal

1. *Adult hyaline margin*: **(0)** absent or greatly reduced, forming a thin margin running anteriorly around the marginal carina (Fig. 2B); **(1)** confined to a medial indent in the marginal carina, not evident laterally; **(2)** confined medially between or around the premarginal carina and usually continuous with the dorsal preantennal suture (Fig. 2H); **(3)** expanded and enlarged, enclosing the premarginal carina and filling the premarginal region anteriorly (Fig. 2D).
2. *Adult marginal carina*: **(0)** forms a complete thickened band running anteriorly around the preantennal region of the head (Fig. 2B); **(1)** forms a band which is interrupted laterally (partially or completely), medially (dorsally and/or ventrally) or both (Fig. 2I).
3. *Adult marginal carina (if interrupted)*: **(0)** ventrally interrupted medially, so that the marginal carina is restricted to both sides of the preantennal region ventrally but complete dorsally (Fig. 2C); **(1)** dorsally interrupted medially, so that the marginal carina is restricted to both sides of the preantennal region dorsally but complete ventrally* (Fig. 2G); **(2)** completely interrupted medially, so that the marginal carina is restricted to both sides of the preantennal region (Fig. 2E); **(3)** interrupted laterally (either partially or completely) but not interrupted medially* (Fig. 2I); **(4)** interrupted laterally (either partially or completely) and completely interrupted medially (Fig. 2A).
4. *Adult premarginal carina*: **(0)** seamlessly continuous with the postmarginal carina (Fig. 2B); **(1)** greatly reduced or absent* (Fig. 2F); **(2)** present and partially attached (not seamlessly continuous) to the postmarginal carina (Fig. 2H); **(3)** present and completely separate from the postmarginal carina (Fig. 2D).
5. *N III marginal carina*: **(0)** forms a complete thickened band running anteriorly around the preantennal region of the head; **(1)** forms a band which is interrupted laterally (partially or completely), medially (dorsally and/or ventrally) or both.
6. *N III marginal carina (if interrupted)*: **(0)** ventrally interrupted medially, so that the marginal carina is restricted to both sides of the preantennal region ventrally but complete dorsally; **(1)** dorsally interrupted medially, so that the marginal carina is restricted to both sides of the preantennal region dorsally but complete ventrally; **(2)** completely interrupted medially, so that the marginal carina is restricted to both sides of the preantennal region; **(3)** interrupted laterally (either partially or completely) but not interrupted medially*; **(4)** interrupted laterally (either partially or completely) and completely interrupted medially.
7. *N III premarginal carina*: **(0)** seamlessly continuous with the postmarginal carina; **(1)** greatly reduced or absent*; **(2)** present both dorsally and ventrally, although not seamlessly continuous with the postmarginal carina; **(3)** present and completely separate from the postmarginal carina.
8. *N II marginal carina*: **(0)** forms a complete thickened band running anteriorly around the preantennal region of the head; **(1)** forms a band which is interrupted laterally (partially or completely), medially (dorsally and/or ventrally) or both.
9. *N II marginal carina (if interrupted)*: **(0)** ventrally interrupted medially, so that the marginal carina is restricted to both sides of the preantennal region ventrally but complete dorsally; **(1)** dorsally interrupted medially, so

that the marginal carina is restricted to both sides of the preantennal region dorsally but complete ventrally; **(2)** completely interrupted medially, so that the marginal carina is restricted to both sides of the preantennal region; **(3)** interrupted laterally (either partially or completely) but not interrupted medially; **(4)** interrupted laterally (either partially or completely) and completely interrupted medially.

10. *N II premarginal carina*: **(0)** seamlessly continuous with the postmarginal carina; **(1)** greatly reduced or absent*; **(2)** present both dorsally and ventrally, although not seamlessly continuous with the postmarginal carina; **(3)** present and completely separate from the postmarginal carina.
11. *N I marginal carina*: **(0)** forms a complete thickened band running anteriorly around the preantennal region of the head; **(1)** forms a band which is interrupted laterally (partially or completely), medially (dorsally and/or ventrally) or both.
12. *N I marginal carina (if interrupted)*: **(0)** ventrally interrupted medially, so that the marginal carina is restricted to both sides of the preantennal region ventrally but complete dorsally; **(1)** dorsally interrupted medially, so that the marginal carina is restricted to both sides of the preantennal region dorsally but complete ventrally; **(2)** completely interrupted medially, so that the marginal carina is restricted to both sides of the preantennal region; **(3)** interrupted laterally (either partially or completely) but not interrupted medially; **(4)** interrupted laterally (either partially or completely) and completely interrupted medially.
13. *N I premarginal carina*: **(0)** seamlessly continuous with the postmarginal carina; **(1)** greatly reduced or absent*; **(2)** present both dorsally and ventrally, although not seamlessly continuous with the postmarginal carina; **(3)** present and completely separate from the postmarginal carina*.
14. *Adult male anterior portion of the ante-clypeus*: **(0)** unsculptured, smooth (Fig. 2J); **(1)** covered by numerous horizontal striations (Fig. 2K); **(2)** covered by numerous cresentric markings* (Fig. 2L).
15. *Adult dorsal anterior plate*: **(0)** absent (Fig. 2B); **(1)** present, always delineated anteriorly and laterally but not necessarily posteriorly (Fig. 2M-O).
16. *Adult dorsal anterior plate (if present)*: **(0)** undivided (Fig. 2M); **(1)** partially divided medially* (Fig. 2N); **(2)** completely separated medially* (Fig. 2O).
17. *Adult posterior margin of the dorsal anterior plate (if dorsal anterior plate is present)*: **(0)** not delimited, merges into dorsal sclerotization of the head capsule (Fig. 2M); **(1)** clearly delimited, separated from surrounding cuticle by the dorsal preantennal suture (Fig. 2N-Q).
18. *Adult male posterior prolongation of the dorsal anterior plate (if both the dorsal anterior plate is present and its posterior margin is delimited)*: **(0)** absent, with the posterior margin rounded or slightly pointed but not significantly developed posteriorly (Fig. 2P); **(1)** strongly developed forming a distinct posterior prolongation (Fig. 2Q).
19. *N III dorsal anterior plate*: **(0)** absent; **(1)** present but poorly developed, having the appearance of an isolated (either partially or completely) portion of the marginal carina; **(2)** present and well developed (extended posteriorly), always delineated anteriorly and laterally but not necessarily posteriorly.
20. *N II dorsal anterior plate*: **(0)** absent; **(1)** present but poorly developed, having the appearance of an isolated (either partially or completely) portion of the marginal carina; **(2)** present and well developed (extended posteriorly), always delineated anteriorly and laterally but not necessarily posteriorly.
21. *N I dorsal anterior plate*: **(0)** absent; **(1)** present but poorly developed, having the appearance of an isolated (either partially or completely) portion of the marginal carina; **(2)** present and well developed (extended

posteriorly), always delineated anteriorly and laterally but not necessarily posteriorly.

22. *N I posterior prolongation of the dorsal anterior plate (if the dorsal anterior plate is present and well developed)*: **(0)** absent. Posterior margin rounded but not developed posteriorly; **(1)** posterior margin strongly pointed but not significantly developed posteriorly*; **(2)** strongly developed forming a distinct posterior prolongation*.

23. *Adult male ventral anterior plate*: **(0)** absent; **(1)** present and well developed, forming a distinct plate which is extended posteriorly (Fig. 3J).

24. *N III ventral anterior plate*: **(0)** absent; **(1)** present and well developed, forming a distinct plate which is extended posteriorly.

25. *N II ventral anterior plate*: **(0)** absent; **(1)** present and well developed, forming a distinct plate which is extended posteriorly.

26. *N I ventral anterior plate*: **(0)** absent; **(1)** present and well developed, forming a distinct plate which is extended posteriorly.

27. *Adult dorsal preantennal suture*: **(0)** absent, or forms a discrete suture isolated from the marginal carina (Fig. 2R); **(1)** present, arising from the ends of the premarginal carina and divides the dorsal preantennal carina medially* (Fig. 2S); **(2)** developed and enlarged behind the posterior margin of the dorsal anterior plate or marginal carina, but not evident laterally and not arising from the post marginal carina (Fig. 2T); **(3)** usually arises from the ends of the postmarginal carina and surrounds the dorsal anterior plate at least laterally. Anteriorly it is continuous with the hyaline margin (Fig. 2U).

28. *Adult dorsal carina*: **(0)** absent or forms a continuous or medially broken anterior band, dorsally supporting the pre- and postmarginal carina (Fig. 2B); **(1)** expanded premarginally filling the anterior region of the head. Either not evident postmarginally or forms a thin carina above but laterally continuous with the postmarginal carina (Fig. 2V); **(2)** predominantly developed postmarginally, either absent or weakly developed premarginally (Fig. 2Z).

29. *Adult dorsal carina form (if predominantly restricted to the postmarginal region of the head)*: **(0)** restricted to the lateral margins of the head in the region of the post marginal carina, not expanded across the head*; **(1)** continuous across the head (Fig. 2W); **(2)** broken medially and variably project inward posteriorly forming two parallel bars, although the posterior projection may be weakly sclerotised (Fig. 2X); **(3)** developed medially but not forming a continuous band across the head, present although less well developed premarginally* (Fig. 2Y); **(4)** developed each side from the post marginal carina, often weakly sclerotised and not forming a definitive carina. May be continuous with the posterior margin of the dorsal anterior plate (Fig. 2Z).

30. *Adult ventral carina*: **(0)** entire, usually well marked semicircular band around the oral cavity (Fig. 3B); **(1)** interrupted medially forming a broken arch. Usually extends close to but never fuses with the marginal carina and lacks a flattened distal extension (Fig. 3C); **(2)** interrupted medially, each side possess a flattened anterior extension which approaches but does not fuse with the marginal carina (Fig. 3D); **(3)** interrupted medially, each side possess a flattened anterior extension which fuses with a marginal carina that is always interrupted, either completely or only ventrally (Fig. 3A, E); **(4)** joined to the ends of the premarginal carina but appear as bands only anteriorly (Fig. 3F); **(5)** poorly developed, apparently fused with the postmarginal carina* (Fig. 3G).

31. *N III ventral carina*: **(0)** entire, usually well marked semicircular band around the oral cavity; **(1)** interrupted

medially forming a broken arch. Usually extends close to but never fuses with the marginal carina; **(2)** interrupted medially, each side possess a flattened anterior extension which approaches but does not fuse with the marginal carina; **(3)** interrupted medially, each side possess a flattened anterior extension which fuses with a marginal carina that is always interrupted, either completely or only ventrally; **(4)** joined to the ends of the premarginal carina but appear as bands only anteriorly.

32. *N II ventral carina*: **(0)** entire, usually well marked semicircular band around the oral cavity; **(1)** interrupted medially forming a broken arch. Usually extends close to but never fuses with the marginal carina; **(2)** interrupted medially, each side possess a flattened anterior extension which approaches but does not fuse with the marginal carina; **(3)** interrupted medially, each side possess a flattened anterior extension which fuses with a marginal carina that is always interrupted, either completely or only ventrally; **(4)** joined to the ends of the premarginal carina but appear as bands only anteriorly*.
33. *N I ventral carina*: **(0)** entire, usually well marked semicircular band around the oral cavity; **(1)** interrupted medially forming a broken arch. Usually extends close to but never fuses with the marginal carina; **(2)** interrupted medially, each side possess a flattened anterior extension which approaches but does not fuse with the marginal carina; **(3)** interrupted medially, each side possess a flattened anterior extension which fuses with a marginal carina that is always interrupted, either completely or only ventrally; **(4)** joined to the ends of the premarginal carina but appear as bands only anteriorly*.
34. *Adult pulvinus*: **(0)** single lobe attached to the ventral carina (Fig. 3H); **(1)** divided into two lateral lobes (Fig. 3I).
35. *Adult male coni*: **(0)** undeveloped, marked only by a slight bump before the anterior margin of the antennal socket; **(1)** developed, forming a significant blunt or pointed process extending laterally from the margin of the head. May be hyaline, sclerotised or both.
36. *Adult female conus*: **(0)** undeveloped, marked only by a slight bump before the anterior margin of the antennal socket (Fig. 4A); **(1)** developed, forming a significant blunt or pointed process extending laterally from the margin of the head. May be hyaline, sclerotised or both (Fig. 4J-L).
37. *Adult female coni morphology (if coni are developed)*: **(0)** shorter or as long as the scape (Fig. 4K, L); **(1)** longer than the scape (Fig. 4J).
38. *Adult trabecula*: **(0)** absent; **(1)** present* (Fig. 4B, L).
39. *Adult transverse carina*: **(0)** absent; **(1)** present, either complete or broken medially (Fig. 2A').
40. *Adult marginal pulvinal band*: **(0)** absent; **(1)** present as a distinct band separate from the torma (Fig. 3H, L [not shaded]); **(2)** fused at either end to the ventral carina (Fig. 3K).
41. *Adult torma*: **(0)** absent; **(1)** present and distinctly separate from the marginal pulvinal band (Fig. 3H, L [not shaded]).
42. *Adult pulvinus size*: **(0)** small, unmodified; **(1)** greatly expanded filling the ventral preantennal region (Fig. 3L).
43. *N III marginal pulvinal band*: **(0)** absent; **(1)** present as a distinct band separate from the torma; **(2)** fused at either end to the ventral carina.
44. *N III torma*: **(0)** absent; **(1)** present and distinctly separate from the marginal pulvinal band.

Antennal

45. *Male antennal segments*: **(0)** not significantly heteromorphic; **(1)** significantly heteromorphic (Fig. 4E, G-I [Not shaded]).

46. *Male scape shape based on elliptic Fourier analysis*: **(0)** partition 0, short and rounded; **(1)** partition 1, elongated with lateral process; **(2)** partition 2, elongated without lateral process.

47. *Process on anterior lateral margin of the male scape (if antennal segments are significantly heteromorphic)*: **(0)** absent (Fig. 4F, G, I); **(1)** present (Fig. 4E, H).

48. *5-6 microsetae in a row across the length of the male scape*: **(0)** absent (Fig. 4E, F, H, I); **(1)** present (Fig. 4G).

49. *Male and female pedicel and flagellum / flagellomeres*: **(0)** not covered in numerous fine microsetae (Fig. 4E, G-I); **(1)** covered in numerous fine microseta (Fig. 4F).

50. *Male flagellomeres*: **(0)** unfused (Fig. 4E, H, I); **(1)** fused (Fig. 4F, G).

51. *First flagellomere shape (if antennal segments are significantly heteromorphic and unfused)*: **(0)** unmodified; **(1)** derived, not forming a simple flagellomere (Fig. 4E, H, I).

52. *Subterminal attachment of flagellomeres II and III (if antennal segments are significantly heteromorphic and unfused)*: **(0)** absent (Fig. 4F-I); **(1)** present (Fig. 4E).

53. *Apical compression of flagellomeres II and III (if antennal segments are significantly heteromorphic and unfused)*: **(0)** absent (Fig. 4E-H); **(1)** present (Fig. 4I).

Postantennal

54. *Gular plate*: **(0)** absent; **(1)** present forming a distinct sclerotised region on the ventral surface of the head, although not necessarily clearly delimited.

55. *Gular plate form (if present)*: **(0)** not delimited, only evident as a distinct sclerotised region on the ventral surface of the head (Fig. 3M); **(1)** clearly distinct anteriorly but laterally and posteriorly more or less continuous with the ventral sclerotization of the head (Fig. 3N); **(2)** clearly delimited anteriorly and laterally but not necessarily posteriorly (Fig. 3O).

56. *Anterior margin of the gular plate (if present and delimited)*: **(0)** smoothly rounded (Fig. 3P); **(1)** pointed (Fig. 3A, N, O, Q).

57. *Pointed anterior margin of the gular plate (if present, delimited and has a pointed anterior margin)*: **(0)** completely tapered to a distinct point; **(1)** pointed medially on an otherwise flat anterior margin (Fig. 3N, O); **(2)** pointed medially on an otherwise rounded anterior margin (Fig. 3A, Q).

58. *Marginal temporal carina*: **(0)** thin, forming a more or less evenly thick band around the temples (Fig. 2B'); **(1)** thickened and enlarged, forming a band of uneven thickness around the temporal margin (Fig. 2C').

59. *Postocular nodus*: **(0)** absent or weakly developed, identifiable as a slight expansion of the marginal temporal carina; **(1)** well developed and enlarged (Fig. 2B', C').

60. *Female ocular setal condition*: **(0)** thorn-like or normal microseta; **(1)** normal or macroseta (not thorn-like); **(2)** thorn-like macroseta*.

61. *Female postocular setal condition*: **(0)** thorn-like or normal microseta; **(1)** normal or macroseta (not thorn-like)*; **(2)** thorn-like macroseta.

62. *Female postocular setal position*: **(0)** on the lens of the eye; **(1)** not on the lens of the eye.

63. *Female marginal temporal setal number*: **(0)** three*; **(1)** four; **(2)** five; **(3)** six or more*.

64. *Female dominant marginal temporal setae (for taxa with 5 MTS)*: **(0)** all subordinate microsetae; **(1)** MTS 1

and 3 dominant; **(2)** MTS 2 and 3 dominant; **(3)** MTS 1, 2 and 3 dominant; **(4)** MTS 1, 2, 3 and 5 dominant*; **(5)** MTS 3 dominant; **(6)** MTS 2 and 5 dominant*; **(7)** MTS 3 and 4 dominant*; **(8)** MTS 1 - 4 dominant; **(9)** MTS 1 - 5 dominant.

65. *Female MTS patterns (where MTS 1 and 3 are dominant)*: **(0)** MTS 1 and 3 dominant microsetae, MTS 2, 4, and 5 microsetae; **(1)** MTS 1 and 3 dominant normal or macrosetae, MTS 2, 4, and 5 microsetae.

66. *Female MTS patterns (where MTS 1, 2 and 3 are dominant)*: **(0)** MTS 1, 2, and 3 dominant normal or macroseta, MTS 4 and 5 microsetae; **(1)** MTS 1 thorn-like macroseta, MTS 2 and 3 dominant normal or macroseta, MTS 4 and 5 microsetae*; **(2)** MTS 1 developed microseta, MTS 2 thorn-like macroseta, MTS 3 dominant macroseta, MTS 4 and 5 thorn-like microseta*.

67. *Female MTS patterns (where MTS 3 is dominant)*: **(0)** MTS 3 dominant microseta; MTS 1, 2, 4 and 5 subordinate microsetae; **(1)** MTS 3 dominant normal or macroseta; MTS 1, 2, 4 and 5 subordinate microsetae.

68. *Female MTS patterns (where MTS 1 - 5 are dominant)*: **(0)** MTS 1 dominant microseta, MTS 3 normal or macrosetae; MTS 2, 4 and 5 thorn-like macrosetae; **(1)** MTS 3 normal or macrosetae, MTS 1, 2, 4, and 5 thorn-like macrosetae*; **(2)** MTS 1 - 4 normal or macroseta, MTS 5 thorn-like macroseta*.

Thoracic

69. *Anterior prothoracic setal distribution*: **(0)** absent or confined to the anterior margin of the prothorax without a disjunct distribution (Fig. 7A); **(1)** not confined to the extreme anterior margin, distribution disjunct (Fig. 7C).

These setae are not to be confused with the two microsetae present on each cervical sclerite.

70. *Rhombic sclerite shape*: **(0)** small discrete oblong, rhombic or rounded sclerite, may be weakly developed or only delimited anteriorly (Fig. 7F); **(1)** medium to large sclerite, may not be strongly delimited (Fig. 7G).

71. *Mesothoracic spiracle position*: **(0)** ventral sublateral without an enlarged atrium; **(1)** more or less pleural, not sublateral, without extension on a slight lateral protuberance or with an enlarged atrium (Fig. 8F); **(2)** more or less plural with an enlarged atrium and thickening of the atrial walls (Fig. 8G); **(3)** extended out on a slight lateral protuberance of the prothorax without an enlarged atrium (Fig. 8H).

72. *Female lateral and/or posterior prothoracic setal arrangement*: **(0)** 0+0*; **(1)** 1+1, may be lateral or sublateral (Fig. 7B); **(2)** 2+2 (Fig. 7C); **(3)** 4+4 or 5+5* (Fig. 7D); **(4)** numerous setae along the lateral and posterolateral margin becoming sub-posterior medially (Fig. 7E).

73. *Female lateral and/or posterior prothoracic setal position (if a 1+1 arrangement is present)*: **(0)** single setal pair on the lateral or posterior margin, not sublateral; **(1)** single sublateral setal pair slightly anterior to the posterior margin of the prothorax (Fig. 7B).

74. *Proepimeron proximal development*: **(0)** more or less blunt ended (usually rounded) (Fig. 8K); **(1)** expanded (occasionally may be fused across the middle to form a single medial plate) (Fig. 8A-E).

75. *Direction of proximal development of the proepimeron (if expanded)*: **(0)** more or less equally expanded anteriorly and posteriorly (Fig. 8B); **(1)** predominantly anteriorly (towards / into the prothorax) (Fig. 8C); **(2)** predominantly posteriorly (towards the abdomen) (Fig. 7A); **(3)** predominantly posteriorly (towards the abdomen) but partially fused anteriorly (Fig. 8D); **(4)** completely fused medially* (Fig. 8E).

76. *Small medial sclerite between or beneath the proximal ends of the proepimeron*: **(0)** absent; **(1)** present (Fig. 8A).

77. *Meso-metasternal plate*: **(0)** absent; **(1)** present (Fig. 8I, J).

78. *Second sternal plate*: **(0)** absent; **(1)** present (Fig. 8I, J).

79. *Cuticular bridge between the meso-metasternal plate and the 2nd sternal plate (if both the meso-metasternal plate and 2nd sternal plate are present)*: **(0)** absent (Fig. 8I); **(1)** present (Fig. 8J).

80. *Ventral pterothoracic setae in the region of the meso- to metasternal plate*: **(0)** absent; **(1)** present (Fig. 8K).

81. *Female ventral pterothoracic setal arrangement in the region of the meso- to metasternal plate (if present)*: **(0)** single setal pair (may be meso- or metasternal); **(1)** mesosternal setae absent, row of four or five metasternal setae present*; **(2)** pair of mesosternal and a pair of metasternal setae present; **(3)** single pair (rarely 3) mesosternal setae and at least 3 (usually 4 although sometimes 5, 6 or 8) metasternal setae.

82. *Attachment of the 2nd and 3rd pairs of legs*: **(0)** sternocoxal, approximately 95% of the coxal surface present beneath the thorax and abdomen (Fig. 8L); **(1)** sterno- pleurocoxal, approximately 30-70% of the coxal surface present beneath the thorax and abdomen (Fig. 8M); **(2)** pleurocoxal, approximately 5-15% of the coxal surface present beneath the thorax and abdomen (coxa attached at the corners of the pterothorax) (Fig. 8N).

83. *Pteronotum*: **(0)** undivided (Fig. 7A, N, Q, R); **(1)** divided medially (Fig. 7O, P, S).

84. *Pterothoracic lateral margins*: **(0)** more or less parallel (Fig. 7R); **(1)** divergent (Fig. 7O).

85. *Pterothoracic lateral margins (if parallel)*: **(0)** without setae (Fig. 7R); **(1)** with setae on the lateral margins.

86. *Pterothoracic posterior margin*: **(0)** more or less flat (Fig. 7R); **(1)** curved or distinctly 'v' shaped (Fig. 7O-Q).

87. *Female medial pair of microseta on the first third of the pterothorax*: **(0)** absent (Fig. 7Q); **(1)** present (Fig. 7R).

88. *Female medial pair of setae on the last third of the pterothorax*: **(0)** absent (Fig. 7R); **(1)** present.

89. *Female pterothoracic trichoid seta*: **(0)** absent; **(1)** present, must be a typical trichoid seta emanating from a distinct ventral or lateral pit (Fig. 7H).

90. *Female pterothoracic thorn-like seta, associated with, although not dependent upon the presence of a trichoid seta*: **(0)** absent; **(1)** present (Fig. 7H).

91. *Female setal pattern on the lateral and posterior margin of the pterothorax, excluding the trichoid setae or its setal equivalent and its associated thorn-like setae*: **(0)** rarely three, four or more commonly at least five well spaced setae forming a complete or broken row along the margin both sides (Fig. 7O, Q, S); **(1)** mainly clustered, not forming a complete or broken row along the margin (Fig. 7N, R); **(2)** clustered single outer pair and three or four inner setae in a row both sides (Fig. 7P).

92. *Female setal pattern on the posterior margin of the pterothorax, excluding the trichoid setae or its setal equivalent and its associated thorn-like seta (if present as a complete or broken row along the margin)*: **(0)** complete, setae in some cases may be well spaced (Fig. 7O, Q); **(1)** discontinuous, setae absent within the submedian and median region of the pterothorax (Fig. 7S).

93. *Female setal patterns on the lateral and posterior margin of the pterothorax, excluding the trichoid setae or its setal equivalent and its associated thorn-like seta (if clustered along the margin)*: **(0)** loosely grouped on the posterolateral margin in various arrangements, not closely associated; **(1)** '2,2+2,2' arrangement (rarely '2,2,1+1,2,2') (Fig. 7N); **(2)** '3+3' arrangement (Fig. 7I); **(3)** '2,3+3,2', '3,2+2,3' or '3,3+3,3' (Fig. 7J); **(4)** '4+4' arrangement (rarely '4,1+1,4') (Fig. 7K); **(5)** '5+5' arrangement* (Fig. 7L); **(6)** '7+7' arrangement* (Fig. 7M).

94. *Female setal patterns on the lateral and posterior margin of the pterothorax, excluding the trichoid setae or its setal equivalent and its associated thorn-like seta (if clustered and present as a 2,2+2,2 or 2,2,1+1,2,2 arrangement)*: **(0)** 2,2+2,2; **(1)** 2,2,1+1,2,2 (Fig. 7N).

Abdominal

95. *Adult male tergum I*: **(0)** absent or fused to tergum II; **(1)** present, identified by a small isolated and weakly sclerotised tergite (Fig. 10).

96. *Adult female submedian to median dorsal setae on abdominal segment II*: **(0)** absent ; **(1)** single pair or row (Fig. 11B); **(2)** two pairs or rows, one behind the other (Fig. 11D); **(3)** three or more pairs or rows, each more or less behind each other (Fig. 11C).

97. *Adult female abdominal segment II*: **(0)** not deeply embedded within abdominal segment III; **(1)** deeply embedded within abdominal segment III (Fig. 11E).

98. *Adult female lateral and/or sublateral setae on abdominal segment II*: **(0)** absent; **(1)** present, may be lateral, dorsal and/or ventral.

99. *Adult female lateral and/or sublateral setal type and arrangement on abdominal segment II (if present)*: **(0)** micro, normal or macroseta or setal pair, present each side on the posterolateral or sublateral margin (Fig. 11F); **(1)** numerous microsetae covering the lateral margins of the pleurites or tergopleurites (Fig. 11G); **(2)** three or four clustered thorn-like setae* (Fig. 11H); **(3)** two or more short blade-like setae, may be present as part of a continuous setal row (Fig. 11I); **(4)** three or four short normal or microsetae present both sides on the lateral and dorsal sublateral margin of the pleurites* (Fig. 11J).

100. *Abdominal spiracle number*: **(0)** six pairs; **(1)** less than six pairs.

101. *Atria of abdominal spiracles*: **(0)** small (Fig. 11O); **(1)** greatly enlarged (Fig. 11P).

102. *Abdominal male lateral flecks (sensu Moreby, 1978)*: **(0)** absent ; **(1)** present (see Figs 7-10 in Moreby, 1978).

103. *Cell shaped cuticular sculpturing on the dorsal and/or ventral abdominal surface*: **(0)** absent in all stadia examined; **(1)** present, either dorsally and/or ventrally in any stadium (Fig. 11Q & 13 D).

104. *Adult female pleural and tergal abdominal plate sclerotisation. Segment IV to VII ONLY*: **(0)** absent (completely unsclerotised) or greatly reduced, limited to dorsal tergites; **(1)** restricted to pleurites that extend no more than sublaterally over the dorsal surface* (Fig. 12G); **(2)** restricted to separate pleurites and tergites; **(3)** restricted to tergopleurites that are unfused medially; **(4)** restricted to tergopleurites that are fused medially.

105. *Adult female pleural abdominal ribs. Segment IV to VII ONLY*: **(0)** absent or restricted to thin folds over the lateral side of the abdomen (Fig. 11K); **(1)** enlarged although not greatly expanded (Fig. 11L); **(2)** greatly expanded with an enlarged pleural knot (Fig. 11M); **(3)** greatly expanded with an anterior and posterior process (Fig. 11N).

106. *Adult female sternal abdominal plates. Segment IV to VII ONLY*: **(0)** absent ; **(1)** present, may be medially fused forming a single sternite or broken forming paired sternites on each segment.

107. *Adult female sternal plate morphology (if present). Segment IV to VII ONLY*: **(0)** sclerotised and complete, fused medially across the abdomen; **(1)** unsclerotised and complete, fused medially across the abdomen; **(2)** separate, present both sides of the abdomen; **(3)** separate in the anterior segments but complete in the posterior segments.

108. *Adult male pleural and tergal abdominal plate sclerotisation. Segment IV to VII ONLY*: **(0)** absent (completely unsclerotised) or greatly reduced, limited to dorsal tergites (Fig. 12A); **(1)** restricted to separate pleurites and tergites (Fig. 12B); **(2)** restricted to tergopleurites that are unfused medially (Fig. 12C); **(3)** restricted to tergopleurites that are unfused medially in segments IV and possibly V but medially fused in the remaining

segments* (Fig. 12D); **(4)** restricted to tergopleurites that are fused medially (Fig. 12E); **(5)** restricted to tergopleurites that are unfused medially and accompanied by a separate isolated medial tergite on the posterior region of each segment (Fig. 11A); **(6)** restricted to tergopleurites that are fused medially and accompanied by a separate isolated medial tergite on the posterior margin of the fused tergopleurite* (Fig. 12F).

109. *Adult male sternal abdominal plates. Segment IV to VII ONLY*: **(0)** absent ; **(1)** present, may be medially fused forming a single sternite or broken forming paired sternites on each segment.

110. *Adult male sternal plate morphology (if present). Segment IV to VII ONLY*: **(0)** complete, fused medially across the abdomen (Fig. 13A); **(1)** unsclerotised and complete, fused medially across the abdomen (Fig. 13B); **(2)** separate, present both sides of the abdomen (Fig. 13C); **(3)** separate in the anterior segments but complete in the posterior segments (Fig. 13D).

111. *Adult female dorsal abdominal setal rows. Segment III to VI ONLY*: **(0)** absent; **(1)** present, may be discontinuous and with at least four or more setal pairs present per segment.

112. *Adult female dorsal abdominal setal row arrangement (if present). Segment III to VI ONLY*: **(0)** present laterally to intermedially, usually as microsetae or short small normal setae. May also be present more medially and possibly continuous across the abdomen; **(1)** continuous sublateral to intermediate microsetal or short normal setal rows; **(2)** continuous sublateral to intermediate normal or macrosetal rows; **(3)** discontinuous, single sublateral or intermediate normal or macroseta and a submedian normal or macrosetal row; **(4)** discontinuous, two or three sublateral or intermediate normal or macroseta and a submedian normal or macrosetal row; **(5)** continuous submedian normal setal row*.

113. *Adult female dorsal abdominal setal type (if present in rows, laterally to intermedially). Segment III to VI ONLY*: **(0)** short small microsetae; **(1)** normal setae*.

114. *Adult female dorsal abdominal setae (if not in rows). Segment III to VI ONLY*: **(0)** median normal or macrosetal pairs only; **(1)** median microsetal pairs only; **(2)** median and submedian or intermediate microsetal pairs; **(3)** intermediate setal pairs regardless of type on the posterior margin of each segment, no median setae; **(4)** median and intermediate setal pairs, may be normal or macrosetae; **(5)** submedian and median pairs only*; **(6)** median blade-like and intermediate normal setal pairs with submedian pairs on segments VII and VIII*; **(7)** sublateral pairs only*.

115. *Adult female ventral abdominal setal rows. Segment III to VI ONLY*: **(0)** absent; **(1)** present, not necessarily complete but with at least 4 setal pairs per segment.

116. *Adult female ventral abdominal setal type (if present in rows). Segment III to VI ONLY*: **(0)** microsetae, may be discontinuous sublateral or intermedially; **(1)** not microsetae, either normal setae or macrosetae. May be discontinuous sublateral or intermedially.

117. *Adult female trichoid seta on the posterolateral margin of abdominal segment VIII*: **(0)** absent, not identifiable from the other setae on the posterolateral margin of this segment (Fig. 11R); **(1)** present and identifiable although not emanating from a distinctive pit (Fig. 11S); **(2)** present with a distinct trichoid seta emanating from a distinct and well developed pit (Fig. 11T).

118. *Adult female dorsal abdominal plates of the terminal abdominal segments*: **(0)** fused, giving the appearance that the segment forms a single unit (Fig. 11U); **(1)** terminal division absent, no evidence of a terminal XI tergite although divisions are present between the lateral portions of the fused IX and X tergite (Fig. 11A' & B'); **(2)** terminal division present separating the segment into its anterior (tergite IX and X) and posterior

(tergite XI) regions, additional divisions within these regions may also be present (Fig. 11W).

119. *Adult female fused IX and X tergites, not necessarily delimited from tergite XI*: **(0)** divided into separate pleurites and a single medial tergite* (Fig. 11Y); **(1)** fused medially forming a single tergopleural plate (Fig. 11V); **(2)** divided into tergopleurites, no medial tergite.

120. *Adult female XI tergite (if present and delimited from tergite IX and X)*: **(0)** medially fused forming a single terminal tergal plate (Fig. 11X); **(1)** medially divided into two terminal tergites (Fig. 11Z).

121. *N III dorsal abdominal plates of abdominal segment II*: **(0)** absent, unsclerotised; **(1)** pleurites only; **(2)** pleurites and isolated tergites; **(3)** tergopleurites.

122. *N III dorsal abdominal plate sclerotisation. Segments III to VIII ONLY*: **(0)** absent (completely unsclerotised) although a few pleurites may be weakly delimited (Fig. 14A); **(1)** restricted to pleurites in abdominal segment III, all other segments not bearing sclerotised plates* (Fig. 14B); **(2)** restricted to pleurites in abdominal segments III and IV only, all other segments not bearing sclerotised plates (Fig. 14C); **(3)** restricted to pleurites in abdominal segments III to VII only, segment VIII not bearing sclerotised plates* (Fig. 14D); **(4)** restricted to pleurites in abdominal segments III to VIII only, no evidence of any separate tergites (Fig. 14F); **(5)** pleurites and tergites separate and sclerotised although tergites absent from one or more posterior segments (Fig. 14E); **(6)** pleurites and tergites separate and sclerotised (Fig. 14G); **(7)** tergopleural plates that are only connected to each other along their posterior margins except in segment VIII which possesses separate tergites and pleurites (Fig. 14H); **(8)** tergopleurites unfused medially (Fig. 14I).

123. *N III abdominal pleurite shape (if abdominal sclerotisation is restricted to pleurites in segments III to VIII)*: **(0)** thin similar sized elongated plates bordering the edges of each segment (Fig. 14F); **(1)** rounded plates on the lateral margin within each segment which get significantly smaller in each proceeding segment.

124. *N III terminal abdominal segments*: **(0)** undifferentiated by sclerotised abdominal plates (Fig. 14A-F); **(1)** differentiated either partially or completely by sclerotised abdominal plates (Fig. 14G-I).

This character refers to the differentiation of segments IX, X and XI only.

125. *N III terminal abdominal segments (if undifferentiated by sclerotised abdominal plates)*: **(0)** completely undivided, no evidence to intersegmental division in the integument of the terminal segment (Fig. 14B-D); **(1)** divided, with evidence of an intersegmental division via folds in the integument of the terminal segments (Fig. 14A, E & F).

126. *N III terminal abdominal segments (if differentiated by sclerotised abdominal plates)*: **(0)** delimited by an isolated pair of tergal and/or sternal plates only (Fig. 14H); **(1)** delimited by a single tergopleural plate (a pair of sternites may also be evident)*; **(2)** differentiated into two or three rows of plates (Fig. 14G & I).

127. *N III terminal abdominal segments (if differentiated into two or three rows of sclerotised abdominal plates)*: **(0)** two rows present (Fig. 14G); **(1)** three rows present (Fig. 14I).

128. *N III segment X (if terminal segments IX, X and XI are differentiated into three rows of sclerotised abdominal plates)*: **(0)** medially separated into two isolated tergites or tergopleurites; **(1)** medially fused forming a single tergite or tergopleurite across the segment (Fig. 14I).

129. *N II abdominal plate sclerotisation. Segments III to VIII ONLY*: **(0)** absent (completely unsclerotised) although a few pleurites may be weakly delimited; **(1)** restricted to pleurites in abdominal segment III, all other segments not bearing sclerotised plates*; **(2)** restricted to pleurites in segments III and IV only, all other segments not bearing sclerotised plates*; **(3)** restricted to pleurites in segments III - V, absent in segments VI -

VIII; **(4)** restricted to pleurites in segments III - VI, absent in segments VII and VIII*; **(5)** restricted to pleurites in segments III - VIII, no evidence of any separate tergites; **(6)** pleurites and tergites separate and sclerotised; **(7)** tergopleural plates that are only connected to each other along their posterior margins except in segment VIII which possesses separate tergites and pleurites; **(8)** tergopleurites unfused medially.

Internal Genitalia

130. *Testes*: **(0)** weakly bilobed; **(1)** strongly bilobed (Fig. 15A, C).

131. *Distal termination of the testes*: **(0)** blunt without a short process or flagellate extension* (Fig. 15B); **(1)** blunt with a short process which may or may not carry a short flagellate extension (Fig. 15A, C); **(2)** flagellate but not carried by a short process (Fig. 15D).

132. *Vesicula seminalis*: **(0)** forms a more or less single structure which appears internally divided medially (Fig. 15A); **(1)** separated distally but apparently fused proximally with the vas deferens entering the separated organ before it becomes fused (Fig. 15G); **(2)** forms two distinct separate structures*.

133. *Subdivisions within each vesicula*: **(0)** absent (Fig. 15E); **(1)** present, each vesicula must be clearly lobed internally (Fig. 15F).

134. *Lateral lobe on each vesicula*: **(0)** absent; **(1)** present (Fig. 15A, E, H, I, K).

135. *Lateral lobe size on each vesicula (if present)*: **(0)** small (Fig. 15E, H, I); **(1)** large, lying along more than half the length of each vesicula. (Fig. 15A, K).

136. *Ductus ejaculatorius, joining each vesicula at the base*: **(0)** forms a short simple tube only slightly longer than the length of the basal apodeme (Fig. 15I); **(1)** greatly swollen, at least 1½ x length of the basal apodeme (Fig. 15J); **(2)** long and coiled (Fig. 15K).

137. *Unpaired diverticulum at the junction of the vesicula seminalis and ductus ejaculatorius*: **(0)** absent (Fig. 15A, H-K); **(1)** present* (Fig. 15L).

138. *Genital chamber calyx*: **(0)** unsclerotised, extremely hyaline or not visible ; **(1)** sclerotised and striated, clearly visible in cleared slide mounted specimens (Fig. 16A, B).
