# Supplementary material for: Repeated adaptive divergence of microhabitat specialization in avian feather lice
Source: BMC Biol. 2012 Jun 20;10:52. doi: 10.1186/1741-7007-10-52 (PMC3391173; doi:10.1186/1741-7007-10-52)
Supplement: Additional file 3 — GenBank accession numbers. This file contains GenBank accession numbers for the sequences used in this study. [file 1741-7007-10-52-S3.PDF]

Additional File 3. GenBank Accession numbers for species in study.

Louse Species	Wingless	COI	EF1	Voucher code
Alcedoecus alatoclypeatus	JX121642	AY314807	AF545775	Alsp.Hamal.1.16.2001.11
Alcedoffula duplicata	N/A	JX121669	JX121682	Afdup.3.16.2001.10
Anaticola crassicornis	GU569394	GU569311	AF320353	Ancra.10.17.2000.3
Anatoecus icterodes	N/A	JX121671	JX121684	Atsp.Andis.9.27.2000.6
Quadraceps punctatus	N/A	JX121678	JX121692	Qupun.3.24.2001.8
Saemundssonia lari	JX121663	AF348865	AY149435	Salar.4.7.1999.12
Ardeicola expallidus	JX121643	JX121670	JX121683	Arexp.9.27.2000.8
Ibidoecus bisignatus	JX121657	AY314817	AY314836	Ibbis.9.27.2000.3
Campanulotes compar	JX121645	AF545681	AF278671	Cabid.6.29.1998.2
Coloceras sp.	JX121646	AF278646	AF278664	Ccsp.Phleu.7.1.1999.5
Columbicola columbae	JX121649	AF385003	AF320386	Cocol.6.29.1998.1
Cuculicola atopus	JX121650	AF444856	AF320388	Cuato.1.27.1999.4
Vernoniella bergi	N/A	AY314824	AY314844	Veber.10.17.2000.7
Craspedorrhynchus hirsutus	JX121647	AF545690	AF545780	Cfhir.1.15.2000.6
Degeeriella carruthi	N/A	AF444860	AF447196	Dgcar.9.8.1999.7
Falcolipeurus marginalis	JX121653	AY314814	AY314833	Famar.6.9.2001.4
Chelopistes sp.	JX121648	JX121674	JX121687	Chsp.Orcan.11.10.2001.9
Goniocotes chrysocephalus	JX121656	HQ332829	HQ332891	Gosp.Phcol.11.10.2001.2
Oxylipeurus chiniri	N/A	AF545739	AF320437	Oxchi.1.27.1999.6
Incidifrons transpositus	N/A	AF545719	AF545790	Intra.1.15.2000.9
Meropoecus sp.	JX121658	JX121675	JX121689	Mrsp.Megul.3.24.2001.11
Meropsiella sp.	N/A	JX121672	JX121685	Brsp.Megul.3.24.2001.10
Osculotes curta	N/A	AF3488858	AF348660	Oscur.10.5.1999.2
Pessoaiella absita	JX121668	JX121681	JX121695	Wiabs.10.5.1999.2
Brueelia ornatissima	JX121644	JX121673	JX121686	Brsp.Moate.3.24.2001.3
Sturnidoecus sp.	JX121666	JX121680	JX121694	Snsp.Tugra.10.16.2002.1
Pectinopygus bassani	JX121660	DQ314507	DQ314517	Pgbas.11.10.2001.13
Picicola porisma	JX121661	AF444867	AF447202	Pipor.10.17.2000.5
Docophoroides brevis	JX121652	AF396547	AF320394	DOCbrev1 (from GenBank)
Harrisoniella densa	N/A	AF396567	AF320410	HARdensa (from GenBank)
Forficuloecus palmai	JX121655	EU669828	JX121688	Ffpal.11.22.2001.14
Psittaconirmus forficuloides	JX121659	JX121676	JX121690	Pcfor.10.16.2002.8
Psittoecus eos	JX121662	JX121677	JX121691	Qkeos.5.16.2002.5
Strigiphilus crucigerus	JX121667	AF545767	AF320468	Stcru.1.27.1999.10
Struthiolipeurus nandu	JX121665	AF545768	HQ124326	Slnan.2.4.2002.4
Discocorpus mexicanus	JX121651	AF545695	AF320393	Dimex.1.27.1999.8
Pseudolipeurus similis	N/A	AF545752	AF320455	Pssim.1.27.1999.5
Pseudophilopterus hirsutus	N/A	AF545755	AF545799	Qshir.2.1.2000.11
Strongylocotes orbicularis	JX121664	HQ332847	HQ332918	Sgorb.11.10.2001.10
**Outgroups**				
Bovicola bovis	N/A	AF545680	AF320370	Bobov.2.4.2002.2
Felicola subrostratus	JX121654	AF545700	AF320398	Fesub.2.4.2002.7
Stachiella larseni	N/A	JX121679	JX121693	Shlar.3.16.2001.4
Trichodectes octomaculatus	N/A	AY314823	AY314843	Tdoct.2.4.2002.1
